# Care-seeking practices for sick neonates: Findings from cross-sectional survey in 14 rural sub-districts of Bangladesh

**DOI:** 10.1371/journal.pone.0204902

**Published:** 2018-09-27

**Authors:** Suman Kanti Chowdhury, Sk Masum Billah, Shams El Arifeen, Dewan Md Emdadul Hoque

**Affiliations:** Maternal and Child Health Division, International Centre for Diarrhoeal Disease Research, Bangladesh (icddr,b), Dhaka, Bangladesh; Univesity of Iowa, UNITED STATES

## Abstract

**Objectives:**

Neonatal deaths account for 45% of all under-five deaths globally and 60% in Bangladesh. This study aimed to investigate the most common symptoms and complications in neonates, care-seeking practices of the mothers for their sick neonates, and factors associated with the care-seeking practices.

**Methods:**

This cross-sectional study analysed data from an Endline Household Survey (as part of an evaluation of a paired cluster-randomised controlled trial study in 14 rural sub-districts in Bangladesh) of 2,931 women who gave birth recently. Descriptive analysis and logistic regressions were conducted to identify the care-seeking practices of mothers of sick neonates and the factors associated with the care-seeking from trained providers.

**Results:**

Of the 2868 neonates, 886 (30.9%) were reported ill during first 28 days after birth. For those with reported symptoms, 748 (84.4%) of their mothers sought care. Of those who sought care, 65.2% sought care from untrained providers. Multiple logistic regression analysis showed significantly higher odds of care-seeking from trained providers when neonates had 3 or more concurrent symptoms (OR: 1.82; 95% CI: 1.07–3.08); when mothers perceived their neonates’ symptoms as severe (OR: 4.08; 95% CI: 2.92–5.70); when mothers received skilled care during pregnancy (OR: 1.95; 95% CI: 1.34–2.84); and when mothers had their delivery in a facility (OR: 3.50; 95% CI: 2.18–5.62). Mothers who delivered their babies at a facility, 43.1% of them sought care for their sick neonates at the same type of public hospital and 34.9% from same type of private hospitals where their deliveries took place.

**Conclusion:**

Skilled care for mothers during pregnancy and delivery, and mothers’ perceptions of the severity of symptoms are the key associated factors of care-seeking for sick neonates from trained providers. Interventions should be tailored to increase care from trained providers during pregnancy and delivery at facilities to improve care-seeking for neonates from trained providers and for the survival of neonates.

## Introduction

Under-five mortality rates have halved globally since the Millennium Development Goal (MDG) baseline in 1990. However, the pace of this improvement varies between age groups within this age classification. The slowest improvement has been observed among neonates (infants below 1 month of age) [1]. More than 90% of neonates die in low and middle income countries (LMICs) [2]; six times higher than neonates die in high-income countries [3]. Neonatal mortality also varies greatly between regions [1] and in-country geographical locations [4, 5]. Although Bangladesh has achieved MDG 4 (reducing under-five mortality to 46 per 1000 live births by 2015), neonatal mortality remains high and is increasingly concentrated especially in the earliest days of neonatal period. Currently, neonatal deaths contribute to more than 60% of all under-five deaths in Bangladesh [4].

Bangladesh Demographic and Health Survey (BDHS) 2011 reported that prematurity/low birth weight (11%), birth asphyxia (21%), and possible serious infection including pneumonia (37%) as the three main causes of neonatal deaths in Bangladesh [6]. Prematurity and intrapartum complications accounted for most early neonatal deaths and infections cause around half of late neonatal deaths which has policy implications for designing effective interventions to address the differentials in the causes of neonatal deaths between the early and late periods [7].

The literature suggests that there are effective interventions that can be implemented around the time of birth for improving neonatal survival in LMICs [8–10]. In the case of sick neonates, health deliveries through an integrated community-facility service can result in improvements in survival. The first steps for providing successful neonatal health care interventions include ensuring the caregivers’ timely recognition of illness and their desire to seek care [2]. Despite the existence of effective interventions– (i.e. domiciliary services, women receiving education for birth preparedness, healthy newborn practices and early care-seeking upon recognizing newborn danger signs) [2, 8–11] care-seeking for sick neonates still remains low in LMICs and is a key challenge in improving neonatal survival [2].

Once a neonate becomes ill, the health condition may rapidly deteriorates. This can result in death if their illness is not identified and treated correctly on time [12, 13]. Healthcare seeking is a complex behaviour, with multiple factors influencing the care-seeking behaviour of a caregiver. These factors include the caregiver’s perception of illness, socio-cultural context, geographical location, quality of care at the available facilities, cost, and maternal and newborn demographic factors [2, 14–17].

Understanding care-seeking behaviours for sick neonates helps in designing effective public health interventions both at the community as well as at the healthcare facility level [17]. Different studies show that care-seeking patterns vary among mothers of neonates, infants and under-five children [2, 18]. However, there is a dearth of community-based data on care-seeking practices for sick neonates, especially in hard to reach areas. The available data shows that the majority of studies targeted children under five years [2]. Therefore, there is a need to explore and understand patterns of and influences on care-seeking behaviour of mothers of sick neonates.

In this study, we focused on identifying the most common symptoms and complications in neonates, exploring the care-seeking practices of mothers of sick neonates and identifying the factors associated with mothers’ care-seeking from trained providers.

## Materials and methods

### Study design and setting

In this study, data was analysed from the Endline Household Survey of a paired cluster-randomised controlled trial (cRCT) which was conducted in 14-sub-districts of five districts (Bandarban, Gopalganj, Kishorganj, Netrokona and Sunamganj), predominantly hard-to-reach areas in Bangladesh. The sub-districts cover an area of about 4640 square kilometer and the population of around 2.5 million. The districts are characterized by its distinct terrain types. Kishoreganj (4 sub-districts), Netrokona (4 sub-districts), and Sumanganj (2 sub-districts) districts are haor areas (haor is bowl-shaped shallow depression which turns into large inland waterbody during monsoon), whereas Bandarban (2 sub-districts) is hilly and Gopalganj (2 sub-districts) is riverine plain land with some chars (char is low-lying area accreted from river/sea). Hoar areas are remote and difficult in terms of poor communication as it is flooded during monsoon. Hilly areas are rugged terrain with forests, lakes and falls, which give it a different character from other parts of the country. Most indigenous people in Bangladesh live in hilly areas. People living in char are vulnerable due to river erosion and flooding. Around 25% of areas in Bangladesh are considered to be hard-to-reach. Details of the interventions, their evaluation designs, and the study settings are available elsewhere [19]. In summary, the objective of the cRCT was to evaluate an integrated package of Maternal, Neonatal and Child Survival (MNCS) interventions that was implemented by the Ministry of Health and Family Welfare, Government of Bangladesh and UNICEF in partnership with other NGOs. The package of integrated interventions included -1) the EPI-plus package that promoted immunization, de-worming and distributing vitamin A supplementation; 2) the IMCI plus package that focused on the prevention and treatment of newborn and child illnesses by families, communities and health care facilities as well as appropriate feeding practices; and 3) the ANC plus package that promoted a minimum three antenatal care (ANC) visits, at least one postnatal care (PNC) visits from a trained healthcare provider, and referrals to existing government facilities. Home visits and community case managers were also provided by the community based government/non-government health workers. Cross-sectional surveys were conducted in all 14 sub-districts at baseline in 2009, and then again via the Endline Survey in 2012 to evaluate the success of the intervention.

### Sample size and sampling

**Fig 1:**
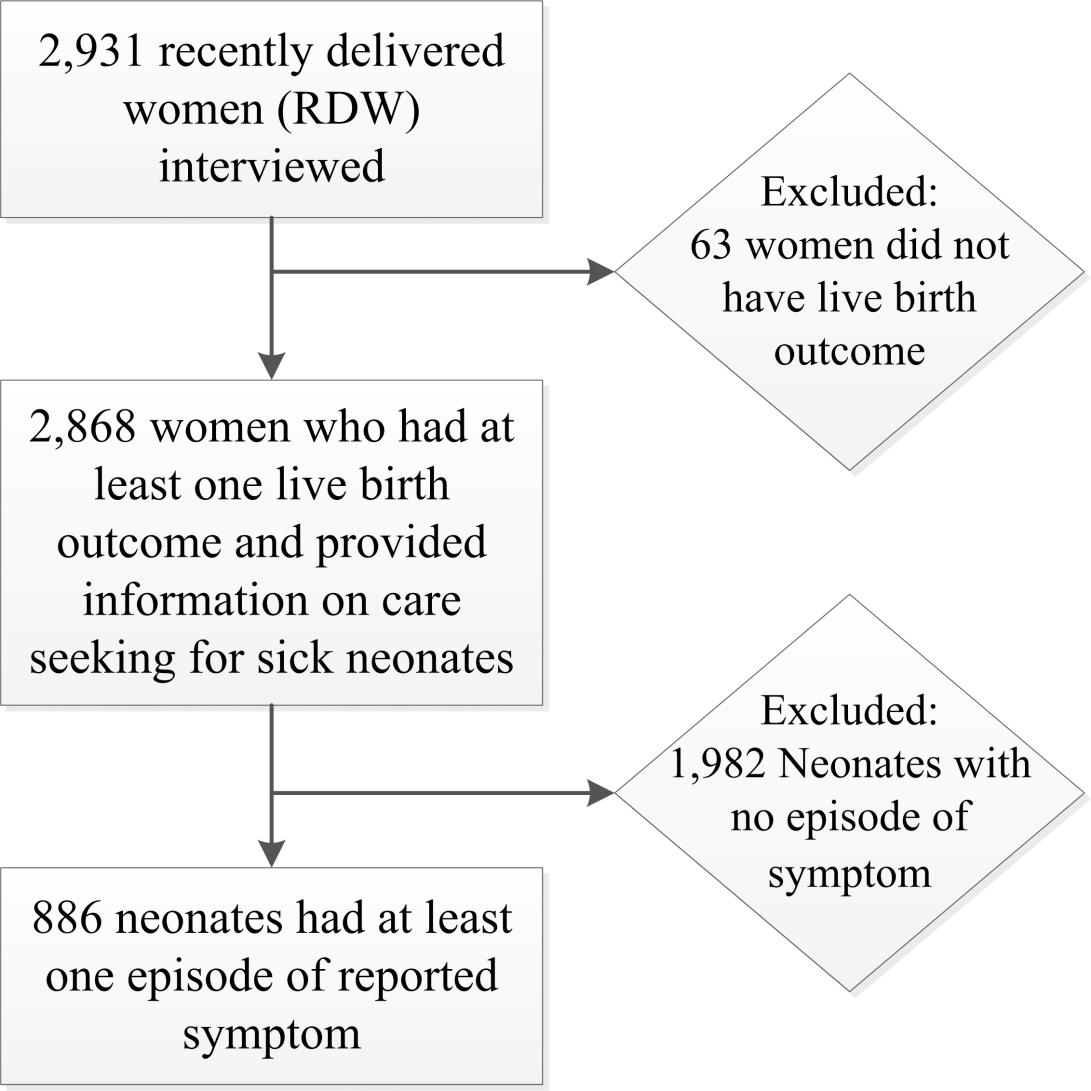
Selection of sample for analysis.

A multi-stage sampling process was adopted to recruit samples from the sub-districts for the intervention and comparison groups. A total of 420 village clusters were selected from 14 sub-districts (30 villages from each) by probability proportion size samples. Thirty-one households were selected randomly from each village cluster. Women having a pregnancy outcome on or after 1 July, 2011 preceding the survey were identified as ‘Recently Delivered Women (RDW)’. Structured interviews were conducted with these 2,931 women who had a recent birth (Fig 1).

### Data collection and management

RDWs who gave consent to participate (98% of total sample) in the study were interviewed using a structured questionnaire. The interview questionnaire included separate sections to collect information on socio-demographic characteristics of the households, women’s background characteristics, and care-seeking behaviours for sick neonates. Locally recruited and trained female data collectors conducted the interviews. Data collection was carried out from September to December 2012. A quality control team comprised of interviewers, field-based supervisors and study investigators who monitored and ensured data integrity by checking the consistency and completeness of forms through spot checks.

### Data processing and statistical analysis

Data collected through the questionnaires were coded and entered into STATA, version 13.0 SE by trained professionals. Descriptive statistical analysis was conducted to explore the distribution of reported symptoms during the neonatal period and mothers’ care-seeking practices. To identify the associated factors of care-seeking from trained provider, univariate and multiple logistic regression were done, where care-seeking from a trained provider was the outcome variable. The potential explanatory variables were identified through reviewing existing literature and based on available data. This study included explanatory variables related to socio-demographic characteristics, perception about the illness, and exposure to facility-based care and/or care from skilled provider during pregnancy and delivery. Univariate analysis was done to see the level of association between each of the explanatory variables and outcome variable. The factors that were found significantly associated with care seeking from trained provider in univariate analysis were considered for building the adjusted model. Odds ratios (with 95% confidence intervals) were used to report the magnitude of associations involved. All data analysis was conducted using STATA, version 13.0 SE.

### Ethical considerations

The MNCS evaluation received approval from the Ethical Review Committee of icddr,b (Protocol number 2007–059). Written informed consent was obtained from the study participants prior to conducting the interviews. The data collectors explained all study participants about the purpose of the study, participants’ right to refuse to answer any question or withdraw from the study at any time. The potential risks and benefits of the study were also explained to the study participants. Appropriate measures were taken to ensure confidentiality during data collection and anonymity during data management, analysis, and reporting.

## Results

Of 2,868 mothers, 48.2% were aged between 25–34 years, and 41.6% were under 25 years of age. 38.4% mothers had no education, and only 13.6% mothers had deliveries in a facility. Participant background characteristics are presented in more detail in Table 1.

**Table 1:** Background characteristics of study participants, MNCS Endline Survey in 2012.

Background characteristics	Overall (N = 2868)	Healthy neonates (N = 1982)	Sick neonates (N = 886)
	n (%)	n (%)	n (%)
**Maternal age (in years) at interview**			
15–24	1194 (41.6)	814 (41.1)	380 (42.9)
25–34	1382 (48.2)	964 (48.6)	418 (47.2)
35+	292 (10.2)	204 (10.3)	88 (9.9)
**Maternal education**			
No formal education	1105 (38.5)	767 (38.7)	338 (38.2)
Primary complete	1017 (35.5)	671 (33.9)	346 (39.1)
Secondary incomplete	617 (21.5)	443 (22.4)	174 (19.6)
Secondary and above	128 (4.5)	101 (5.1)	27 (3.1)
**Father’s education**			
No formal education	1326 (46.2)	895 (45.2)	431 (48.7)
Primary complete	868 (30.3)	585 (29.5)	283 (31.9)
Secondary incomplete	474 (16.5)	346 (17.5)	128 (14.5)
Secondary and above	192 (6.7)	151 (7.6)	41 (4.6)
**Birth order**			
1	824 (28.7)	564 (28.5)	260 (29.4)
2	732 (25.5)	521 (26.3)	211 (23.8)
3	544 (19.0)	371 (18.7)	173 (19.5)
4 or more	768 (26.8)	526 (26.5)	242 (27.3)
**Place of delivery**			
Home	2476 (86.3)	1701 (85.8)	775 (87.5)
Facility	389 (13.6)	278 (14.0)	111 (12.5)
Other	3 (0.1)	3 (0.2)	-
**Wealth quintile**			
Lowest	551 (19.2)	352 (17.8)	199 (22.5)
Second	589 (20.5)	379 (19.1)	210 (23.7)
Middle	589 (20.5)	393 (19.8)	196 (22.1)
Forth	568 (19.8)	442 (22.3)	126 (14.2)
Highest	571 (19.9)	416 (21.0)	155 (17.5)

30.9% of neonates (886 of 2868) were reported ill during the survey, of which 36.7% (325 of 886) were reported to have more than one symptom. Two thirds of the sick neonates were reported to have suffered from fever (raised temperature). After fever, the most commonly reported symptoms were difficult or fast breathing (28.1%), low temperature (27.8%), poor sucking or feeding (13.4%), and chest in-drawing (10.4%). Of those who reported symptoms, 748 (84.4%) of them sought care, with around 65% of these sick neonates seeking care from untrained providers. Only 29.4% of sick neonates received care from medically trained providers, who were mostly medical doctors 93.8% (244 of 260). Mothers of sick neonates primarily sought care from private health facilities (57.0%), with the next most common choice being government health facilities (19.1%). The common reasons for not seeking care were reported to be financial problem (52.2%) and mothers did not think care-seeking was necessary (44.9%) (Table 2).

**Table 2:** Reported symptoms and mother’s preferences of care-seeking for their sick neonates, MNCS Endline Survey in 2012.

Characteristics			n (%)
**Symptom category [N = 886]**			
	Any symptom		886 (30.9)
	1 symptom		561 (63.3)
	2 symptoms		239 (27.0)
	3 or more symptoms		86 (9.7)
**Reported neonatal symptom** ^a^ **[N = 886]**			
	Baby feels hot/Fever		575 (64.9)
	Difficult or fast breathing		249 (28.1)
	Baby feels cold		246 (27.8)
	Chest in-drawing		92 (10.4)
	Poor sucking or feeding		119 (13.4)
	Convulsions/Spasms/Rigidity		36 (4.1)
	Lethargy/Unconsciousness		31 (3.5)
**Sought care [N = 884]**			
	Yes		748 (84.4)
	No		136 (15.4)
**Types of providers [N = 884]**			
	Trained provider		260 (29.4)
		Medical doctor	244 (27.6)
		Nurse/Midwife/ Paramedic/SACMO/FWV	16 (1.8)
	Untrained provider		488 (55.2)
		Village doctor	305 (34.5)
		Homeopathic practitioner	91 (10.3)
		Allopathic drug stores	50 (5.7)
		Others	42 (4.7)
	Did not seek care		136 (15.4)
**Places of care-seeking [N = 884]**			
	Home		72 (8.1)
	Govt. health centers		169 (19.1)
		Sub-district level hospital	104 (11.8)
		District level hospital	33 (3.7)
		Medical college hospital	13 (1.5)
		Health facility at union level and below	19 (2.1)
	Private health care facilities		504 (57.0)
	Non-govt. health centers		3 (0.3)
	Did not seek care		136 (15.4)
**Reason for not seeking care** ^a^ **[N = 136]**			
	Financial problem		71 (52.2)
	Did not think care seeking is necessary		61 (44.9)
	Far distance of health facility from home		34 (25.0)
	Transport problem		31 (22.8)
	Others		20 (14.7)

^a^ Multiple responses allowed.

Percentages of care-seeking from trained providers was higher when neonates had suffered from three or more symptoms (47.7%). They were also higher among mothers aged below 25 years (36.0%), mothers who had a secondary or above education (59.3%), mothers who had received ANC services from a trained provider (44.4%), mothers who were from the richest quintile (42.6%), and when mothers perceived symptom/s as severe (48.9%) (Table 3).

**Table 3:** Factors associated with care-seeking for sick neonates from trained providers (N = 886).

Background factors		Sick neonates n (%)	Care seeking from trained providers n (%)	Unadjusted		Adjusted	
				OR (95% CI)	P-value	OR (95% CI)	P-value
**Perceived severity of symptom**							
	Severe	323 (36.5)	158 (48.9)	4.32 (3.19–5.88)	<0.001	4.08 (2.92–5.70)	<0.001
	Not severe	563 (63.5)	102 (18.2)	*Reference group*			
**Symptom category**							
	3 or more symptoms	86 (9.7)	41 (47.7)	2.69 (1.69–4.28)	<0.001	1.82 (1.07–3.08)	0.026
	2 symptoms	239 (27.0)	77 (32.4)	1.40 (1.01–1.95)	0.045	1.14 (0.78–1.66)	0.504
	1 symptom	561 (63.3)	142 (25.4)	*Reference group*			
**Maternal education**							
	Secondary and above	27 (3.1)	16 (59.3)	5.21 (2.32–11.7)	<0.001	2.67 (1.03–6.88)	0.043
	Secondary incomplete	174 (19.6)	73 (42.2)	2.59 (1.74–3.85)	<0.001	1.52 (0.95–2.45)	0.081
	Primary complete	346 (39.1)	97 (28.1)	1.40 (0.98–1.98)	0.061	1.30 (0.89–1.91)	0.176
	No formal education	338 (38.2)	73 (21.6)	*Reference group*			
**Maternal age**							
	15–24	380 (42.9)	136 (36.0)	1.33 (0.80–2.20)	0.268		
	25–34	418 (47.2)	98 (23.4)	0.73 (0.44–1.22)	0.228		
	35+	88 (9.9)	26 (29.6)	*Reference group*			
**Antenatal care**							
	Trained provider	297 (33.7)	132 (44.4)	2.83 (2.05–3.90)	<0.001	1.95 (1.34–2.84)	0.001
	Untrained provider	145 (16.4)	29 (20.1)	0.88 (0.56–1.41)	0.603	0.70 (0.43–1.16)	0.164
	No ANC received	440 (49.9)	97 (22.1)	*Reference group*			
**Delivery places**							
	Facility	111 (12.5)	68 (61.3)	4.80 (3.17–7.27)	<0.001	3.50 (2.18–5.62)	<0.001
	Home	775 (87.5)	192 (24.8)	*Reference group*			
**Birth order**							
	1	260 (29.4)	97 (37.5)	2.23 (1.50–3.32)	<0.001		
	2	211 (23.8)	67 (31.9)	1.74 (1.14–2.66)	0.010		
	3	173 (19.5)	45 (26.0)	1.32 (0.83–2.08)	0.240		
	4 or more	242 (27.3)	51 (21.1)	*Reference group*			
**Delivery attended**							
	Skilled	132 (14.9)	66 (50.0)	2.89 (1.98–4.21)	<0.001		
	Unskilled	754 (85.1)	194 (25.8)				
**Household wealth**							
	1st quintile (Lowest)	199 (22.5)	43 (21.6)	*Reference group*			
	2nd quintile	210 (23.7)	51 (24.3)	1.16 (0.73–1.85)	0.520		
	3rd quintile	196 (22.1)	59 (30.3)	1.56 (0.99–2.46)	0.055		
	4th quintile	126 (14.2)	41 (32.8)	1.75 (1.06–2.89)	0.029		
	5th quintile (Highest)	155 (17.5)	66 (42.6)	2.69 (1.69–4.28)	<0.001		

The unadjusted univariate logistic regression analysis provided the list of significant variables that were included in the adjusted model. In the multiple logistic regression model, after adjusting for other variables, the odds of mothers who perceived the symptom/s to be severe seeking care from trained providers remained high (OR: 4.08; 95% CI: 2.92–5.70). Similarly, care-seeking from trained providers was 1.82 times higher (OR: 1.82; 95% CI: 1.07–3.08) among neonates with three or more symptoms. Mothers who received antenatal care from trained providers had a higher tendency of care-seeking from trained providers for their sick neonates (OR: 1.95; 95% CI: 1.34–2.84). Mothers who delivered at healthcare facilities were 3.50 times (OR: 3.50; 95% CI: 2.18–5.62) more likely to seek care from trained providers (Table 3).

Additionally, in the sub-group analysis we found that the proportion of mothers who delivered their babies at a facility were likely to seek care from the same type of facility when their neonates were sick; 43.1% for public hospital and 34.9% for private hospital/clinic. The proportions of care-seeking from the same type of facility did not differ between primary and secondary/tertiary level public hospitals (Table 4).

**Table 4:** Proportion of care-seeking for sick neonates from the same facility where delivery was conducted.

	Facility n (%)	Home n (%)
Delivery	389 (13.6)	2476 (86.4)
Sick neonates	111 (28.5)	775 (31.3)
Care-seeking from trained provider	68 (61.3)	192 (24.8)
*Care-seeking from same type of facility*		
Public hospital	25 (43.1)	-
Secondary/tertiary level hospital	10 (43.5)	-
Primary level facility hospital	15 (42.9)	-
NGO	0 (0.0)	-
Private hospital/clinic	15 (34.9)	-

## Discussion

The objective of this study was to explore the distribution of reported neonatal symptoms and pattern of mothers’ care-seeking practices, and to identify the factors associated with care-seeking from trained providers.

### Reported symptom and mother’s preferences of care-seeking for their sick neonates

This study found a high prevalence (one of every three neonates) of symptoms during the first 28 days after birth, though it was lower when compared with findings from previous studies [5, 20]. Fever was found to be the most common symptom followed by difficult or fast breathing [5, 21]. These two symptoms are associated with Acute Respiratory Illness (ARI); a common childhood illness in Bangladesh. One-third of the sick neonates in this study were reported to have multiple concurrent symptoms which is similar to the findings reported in a previous study on under five children in Bangladesh [22].

Consistent with other studies within the Bangladesh context, the majority (four out of five) of the mothers in this study reported of receiving care for their sick neonates either at home or at facilities [5, 22, 23]. However, more than half of these neonates received care from untrained providers including village doctors, non-allopathic practitioners, traditional and spiritual healers and pharmacists at local drug stores [5, 17, 21, 24]. Village doctors remained the most frequent choice among untrained providers. Village doctors are considered to be easily accessible, relatively cheap, and more culturally acceptable [17, 21, 24]. Most of the mothers who sought care from trained providers went to medical doctors; particularly at upazila (sub-district) level hospitals known as Upazila Health Complexes (UHC) [24]. UHC is a primary health care referral facility with out-patient, emergency and in-patient services where medical doctors are the main service providers. Allopathic medicine was found to be the most preferred method of treatment followed by homeopathic medicine [15, 17]. The popularity of allopathic medicine may be attributable to the relatively faster working properties and widespread availability in the local drugstores. Mothers may have been attracted to homeopathic medicine to treat their sick neonates due to the low cost and the belief that it is milder and causes no side effects [5].

### Factors associated with care-seeking for sick neonates from trained providers

This study also explored the factors associated with mother’s care-seeking for reported symptoms from trained providers. Mothers perceptions of the severity of symptoms, previous exposure to antenatal care from trained providers and delivery at a facility were identified as the key associated factors of care-seeking from trained providers. The presence of multiple symptoms and the mother’s education were also significantly related with increased care-seeking from trained providers.

A review of care-seeking practices for newborn sickness in South-Asia identified delays in recognizing neonatal danger signs in the community setting. Once danger signs were recognized, caregivers tended to seek care from untrained providers initially [15]. However, when multiple complications were present, caregivers were more likely to seek care from trained providers [21, 24]. Mothers who had received antenatal care from a trained provider were about two times more likely to seek care from trained providers for their sick neonates. Similarly, mothers who had given birth at facilities were 3.5 times likely to seek care from trained providers compared to those who had not delivered at the facilities. The reason for these differences may be that previous exposure to maternal health care services from trained provider increases awareness regarding the importance and availability of these services, and strengthens mother’s trust in these providers leading to positive care-seeking practices for subsequent neonatal sickness [5, 25]. In addition to this, as the majority of neonatal sickness occurs in the first week after birth, it may be that mothers delivering in healthcare facilities are more likely to receive the services directly if their neonates get sick during their stay. Our sub-group analysis among the mothers who had given birth in facilities identified that more than 40 percent of these mothers sought care for their sick neonates from the same type of facilities where the deliveries took place. In addition, the availability of newborn care services also influences care-seeking for sick neonates.

Educated mothers (with secondary and above education) were more than 2.5 times more likely to seek care from trained providers compared to mothers with no education. This strengthens the argument that educated mothers are more likely to have better knowledge of health services and better decision-making abilities that may lead them to seek care from trained providers [23].

### Study strengths and limitations

This study was carried out in hard-to-reach areas with a relatively large number of participants. However, data used in this study were from interviews with an 18 months recall period and therefore, were susceptible to recall errors when reporting symptoms and care-seeking practices. To address this issue, data collectors were trained rigorously on appropriate questioning methods and potential healthcare sources to avoid misclassifications. Information on the timing and duration of the symptoms after delivery, and promptness of recognition of symptoms and care seeking were not collected during the survey which restricted us from analyzing and explaining if the observed predictors for care-seeking from trained providers were different in different groups. We also did not have data on availability of different types of service providers in the study sub-districts. However, disaggregated analysis showed similar distribution of care seeking from different providers across the sub-districts.

## Conclusions

Raised temperature and breathing difficulty remain the most common symptoms among neonates. Although care-seeking practices are high for sick neonates, most of them receive care from untrained providers, particularly from the village doctor. However, medical doctors are the most commonly chosen service provider when care is sought from trained providers. Mother’s perceptions of the severity of symptoms, whether they received antenatal care from trained providers during pregnancy and management during delivery at a facility or not are the key associated factors of care-seeking from trained providers. Interventions should be designed to increase the coverage of care from trained providers during pregnancy and facility delivery to improve care-seeking for sick neonates from trained providers, and ultimately the survival of neonates. Adequate and appropriate health education programmes at the family and community levels to ensure skilled maternal care and early recognition of newborn danger signs should be intensified. Further studies are needed to understand the specific predictors of care-seeking from trained providers and its trends at the community and facility levels.

## Supporting information

S1 File Women and under 5 children questionnaire for MNCS/C-IMCI endline household survey 2012. (PDF)
