# Supplementary material for: Care-seeking practices for sick neonates: Findings from cross-sectional survey in 14 rural sub-districts of Bangladesh
Source: PLoS One. 2018 Sep 27;13(9):e0204902. doi: 10.1371/journal.pone.0204902 (PMC6160193; doi:10.1371/journal.pone.0204902)
Supplement: S1 File — (PDF) [file pone.0204902.s001.pdf]

MNCS / C-IMCI Endline Household Survey 2012

Module II - V

Women and their under 5 children

	Name	Code
DISTRICT		☐
UPAZILA		☐☐
UNION		☐☐☐
VILLAGE		☐☐-☐☐☐☐
BARI		☐☐☐
HOUSEHOLD HEAD		☐☐☐☐
WOMAN		Line no |___|___| ☐ of ☐
HUSBAND		
ADDRESS		

সাক্ষাৎকার শুরু হবার সময়:

|___|___|:|___|___|

সাক্ষাৎকার শেষ করার সময়

|___|___|:|___|___|

Interviewer's Visits and Status

	Visit 1	Visit 2	Visit 3
Date	|__|__|-|__|__|-|__|__|	|__|__|-|__|__|-|__|__|	|__|__|-|__|__|-|__|__|
Interviewer's Name			
Result code*	|__|__|	|__|__|	|__|__|
Next Visit	|__|__|-|__|__|-|__|__|	|__|__|-|__|__|-|__|__|	

Final Visit	
Date	|__|__|-|__|__|-|__|__|
Interviewer's code	|__|__|__|
Result code	|__|__|
Total # of visits	|__|

মহিলার 1 (পহেলা) জুন 2011 সাল বা তার পরে গর্ভ ফলাফল আছে The woman had an outcome since 01-Jun-2011	Yes No
1 (পহেলা) জুন 2007 সাল বা তার পরে জন্ম নেয়া এবং বর্তমানে জীবিত বাচ্চার সংখ্যা Number of Alive Under 5 children (DOB since 01-Jun-2007)	☐
গত দুই সপ্তাহে কখনও অসুস্থ ছিল এমন বাচ্চার সংখ্যা Number of Sick Child (2 wk morbidity) identified in this HH	☐

***Result Codes**

01. Interview Complete
02. Woman married and aged between 15-49 years is absent
03. Refused to give interview (partial refusal)
04. Refused to give interview (full refusal)
05. Woman is never married or aged not between 15-49 years
07. Others____________________

Supervision	Name	Code	Date
Reviewed by FS		|___|___|	|___|___|-|___|___|-|___|___|
Checked by FRS		|___|___|	|___|___|-|___|___|-|___|___|
Reviewed by QC Team		|___|___|	|___|___|-|___|___|-|___|___|
Data Entered by		|___|___|	|___|___|-|___|___|-|___|___|

Module II
Women Background, Reproductive and Birth History

Section C: Background

This Section contains some background information of the respondent (woman) and her husband

আপনি এবং আপনার স্বামী সম্পর্কে এখন আমি আপনাকে কিছু প্রশ্ন করতে চাই

First I would like to ask some questions about you and your husband.

NO.	QUESTIONS AND FILTERS	CODING CATEGORIES	SKIP
C01	আপনি কোন সালের কোন মাসে জন্ম গ্রহন করেছিলেন?<br>In what month and year were you born?	মাস (Month) ....|____|____| জানি না (Don't know month)..99<br>সাল (Year)................................... |____|____||____|____|	
C02	বর্তমানে আপনার বয়স কত?<br>(C01 ও C02 মিলিয়ে দেখুন, অসামঞ্জস্য হলে C01 ও C02 সংশোধন করুন)How old were you at your last birthday?	বয়স (পূর্ন বছরে) Age in completed years ....|____|____|	
C03	আপনার যখন বিয়ে হয়েছিল, তখন আপনার বয়স কত ছিল?<br>How old were you when you got married?	বয়স (পূর্ন বছরে) Age in completed years ....|____|____|	
C04	আপনি কি কখনও স্কুলে, মাদ্রাসায় বা উপানুষ্ঠানিক শিক্ষা স্কুলে (বয়স্ক শিক্ষা কেন্দ্র বা অন্যকোথাও) লেখাপড়া করেছেন?<br>Did you ever study in school, madrasa or non formal school?	হ্যাঁ (Yes) .... 1<br>না (No) 2	 →C07
C05	আপনি সর্বোচ্চ কোন ক্লাস/শ্রেণী পর্যন্ত লেখাপড়া করেছেন?<br>(কোন ক্লাস/শ্রেণী সম্পুর্ন করে না থাকলে "00" লিখুন)<br>What is the highest grade/class or number of years of studies you have completed at that schooling?<br>*[Write '00' if no class completed]*	সর্বোচ্চ কোন ক্লাস/শ্রেণী Highest Class/Grade..........................|____|____|	
C06	আপনি কোথায় কোথায় লেখাপড়া করেছেন?<br>What type of institution/s have you attended?	স্কুল School/College/University .... A<br>বোর্ড" মাদ্রাসা "Board" madrasah.. B<br>কওমী মাদ্রাসা "Qowmi" madrasah... C<br>উপানুষ্ঠানিক শিক্ষা স্কুল Non Formal Education Program .... D<br>অন্যান্য Others ________________________________.... X	
C07	আপনি কি দৈনিক খবরের কাগজ বা অন্যান্য পত্রিকা/ম্যাগাজিন পড়েন? Do you read a newspaper or magazine?	হ্যাঁ Yes .... 1<br>না No 2	 →C09
C08	আপনি কত ঘন ঘন দৈনিক খবরের কাগজ বা অন্যান্য পত্রিকা/ম্যাগাজিন পড়েন? প্রতিদিন পড়েন, নাকি সপ্তাহে কমপক্ষে একদিন পড়েন, নাকি আরও কম পড়েন?<br>How often do you read newspaper or magazine?	প্রতিদিন Almost everyday ....1<br>অন্তত সপ্তাহে একদিন At least once a week.....................................2<br>খুবই কম সপ্তাহে ১ দিনও না Less than once a week 3	
C09	আপনি কি রেডিও শোনেন?<br>Do you listen to radio?	হ্যাঁ Yes .... 1<br>না No 2	 →C11
C10	আপনি কত ঘন ঘন রেডিও শোনেন? প্রতিদিন শোনেন, নাকি সপ্তাহে কমপক্ষে একদিন শোনেন, নাকি আরও কম শোনেন?<br>How often do you listen to radio?	প্রতিদিন Almost everyday ....1<br>অন্তত সপ্তাহে একদিন At least once a week.....................................2<br>খুবই কম সপ্তাহে ১ দিনও না Less than once a week 3	
C11	আপনি কি টেলিভিশন দেখেন?<br>Do you watch television?	হ্যাঁ Yes .... 1<br>না No 2	 →C13
C12	আপনি কত ঘন ঘন টেলিভিশন দেখেন? প্রতিদিন দেখেন, নাকি সপ্তাহে কমপক্ষে একদিন দেখেন, নাকি তারও কম দেখেন?<br>How often do you watch television?	প্রতিদিন Almost everyday ....1<br>অন্তত সপ্তাহে একদিন At least once a week.....................................2<br>খুবই কম সপ্তাহে ১ দিনও না Less than once a week 3	
C13	আপনার TV তে কি কেবল / ডিশের সংযোগ আছে?<br>Does the television you watch has a cable connection?	হ্যাঁ Yes .... 1<br>না No .... 2<br>প্রযোজ্য নয় Not Applicable 9	

NO.	QUESTIONS AND FILTERS	CODING CATEGORIES	SKIP
C14	আপনার ধর্ম কি?<br>What is your religion?	ইসলাম Islam ....1<br>হিন্দু Hinduism ....2<br>বৌদ্ধ Buddhism ....3<br>খ্রীস্টান Christianity ....4<br>অন্যান্য Others________ 7	
C15	ঘরের কাজের পাশাপাশি আপনি এমন কোন কাজ কি করেন যা থেকে আপনার আয় হয়? যেমন, কেউ জিনিসপত্র বিক্রী করেন, কেউ নিজের ছোট ব্যবসায় বা পারিবারিক খামারে বা ব্যবসায় কাজ করেন, কেউ গরু-ছাগল বর্গা নেন ইত্যাদি।<br>As you know, some women take up jobs for which they are paid in cash or kind. Others sell things, have a small business or work on the family farm or in the family business.<br>Are you doing any of this things or any work?	হ্যাঁ Yes .... 1<br>না No 2	 →C18
C16	প্রধানতঃ আপনি কি কাজ করেন?<br>*[একাধিক পেশার সাথে জড়িত হলে প্রধান পেশার নাম নীচে লিখে ডান দিকের কোড বৃত্তায়িত করুন।]*<br>পেশাঃ (Occupation)________<br>What is your primary occupation, that is, what kind of work do (did) you mainly do?	**কায়িক পরিশ্রম Physical work**<br>অদক্ষ (যেমন কামলা, মাটি কাটা) Unskilled laborer ....11<br>দক্ষ (যেমন কাঠের কাজ, মিস্ত্রি, সিমেন্টের কাজ, রিক্সা চালক) Skilled worker ....12<br>**বিনা কায়িক পরিশ্রম Non physical work**<br>ব্যবসা Business/trade ....21<br>চাকুরী Service holder ....22<br>পেশাজীবি (ডাক্তার/ ইনজিনিয়ার/শিক্ষক) Professional ....23<br>**অন্যান্য** Other ________ ....97<br>নির্দিষ্ট করুন	
C17	*[উল্লেখিত প্রধান পেশা কি কৃষিকাজ বা মাছ চাষাবাদের সাথে জড়িত]* *[Is the main occupation of the respondent involved agriculture, farming or fishing?]*	হ্যাঁ (Yes) .... 1<br>না (No) 2	
C18	আপনি বর্তমানে বিবাহিতা, বিচ্ছিন্না, পরিত্যক্তা, বিধবা না তালাকপ্রাপ্তা?<br>Are you now married, separated, deserted, widowed, or divorced?	বর্তমানে বিবাহিত Currently Married ....1<br>বিচ্ছিন্না Separated ....2<br>পরিত্যক্তা Deserted ....3<br>তালাকপ্রাপ্তা Divorced .... 4<br>বিধবা Widowed 5	 →D01<br>→D01<br>→D01<br>→D01
C19	বর্তমানে আপনার স্বামীর বয়স কত?<br>How old was your husband at his last birthday?	বয়স (পূর্ন বছরে) Age in completed years .... |____|____| জানি না Don't know 99	
C20	আপনার স্বামী কি কখনও স্কুলে, মাদ্রাসায় বা উপানুষ্ঠানিক শিক্ষা স্কুলে (বয়স্ক শিক্ষা কেন্দ্র বা অন্যকোথাও) লেখাপড়া করেছেন?<br>Did your husband ever study in school, madrasa or adult literacy school?	হ্যাঁ Yes .... 1<br>না No .... 2<br>জানি না/ মনে নাই Don't know/Can't remember 9	 →C23<br>→C23
C21	আপনার স্বামী সর্বোচ্চ কোন ক্লাস/শ্রেণী পর্যন্ড় লেখাপড়া করেছেন? *[কোন ক্লাস/শ্রেণী সম্পুর্ন করে না থাকলে "00" লিখুন যদি]*What is the highest grade/class or number of years of studies your husband have completed at that schooling?(Write '00' if no class completed)	সর্বোচ্চ কোন ক্লাস/শ্রেণী Highest Class/Grade.... |____|____| জানি না/ মনে নাই Don't know/Can't remember 99	
C22	আপনার স্বামী কোথায় কোথায় লেখাপড়া করেছেন?<br>What type of institution/s have your husband attended?	স্কুল School/College/University .... A<br>বোর্ড মাদ্রাসা "Board" madrasah .... B<br>কওমী মাদ্রাসা "Qowmi" madrasah.... C<br>উপানুষ্ঠানিক শিক্ষা স্কুল Non Formal Education Program .... D<br>অন্যান্য Others ________ .... X<br>জানি না/ মনে নাই Don't know/Can't remember .... Z	
C23	বর্তমানে আপনার স্বামী আয় রোজগারের জন্য কোন কাজ করেন কি? Does your husband do anything for living?	হ্যাঁ Yes .... 1<br>না No 2	 →D01

NO.	QUESTIONS AND FILTERS	CODING CATEGORIES	SKIP
C24	প্রধানতঃ আপনার স্বামী কি কাজ করেন?<br>*[একাধিক পেশার সাথে জড়িত হলে প্রধান পেশার নাম নিচে লিখে ডান দিকের কোড বৃত্তায়িত করুন ।]*<br>পেশাঃ (Occupation)________________<br><br>What is his primary occupation?	**কায়িক পরিশ্রম Physical work**<br>অদক্ষ (যেমন কামলা, মাটি কাটা) Unskilled laborer ....11<br>দক্ষ (যেমন কাঠের কাজ, মিস্ত্রি, সিমেন্টের কাজ, রিক্সা চালক) Skilled worker ....12<br>**বিনা কায়িক পরিশ্রম Non physical work**<br>ব্যবসা Business/trade ....21<br>চাকুরী Service holder..22<br>পেশাজীবি (ডাক্তার/ ইনজিনিয়ার/শিক্ষক) Professional ....23<br>**অন্যান্য** Other ________________________.........97<br>নির্দিষ্ট করুন	
C25	*[উল্লেখিত প্রধান পেশা কি কৃষিকাজ বা মাছ চাষাবাদের সাথে জড়িত] [Is the main occupation of the respondent's husband involve agriculture, farming or fishing]*	হ্যাঁ (Yes) .... 1<br>না (No) 2	

Section D: Reproduction and Birth History

This Section contains the basic history of all the births the woman had given during her lifetime

আপনার জীবনে আপনি যতবার গর্ভবতী হয়েছিলেন তার সবগুলো সম্পর্কে এখন আমি আপনাকে প্রশ্ন করবো

Now I would like to ask about all the pregnancy you have had during your life.

NO.	QUESTIONS AND FILTERS	CODING CATEGORIES	SKIP
D01	আপনার কি কখনও কোন ছেলে মেয়ে হয়েছে?<br>Have you ever given birth?	হ্যাঁ Yes ....1<br>না No 2	→D06
D02	আপনি জন্ম দিয়েছেন, এমন ছেলে অথবা মেয়ে কি এখন আপনার সাথে বসবাস করছে? Do you have any sons or daughters to whom you have given birth who are now living with you?	হ্যাঁ Yes ....1<br>না No 2	→D04
D03	কয়জন ছেলে আপনার সাথে বসবাস করছে? কয়জন মেয়ে আপনার সাথে বসবাস করছে?<br>*[কোন ছেলে মেয়ে সাথে বসবাস না করলে, '00' লিখুন]*<br>How many sons live with you? And how many daughters live with you?	বাড়ীতে থাকে এমন ছেলের সংখ্যা Sons at home .... |____|____| বাড়ীতে থাকে এমন মেয়ের সংখ্যা Daughters at home .... |____|____|	
D04	আপনি এমন কোন ছেলে বা মেয়ে এর জন্ম দিয়েছেন কি যারা জীবিত আছে কিন্তু আপনার সাথে বসবাস করে না?<br>Do you have any sons or daughters whom you have given birth who are alive but do not live with you?	হ্যাঁ Yes ....1<br>না No 2	→D06
D05	কয়জন জীবিত ছেলে আছে, যারা আপনার সাথে বসবাস করে না? এবং কয়জন জীবিত মেয়ে আছে, যারা আপনার সাথে বসবাস করে না? *[কোন ছেলে মেয়ে সাথে বসবাস না করলে, '00' লিখুন]*<br>How many sons are alive but do not live with you? And how many daughters are alive but do not live with you?	অন্য কোথাও থাকে এমন ছেলে সংখ্যা Sons Elsewhere....... |____|____| অন্য কোথাও থাকে এমন মেয়ের সংখ্যা Daughters elsewhere |____|____|	
D06	আপনি কি কখনও এমন কোন ছেলে বা মেয়ে জন্ম দিয়েছেন, যে জীবিত জন্ম নিয়েছিলো কিন্তু পরে মারা গিয়েছিলো?<br>*[যদি না হয়, যাচাই করুনঃ]* এমন কোন ছেলে বা মেয়ে, যে জন্ম নেয়ার পর কেঁদেছিলো বা যার মধ্যে জীবনের লক্ষণ দেখা গিয়েছিল, কিন্তু কয়েক মিনিট বা কয়েক ঘন্টা বা কয়েক দিন মাত্র জীবিত ছিল অর্থ্যাৎ পরে কোন সময় মারা গিয়েছিলো?<br>Have you ever given birth to a boy or girl who was born alive but later died? (Any baby who cried or showed signs of life but did not survive?)	হ্যাঁ Yes ....1<br>না No 2	→D08
D07	সর্বমোট কয়জন ছেলে মারা গেছে? সর্বমোট কয়জন মেয়ে মারা গেছে? *[ছেলে মেয়ে মারা না গিয়ে থাকলে, '00' লিখুন]*<br>How many boys have died? And how many girls have died?	ছেলে মারা গেছে Boys dead.................................... |____|____| মেয়ে মারা গেছে Girls dead .... |____|____|	
D08	কোন কোন গর্ভাবস্থা পূর্ন মেয়াদের আগেই গর্ভনষ্ট বা গর্ভপাত হিসাবে শেষ হয়ে যেতে পারে। আবার কোন কোন গর্ভাবস্থা মৃতজন্ম বা মৃত শিশুর জন্মও দিতে পারে অর্থাৎ যার জন্মের সময় জীবনের কোন লক্ষন থাকে না।<br>আপনার জীবনে কি কখনও এ ধরনের কোন ঘটনা অর্থাৎ মৃত বাচ্চা জন্ম দেয়া অথবা গর্ভনষ্ট বা গর্ভপাত এর মত ঘটনা ঘটেছিল?<br>Have you had any pregnancies that did not result in live births? Some pregnancies end before full term as miscarriage or an abortion, while others may result in a stillbirth.	হ্যাঁ Yes ....1<br>না No 2	→D10
D09	মোট কতগুলো গর্ভাবস্থার ক্ষেত্রে মৃত বাচ্চা জন্ম দেয়া অথবা গর্ভনষ্ট বা গর্ভপাত এর মত ঘটনা ঘটেছে?<br>In all, how many pregnancies did not result in a live birth?	গর্ভ নষ্টের সংখ্যা (Pregnancy Loss).................................|___|___|	
D10	*[প্রশ্ন D03 , D05, D07 এবং D09 যোগ করুন এবং পাশের বক্সে লিখুন।]*	মোট গর্ভের ফলাফলের সংখ্যা (#of pregnancy outcomes)..........|___|___|	

NO.	QUESTIONS AND FILTERS	CODING CATEGORIES	SKIP
D11	*[প্রশ্ন D10 দেখুন এবং জিজ্ঞাসা করুন: (CHECK D10 and ask)]*<br><br>এখন এই হিসাবটি ঠিকভাবে করেছি কিনা তা নিশ্চিত হবার জন্য আপনাকে আবার জিজ্ঞাসা করছি:<br><br>আপনার ______ জন সন্তান এখন জীবিত (D03+D05)<br>আপনার _______ জন সন্তান মারা গেছে (D07), এবং<br>আপনার _______ টি গর্ভাবস্থার পরে জীবিত সন্তান না হওয়ার মত ঘটনা ঘটেছে (D09)<br><br>আমার এই হিসাব কি ঠিক?<br><br>Just to make sure that I have this is correct: you have had<br>______# children still alive (D03+D05)<br>______# children have died (D07), and<br>______# pregnancies which did not result in a live birth (D09)?<br><br>Is that correct?	হ্যাঁ .... 1<br>না .... 2<br><br>*[উত্তর যদি "না" হয় তবে প্রশ্ন করুন এবং D01 থেকে D10 শুদ্ধ করুন।]*	 →Check D01-D10
D12	*[প্রশ্ন D10 দেখুন এং সঠিক কোড বৃত্তায়িত করুন]*<br>[Check D10 and circle appropriate code]	মোট গর্ভের ফলাফলের সংখ্যা 01 বা তার অধিক (Total pregnancy 01 or more) ....1<br>মোট গর্ভের ফলাফলের সংখ্যা 00 (Total pregnancy 00) 2	 <br>→End

আপনি জীবনে যতবার গর্ভবতী হয়েছেন, এবার আমি তার প্রত্যেকটির ব্যাপারে আলোচনা করতে চাই। সেই গর্ভের বা গর্ভাবস্থাগুলো জীবিত শিশু বা মৃতশিশু বা গর্ভ/গর্ভসমূহ মেয়াদের আগে শেষ হয়ে যাওয়া যাই হোক না কেন সবগুলো সম্পর্কেই আলোচনা করতে চাই। আপনার শেষ গর্ভাবস্থা দিয়েই আলোচনা শুরু করতে চাই। *[যমজ বা একই সাথে হওয়া তার চেয়ে বেশী সংখ্যক সন্তানের ক্ষেত্রে আলাদা লাইন ব্যবহার করুন]*

Now I would like to talk to you about all of your pregnancies, whether the child was born alive, born dead or the pregnancy was lost before full-term, which is as a miscarriage or an abortion. (I would like to start with your last pregnancy. Record Twins and Triplets on separate lines.)

<table>
<tr><th>আপনার শেষ/পূর্ববর্তী গর্ভাবস্থার কথা চিন্তা করুন</th><th>এই গর্ভে কয়টি বাচ্চা ছিল? এক বা একাধিক (যেমন: যমজ)?
Single/multiple Pregnancy</th><th>কোন বছরের কোন মাসের কত তারিখে এই গর্ভাবস্থা শেষ হয়েছিল?
Date of pregnancy Outcome</th><th>এই গর্ভাবস্থার ফলাফল কি ছিল? জীবিত শিশু নাকি মৃতশিশু নাকি মেয়াদের আগে (২৮ সপ্তাহ) নষ্ট হওয়া গর্ভ যেমন: গর্ভনষ্ট বা গর্ভপাত?
Status of pregnancy Outcome</th><th>জন্মের পর বাচ্চাটি কি কেঁদেছিল বা নড়াচড়া করেছিল বা শ্বাসপ্রশ্বাস নিয়েছিল?
Cry/move/breathe after birth</th><th>বাচ্চাটির কি নাম দেয়া হয়েছিল ?
[যদি নাম না থাকে তবে XX লিখুন]
Name of the child</th><th>(নাম) কি মেয়ে না ছেলে?
Sex</th><th>(নাম) কি এখনও জীবিত?
Status of the child (alive or dead)</th><th>যদি জীবিত হয়:
সর্বশেষ বা গত জন্মদিনে (নাম) এর বয়স কত ছিল?
[পূর্ণ বছরের হিসাবে বয়স লিখুন। 1 বছরের কম হলে 00 লিখুন।]</th><th>যদি মৃত হয়: মৃত্যুর সময় তার বয়স কত ছিল?
[দুই মাস (60 দিন) এর কম হলে দিনে, পাঁচ বছরের কম হলে মাসে, পাঁচ বছরের বেশী হলে বছরে লিখুন]</th></tr>
<tr><th>D13</th><th>D14</th><th>D15</th><th>D16</th><th>D17</th><th>D18</th><th>D19</th><th>D20</th><th>D21</th><th>D22</th></tr>
<tr><td><u>01|</u></td><td>একক Single 1
একাধিক Multiple...2
জানি না DK ....9</td><td>Day .... |___|___|
Month .... |___|___|
Year .....|___|___|___|___|</td><td>জীবিত জন্ম Live birth............1
(go to D18)
মৃত জন্ম. Still Birth...............2
মেয়াদের আগে নষ্ট Abortion.....3
(go to D24)</td><td>হ্যাঁ Yes 1
না No 2
(go to D24)</td><td>________
নাম Name</td><td>ছেলে Boy.......... 1
মেয়ে Girl .... 2</td><td>হ্যাঁ Yes 1
না No 2
(go to D22)</td><td>বয়স AGE |___|___|
(যদি আর কোন গর্ভের ইতিহাস না থাকে তবে SKIP to D 24)</td><td>দিন Days.......... 1 |___|___|
মাস Months ..... 2 |___|___|
বছর Years .... 3 |___|___|
(যদি আর কোন গর্ভের ইতিহাস না থাকে তবে skip to D24)</td></tr>
<tr><td><u>02|</u></td><td>একক Single 1
একাধিক Multiple...2
জানি না DK ....9</td><td>Day .... |___|___|
Month .... |___|___|
Year .....|___|___|___|___|</td><td>জীবিত জন্ম Live birth............1
(go to D18)
মৃত জন্ম. Still Birth...............2
মেয়াদের আগে নষ্ট Abortion.....3
(go to D24)</td><td>হ্যাঁ Yes 1
না No 2
(go to D24)</td><td>________
নাম Name</td><td>ছেলে Boy.......... 1
মেয়ে Girl .... 2</td><td>হ্যাঁ Yes 1
না No 2
(go to D22)</td><td>বয়স AGE |___|___|
(যদি আর কোন গর্ভের ইতিহাস না থাকে তবে SKIP to D 24)</td><td>দিন Days.......... 1 |___|___|
মাস Months ..... 2 |___|___|
বছর Years .... 3 |___|___|
(যদি আর কোন গর্ভের ইতিহাস না থাকে তবে skip to D24)</td></tr>
<tr><td><u>03|</u></td><td>একক Single 1
একাধিক Multiple...2
জানি না DK ....9</td><td>Day .... |___|___|
Month .... |___|___|
Year .....|___|___|___|___|</td><td>জীবিত জন্ম Live birth............1
(go to D18)
মৃত জন্ম. Still Birth...............2
মেয়াদের আগে নষ্ট Abortion.....3
(go to D24)</td><td>হ্যাঁ Yes 1
না No 2
(go to D24)</td><td>________
নাম Name</td><td>ছেলে Boy.......... 1
মেয়ে Girl .... 2</td><td>হ্যাঁ Yes 1
না No 2
(go to D22)</td><td>বয়স AGE |___|___|
(যদি আর কোন গর্ভের ইতিহাস না থাকে তবে SKIP to D 24)</td><td>দিন Days.......... 1 |___|___|
মাস Months ..... 2 |___|___|
বছর Years .... 3 |___|___|
(যদি আর কোন গর্ভের ইতিহাস না থাকে তবে skip to D24)</td></tr>
<tr><td><u>04|</u></td><td>একক Single 1
একাধিক Multiple...2
জানি না DK ....9</td><td>Day .... |___|___|
Month .... |___|___|
Year .....|___|___|___|___|</td><td>জীবিত জন্ম Live birth............1
(go to D18)
মৃত জন্ম. Still Birth...............2
মেয়াদের আগে নষ্ট Abortion.....3
(go to D24)</td><td>হ্যাঁ Yes 1
না No 2
(go to D24)</td><td>________
নাম Name</td><td>ছেলে Boy.......... 1
মেয়ে Girl .... 2</td><td>হ্যাঁ Yes 1
না No 2
(go to D22)</td><td>বয়স AGE |___|___|
(যদি আর কোন গর্ভের ইতিহাস না থাকে তবে SKIP to D 24)</td><td>দিন Days.......... 1 |___|___|
মাস Months ..... 2 |___|___|
বছর Years .... 3 |___|___|
(যদি আর কোন গর্ভের ইতিহাস না থাকে তবে skip to D24)</td></tr>
<tr><td><u>05|</u></td><td>একক Single 1
একাধিক Multiple...2
জানি না DK ....9</td><td>Day .... |___|___|
Month .... |___|___|
Year .....|___|___|___|___|</td><td>জীবিত জন্ম Live birth............1
(go to D18)
মৃত জন্ম. Still Birth...............2
মেয়াদের আগে নষ্ট Abortion.....3
(go to D24)</td><td>হ্যাঁ Yes 1
না No 2
(go to D24)</td><td>________
নাম Name</td><td>ছেলে Boy.......... 1
মেয়ে Girl .... 2</td><td>হ্যাঁ Yes 1
না No 2
(go to D22)</td><td>বয়স AGE |___|___|
(যদি আর কোন গর্ভের ইতিহাস না থাকে তবে SKIP to D 24)</td><td>দিন Days.......... 1 |___|___|
মাস Months ..... 2 |___|___|
বছর Years .... 3 |___|___|
(যদি আর কোন গর্ভের ইতিহাস না থাকে তবে skip to D24)</td></tr>
</table>

<table>
<tr>
<th>আপনার শেষ/পূর্ববর্তী গর্ভাবস্থার কথা চিন্তা করুন</th>
<th>এই গর্ভে কয়টি বাচ্চা ছিল? এক বা একাধিক (যেমন: যমজ)?
Single/multiple Pregnancy</th>
<th>কোন বছরের কোন মাসের কত তারিখে এই গর্ভাবস্থা শেষ হয়েছিল?
Date of pregnancy Outcome</th>
<th>এই গর্ভাবস্থার ফলাফল কি ছিল? জীবিত শিশু নাকি মৃতশিশু নাকি মেয়াদের আগে (২৮ সপ্তাহ) নষ্ট হওয়া গর্ভ যেমন: গর্ভনষ্ট বা গর্ভপাত?
Status of pregnancy Outcome</th>
<th>জন্মের পর বাচ্চাটি কি কেঁদেছিল বা নড়াচড়া করেছিল বা শ্বাসপ্রশ্বাস নিয়েছিল?
Cry/move/breathe after birth</th>
<th>বাচ্চাটির কি নাম দেয়া হয়েছিল ?
[যদি নাম না থাকে তবে XX লিখুন]
Name of the child</th>
<th>(নাম) কি মেয়ে না ছেলে?
Sex</th>
<th>(নাম) কি এখনও জীবিত?
Status of the child (alive or dead)</th>
<th>যদি জীবিত হয়:
সর্বশেষ বা গত জন্মদিনে (নাম) এর বয়স কত ছিল?
[পূর্ণ বছরের হিসাবে বয়স লিখুন। 1 বছরের কম হলে 00 লিখুন।]</th>
<th>যদি মৃত হয়: মৃত্যুর সময় তার বয়স কত ছিল?
[দুই মাস (60 দিন) এর কম হলে দিনে, পাঁচ বছরের কম হলে মাসে, পাঁচ বছরের বেশী হলে বছরে লিখুন]</th>
</tr>
<tr><td>D13</td><td>D14</td><td>D15</td><td>D16</td><td>D17</td><td>D18</td><td>D19</td><td>D20</td><td>D21</td><td>D22</td></tr>
<tr>
<td>06|</td>
<td>একক Single 1
একাধিক Multiple...2
জানি না DK...........9</td>
<td>Day .... |___|___|
Month .... |___|___|
Year
.....|___|___|___|___|</td>
<td>জীবিত জন্ম Live birth...........1
(go to D18)
মৃত জন্ম. Still Birth..............2
মেয়াদের আগে নষ্ট Abortion.....3
(go to D24)</td>
<td>হ্যাঁ Yes 1
না No 2
(go to D24)</td>
<td>______________
নাম Name</td>
<td>ছেলে Boy.......... 1
মেয়ে Girl.......... 2</td>
<td>হ্যাঁ Yes 1
না No 2
(go to D22)</td>
<td>বয়স AGE |___|___|
(যদি আর কোন গর্ভের ইতিহাস না থাকে তবে SKIP to D 24)</td>
<td>দিন Days.......... 1 |___|___|
মাস Months ..... 2 |___|___|
বছর Years .... 3 |___|___|
(যদি আর কোন গর্ভের ইতিহাস না থাকে তবে skip to D24)</td>
</tr>
<tr>
<td>07|</td>
<td>একক Single 1
একাধিক Multiple...2
জানি না DK...........9</td>
<td>Day .... |___|___|
Month .... |___|___|
Year
.....|___|___|___|___|</td>
<td>জীবিত জন্ম Live birth...........1
(go to D18)
মৃত জন্ম. Still Birth..............2
মেয়াদের আগে নষ্ট Abortion.....3
(go to D24)</td>
<td>হ্যাঁ Yes 1
না No 2
(go to D24)</td>
<td>______________
নাম Name</td>
<td>ছেলে Boy.......... 1
মেয়ে Girl.......... 2</td>
<td>হ্যাঁ Yes 1
না No 2
(go to D22)</td>
<td>বয়স AGE |___|___|
(যদি আর কোন গর্ভের ইতিহাস না থাকে তবে SKIP to D 24)</td>
<td>দিন Days.......... 1 |___|___|
মাস Months ..... 2 |___|___|
বছর Years .... 3 |___|___|
(যদি আর কোন গর্ভের ইতিহাস না থাকে তবে skip to D24)</td>
</tr>
<tr>
<td>08|</td>
<td>একক Single 1
একাধিক Multiple...2
জানি না DK...........9</td>
<td>Day .... |___|___|
Month .... |___|___|
Year
.....|___|___|___|___|</td>
<td>জীবিত জন্ম Live birth...........1
(go to D18)
মৃত জন্ম. Still Birth..............2
মেয়াদের আগে নষ্ট Abortion.....3
(go to D24)</td>
<td>হ্যাঁ Yes 1
না No 2
(go to D24)</td>
<td>______________
নাম Name</td>
<td>ছেলে Boy.......... 1
মেয়ে Girl.......... 2</td>
<td>হ্যাঁ Yes 1
না No 2
(go to D22)</td>
<td>বয়স AGE |___|___|
(যদি আর কোন গর্ভের ইতিহাস না থাকে তবে SKIP to D 24)</td>
<td>দিন Days.......... 1 |___|___|
মাস Months ..... 2 |___|___|
বছর Years .... 3 |___|___|
(যদি আর কোন গর্ভের ইতিহাস না থাকে তবে skip to D24)</td>
</tr>
<tr>
<td>09|</td>
<td>একক Single 1
একাধিক Multiple...2
জানি না DK...........9</td>
<td>Day .... |___|___|
Month .... |___|___|
Year
.....|___|___|___|___|</td>
<td>জীবিত জন্ম Live birth...........1
(go to D18)
মৃত জন্ম. Still Birth..............2
মেয়াদের আগে নষ্ট Abortion.....3
(go to D24)</td>
<td>হ্যাঁ Yes 1
না No 2
(go to D24)</td>
<td>______________
নাম Name</td>
<td>ছেলে Boy.......... 1
মেয়ে Girl.......... 2</td>
<td>হ্যাঁ Yes 1
না No 2
(go to D22)</td>
<td>বয়স AGE |___|___|
(যদি আর কোন গর্ভের ইতিহাস না থাকে তবে SKIP to D 24)</td>
<td>দিন Days.......... 1 |___|___|
মাস Months ..... 2 |___|___|
বছর Years .... 3 |___|___|
(যদি আর কোন গর্ভের ইতিহাস না থাকে তবে skip to D24)</td>
</tr>
<tr>
<td>10|</td>
<td>একক Single 1
একাধিক Multiple...2
জানি না DK...........9</td>
<td>Day .... |___|___|
Month .... |___|___|
Year
.....|___|___|___|___|</td>
<td>জীবিত জন্ম Live birth...........1
(go to D18)
মৃত জন্ম. Still Birth..............2
মেয়াদের আগে নষ্ট Abortion.....3
(go to D24)</td>
<td>হ্যাঁ Yes 1
না No 2
(go to D24)</td>
<td>______________
নাম Name</td>
<td>ছেলে Boy.......... 1
মেয়ে Girl.......... 2</td>
<td>হ্যাঁ Yes 1
না No 2
(go to D22)</td>
<td>বয়স AGE |___|___|
(যদি আর কোন গর্ভের ইতিহাস না থাকে তবে SKIP to D 24)</td>
<td>দিন Days.......... 1 |___|___|
মাস Months ..... 2 |___|___|
বছর Years .... 3 |___|___|
(যদি আর কোন গর্ভের ইতিহাস না থাকে তবে skip to D24)</td>
</tr>
</table>

N0.	QUESTIONS AND FILTERS	CODING CATEGORIES	SKIP TO
D24	*[D10 চেক করুন এবং সংখ্যাটি এখানে লিখুন (Check D10 and write the number here)]*	|____|____|	
	[কলাম D13 চেক করুন এবং মোট কতটি লাইন পূরন করা হয়েছে - সেই সংখ্যাটি এখানে লিখুন] (Check Collumn D13 and write the number of lines filled in)	|____|____|	
	[উপরের সংখ্যাগুলো তুলনা করুন এবং "টিক" চিহ্ন দিন (Compare the numbes above above and put tick (√)]:		
	সংখ্যাগুলো একই (numbers are same) ☐ ↓	*সংখ্যাগুলো ভিন্ন (numbers are different)* ☐ *[PROBE করুন এবং সংশোধন করুন]*	
D25	*চেক করুন এবং "টিক" চিহ্ন দিন:*		
	প্রতিটি গর্ভ ফলাফলের জন্য মাস এবং সাল রেকর্ড করা হয়েছে (For each pregnancy outcome: month and year of birth are recorded)	☐	
	প্রতিটি জীবিত সন্তানের ক্ষেত্রে: বর্তমান বয়স রেকর্ড করা হয়েছে (current age of all alive children has been recorded)	☐	
	প্রতিটি মৃত সন্তানের ক্ষেত্রে: মৃত্যুর সময়কার বয়স রেকর্ড করা হয়েছে (age at death for all dead children has been recoded)	☐	
	মৃত্যুর সময় বয়স ৫ বছর -এর কম হলে প্রোব করুন এবং নিশ্চিত হয়ে নিন যে সঠিকভাবে মাসের সংখ্যা রেকর্ড করা হয়েছে (If age at death is less than 5 years, then probe and assure, number of months has been recorded correctly)	☐	
	***মৃত্যুর সময় বয়স 2 মাস** (60 দিন) - **এর কম হলে** প্রোব করুন এবং নিশ্চিত হয়ে নিন যে সঠিকভাবে **দিনের** সংখ্যা রেকর্ড করা হয়েছে: (If age at death is less than 2 months (60 days), then probe and assure number of days has been recorded correctly)*	☐	

Interviewer:

[D15, D16 এবং D20 দেখুন এবং সঠিক ঘরে টিক দিন]

Check the questions and its answers from D15, D16 and D20; and tick the right box below

1.	মহিলার 1 (পহেলা) জুন 2011 সাল বা তার পরে কমপক্ষে একবার গর্ভ ফলাফলের (জীবিত জন্ম/মৃত জন্ম/গর্ভপাত) ইতিহাস আছে। The women has at least one Pregnancy outcome history since June 2011	হ্যাঁ Yes....................................... 1<br>না No... 2	→E00
2.	মহিলার 1 (পহেলা) জুন 2007 সালে বা তার পর জন্ম নেয়া কমপক্ষে একটি পাঁচ বছর বা তার কম বয়সী জীবিত বাচ্চা আছে। The women has at least one living (live birth and still alive) child aged 5 yrs or below (DOB since June 2007)	হ্যাঁ Yes...................................... 1<br>না No 2	→I00<br>→ *

* উত্তরদাত্রীকে সহযোগিতা করার জন্য ধন্যবাদ দিন। যদি এক এর অধিক মহিলা থাকেন তবে পরের মহিলার ইন্টারভিউ শুরু করুন।

Module III

Antenatal, Delivery, Immediate Newborn and Post Natal Care

[পহেলা জুন 2008 বা তার পরের কোন তারিখে উত্তরদাত্রীর কোন গর্ভের ফলাফল থেকে থাকলে, সেই (সর্বশেষ) গর্ভাবস্থার গর্ভকালীন, প্রসবকালীন ও প্রসব পরবর্তীকালীন সেবা সম্পর্কে আপনাকে এই মডিয়ুলে তথ্য সংগ্রহ করতে হবে (গর্ভের ফলাফল জীবিত জন্ম বা মৃত জন্ম যাই হোক না কেন)। সর্বশেষ গর্ভের ফলাফল যদি জীবিত জন্ম/শিশু হয়ে থাকে, তাহলে সেই শিশুর Immediate Newborn *বিষয়ক তথ্যও সংগ্রহ করতে হবে। কাজেই নিশ্চিত হয়ে নিন যে, উত্তরদাত্রী 'সর্বশেষ গর্ভাবস্থার কথা' বুঝতে পেরেছেন এবং সুনির্দিষ্ট করতে পেরেছেন । এখন প্রশ্ন করা শুরু করুনঃ]*

[You have to collect information regarding care during pregnancy, during delivery and during post partum time of the woman's most recent pregnancy outcome (Live birth or Still Birth since June 2008) and also immediate newborn care of most recent delivery of live birth since June 2008. So, make sure that the respondent understand and identify the most recent pregnancy then ask questions.]

No	Questions and filters	Options and coding category	Skip
E00	*[প্রশ্ন নং D15 দেখুন এবং উত্তরদাত্রীর সর্বশেষ গর্ভের ফলাফলের /গর্ভশেষ হওয়ার তারিখ লক্ষ্য করুন]*<br>আপনার সর্বশেষ গর্ভের ফলাফল/গর্ভশেষ হওয়ার তারিখ কি ১লা জুন 2008 বা তার পরের কোন তারিখে?<br>Check D15. Find out the date of delivery of the last pregnancy outcome of the women. Did the last pregnancy outcome of the woman take place in 01 June 2008 or later?	হ্যাঁ , সর্বশেষ গর্ভের ফলাফলের তারিখ 1 লা (পহেলা) জুন 2011 বা তার পরের কোন তারিখে Yes, Last outcome in 1 June 11 or later..........................1<br>না, সর্বশেষ গর্ভের , ফলাফলের তারিখ 1 লা (পহেলা) জুন 2011 এর আগের কোন তারিখে No, last outcome was before 1 June 11........................2	 <br><br>→ I00a
E00a	*[Q D15 এবং D16 দেখুন]* [মহিলার ১লা জুন, 2011 বা এর পরে হওয়া সর্বশেষ গর্ভের ফলাফল কি ছিল?]<br>Check Q D15 and D16 for the answer. What was the outcome of your most recent pregnancy since June 2011.	জীবিত জন্ম Live Birth ....1<br>মৃত জন্ম Still Birth..2<br>গর্ভপাত/গর্ভনষ্ট Abortion 3	 <br>→ I00a

Section E: Antenatal Care

This Section contains some information regarding care of the mother during her most recent pregnancy

No	Questions and filters	Options and coding category	Skip
E01	গর্ভকালীন সময়ে একজন মহিলা অসুস্থ না হলেও মেডিকেল চেকআপ (ANC) করার দরকার আছে কি?<br>Do you know about ANC (Ante natal care), a medical check-up for a woman even when she is not sick, during pregnancy?	হ্যাঁ Yes..1<br>না No 2	 →E06
E02	গর্ভকালীন সময়ে একজন মহিলার কমপক্ষে কত বার মেডিকেল চেকআপ (ANC) করা দরকার?<br>Can you tell me at least how many times a pregnant woman should receive such kind of medical check-up (ANC) during pregnancy?	বার (গর্ভকালীন সময়ে) Times (during pregnancy)..................... |____|____| জানা নাই/মনে নাই DK/can't remember 99	
E03	গর্ভের কত মাসের সময় একজন মহিলার প্রথম মেডিকেল চেকআপ (ANC) করা দরকার?<br>When should a pregnant woman first receive antenatal care (at what time in her pregnancy)?	মাস (গর্ভকালীন সময়ে) Months (during pregnancy)................... |____|____| জানা নাই/মনে নাই DK/can't remember 99	

No	Questions and filters	Options and coding category	Skip
E04	একজন মহিলা গর্ভকালীন সময়ে মেডিকেল চেক-আপ (ANC) করার জন্য কোথায় যেতে পারেন, সেটা কি আপনি জানেন? আমাকে কি বলবেন কোথায় কোথায় সেবার জন্য সেই সময়টায় যাওয়া যায়?<br><br>*[মহিলাকে জিজ্ঞেস করুন]* আরও কিছু? *[মহিলার নিজে থেকে দেয়া সবগুলো উত্তরই বৃত্তায়িত করুন । উত্তরগুলো পড়ে শুনাবেন না। একাধিক উত্তর হতে পারে।]*<br><br>Can you tell us from where you can get antenatal check-up? *[Ask the women, Anything else? Record all the un-prompted answer.]*	**<u>সরকারী স্বাস্থ্য কেন্দ্র (Govt Health center)</u>**<br>মেডিকেল কলেজ হাসপাতাল (Medical College Hospital) .... A<br>জেলা/সদর হাসপাতাল (District /Sadar Hospital) .... B<br>মা ও শিশু স্বাস্থ্য কেন্দ্র (MCWC) .... C<br>উপজেলা স্বাস্থ্য কমপ্লেক্স (UHC) .... D<br>ইউনিয়ন স্বাস্থ্য ও পরিবার কল্যাণ কেন্দ্র/সাব সেন্টার/আরডি (FWC/SC/RD) ... E<br>কমিউনিটি ক্লিনিক (Community clinic) .... F<br>সেটেলাইট ক্লিনিক/ ইপিআই কেন্দ্র (Satellite clinic/EPI centre) .... G<br>অন্যান্য সরকারী স্বাস্থ্য কেন্দ্র (Other Govt Health facility) .... H<br>**<u>বেসরকারী স্বাস্থ্য কেন্দ্র (Non Govt Health center)</u>**<br>এনজিও হাসপাতাল (NGO hospital) .... I<br>এনজিও স্থায়ী স্বাস্থ্য কেন্দ্র (NGO static health centre) .... J<br>এনজিও সেটেলাইট ক্লিনিক (NGO satellite clinic) .... K<br>পুষ্টি কেন্দ্র (NNP centre) .... L<br>অন্যান্য বেসরকারী স্বাস্থ্য কেন্দ্র (Other NGO Health facility) .... M<br>**<u>প্রাইভেট (Private Health sector)</u>**<br>হাসপাতাল/ ক্লিনিক (Hospital/clinic) .... N<br>স্বাস্থ্য কেন্দ্র /ডিসপেনসারী (Health centre/Dispensary) .... O<br>এমবিবিএস ডাক্তারের চেম্বার (MBBS doctor's chamber) .... P<br>গ্রাম ডাক্তারের চেম্বার (Village doctor's chamber) .... Q<br>প্যারামেডিক/মেডিকেল এসিসটেন্ট/সাকমোর চেম্বার (Paramedic/ MA/SACMO chamber) R<br>এলোপ্যাথী ঔষধের দোকান (Allopath drug store) .... S<br>অন্যান্য প্রাইভেট স্বাস্থ্য কেন্দ্র (Other private Health facility) .... T<br>অন্যান্য (Others) ________________ X<br>(নির্দিষ্ট করুন)<br>কোথায় যেতে হবে, জানা নাই (Don't know) .... Y	
E05	*[যদি মা উপরের প্রশ্নের (E02-E04) এক বা একাধিক উত্তর হ্যাঁ সূচক বলেন, তাহলে মাকে জিজ্ঞেস করুন]*<br><br>এই যে আপনি বললেন যে, একজন গর্ভবতী মহিলার জন্য মেডিকেল চেকআপ/সেবা (ANC) নেয়া উচিত, এবং তা ---- বার এবং ------ -এর কাছ থেকে নিতে হবে/নেয়া উচিত - এই তথ্য আপনি কোথা থেকে বা কার কাছ থেকে জেনেছেন/পেয়েছেন?<br><br>*[মহিলাকে জিজ্ঞেস করুন]* আরও কিছু? *[মহিলার নিজে থেকে দেয়া সবগুলো উত্তরই বৃত্তায়িত করুন । উত্তরগুলো পড়ে শুনাবেন না। একাধিক উত্তর হতে পারে।]*<br><br>[If the women answer any one of the questions above, then ask the women: Where did you get this information?]	ডাক্তার/নার্স/ধাত্রী/প্যারামেডিক (Doctor/ Nurse/Midwife/ Paramedics) .... A<br>স্বাস্থ্য কেন্দ্র/হাসপাতাল (Health facility/ Clinic/ Hospital) .... B<br>কমিউনিটি স্বাস্থ্য কর্মী - স্বাস্থ্য সহকারী/পরিবার কল্যান সহকারী, পুষ্টি কর্মী, এনজিও কর্মী, MNCS প্রমোটর, স্বেচ্ছাসেবী (CHWs -HAS, FWAs, CNPs, MNCS promoter,NGO worker, volunteer) .... C<br>কমিউনিটি গ্রুপ মিটিং / মিটিং /সভা থেকে (Community meeting) .... D<br>রেডিও/টিভি (Radio /TV) .... E<br>সংবাদপত্র/খবরের কাগজ/বইপত্র (Newspaper/Books) .... F<br>পোষ্টার / কোন ছাপানো কাগজ (Poster/Leaflet) .... G<br>পরিবারের অন্যান্য সদস্য/আত্মীয়/প্রতিবেশী/বন্ধু Family/relatives/Neighbor/friend ..... H<br>অন্যান্য Others________________ ... X<br>(নির্দিষ্ট করুন)<br>মনে নাই Can't remember .... Y	
E06	শেষ যখন আপনি গর্ভবতী ছিলেন, তখন গর্ভকালীন মেডিকেল চেক-আপের জন্য আপনি কি কাউকে দেখিয়েছিলেন?<br>Did you see (consult) anyone for ANC (health check-up) during your most recent pregnancy?	হ্যাঁ Yes .... 1<br>না No .... 2<br>জানি না/ মনে নাই Don't know/Can't remember 9	→E08<br><br>→E14

No	Questions and filters	Options and coding category	Skip
E07	শেষ যখন আপনি গর্ভবতী ছিলেন, তখন গর্ভকালীন মেডিকেল চেক-আপের জন্য আপনি কেন কাউকে দেখান নি?<br><br>*[মহিলাকে জিজ্ঞেস করুন]* আরও কিছু? *[মহিলার নিজে থেকে দেয়া সবগুলো উত্তরই বৃত্তায়িত করুন । উত্তরগুলো পড়ে শুনাবেন না। একাধিক উত্তর হতে পারে।]*<br><br>If the women didn't seek ANC then ask, why she didn't seek ANC from anyone? *[Do not readout the answers. Ask anyone else?Record all the un-prompted answers]*	সেবার প্রয়োজন আছে বলে মনে হয় নি/সেবার প্রয়োজন নেই<br>Didn't think it was necessary to seek care .... A<br>জানতাম না কোথায় যেতে হবে Not known where to go .... B<br>অনেক খরচ/ টাকা পয়সা ছিল না Too costly/ Lack of money .... C<br>স্বাস্থ্য কেন্দ্র বাসা হতে অনেক দুরে Too far from house .... D<br>যানবাহনের সমস্যা Transport problem .... E<br>সাথে যাবার মত কেউ ছিল না No one accompanied .... F<br>স্বাস্থ্যকেন্দ্রে যাবার মত সময় ছিল না Not enough time to go .... G<br>পরিবার আমাকে যেতে দেন নি Family didn't allow me to go .... H<br>ধর্মে মানা/বাধা Religeous bar .... I<br>স্বাস্থ্যকেন্দ্রের সেবাদানের সময় সীমা সুবিধাজনক নয় Service hr inconvenient ... J<br>স্বাস্থ্যকেন্দ্র বন্ধ ছিল/কোন স্বাস্থ্যকর্মী ছিলেন না HF found closed/nobody there..... K<br>স্বাস্থ্যকেন্দ্রে সেবা অনুন্নত মানের Poor quality of services at facility .... L<br>স্বাস্থ্যকেন্দ্রে নিম্নমানের এবং অদক্ষ সেবাপ্রদানকারী Poor quality & staffs at HF .. M<br>স্বাস্থ্যকেন্দ্রে পর্দার অভাব Lack of privacy .... N<br>স্বাস্থ্যকেন্দ্রের সেবাদানকারীদের ব্যবহার খারাপ Unpleasant behavior at center ... O<br>স্বাস্থ্যকেন্দ্রের অনেকক্ষন বসে থাকতে হয় সেবা পাবার জন্য Long queue at HF . P<br>স্বাস্থ্যকেন্দ্রের ঔষধ পত্র পাওয়া যায়না Inadequate drugs at the health center .... Q<br>অন্যান্য Others ________________ .. X<br>(নির্দিষ্ট করুন)	Skip to E14 for all
E08	শেষ যখন আপনি গর্ভবতী ছিলেন, তখন গর্ভকালীন মেডিকেল চেক-আপের জন্য আপনি কাকে দেখিয়েছিলেন?<br><br>*[মহিলাকে জিজ্ঞেস করুন]* আরও কিছু? *[মহিলার নিজে থেকে দেয়া সবগুলো উত্তরই বৃত্তায়িত করুন] । উত্তরগুলো পড়ে শুনাবেন না। একাধিক উত্তর হতে পারে।]*<br><br>*[যদি মহিলার উত্তর সি,এস,বি,এ (Code E) বা MNCS প্রমোটর (Code H) হয়, তাহলে তাদের নাম লিখুন।]*<br><br>**Code :E** নাম Name: ________________<br><br>**Code :H** নাম Name: ________________<br><br>To whom you seek care for ANC during you most recent pregnancy? *[Do not readout the answers. Ask anyone else? Record all the answers. If the answer is either E (CSBA) or H (MNCS promoter), then please collect the name of the health workers.]*	**দক্ষ/প্রশিক্ষণ প্রাপ্ত স্বাস্থ্য কর্মী (Medically trained)**<br>পাশ করা ডাক্তার (MBBS doctor) .... A<br>নার্স/ধাত্রী (Nurse/midwife) .... B<br>প্যারামেডিক/মেডিকেল এসিসটেন্ট/সাকমো (Paramedic/MA/SACMO) .... C<br>পরিবার কল্যাণ পরিদর্শক (FWV) .... D<br>সি,এস,বি,এ (CSBA) .... E<br>**অন্যান্য স্বাস্থ্য কর্মী (Other health worker)**<br>স্বাস্থ্য সহকারী/ পরিবার কল্যান সহকারী (HA /FWA) .... F<br>পুষ্টি কর্মী (CNP) .... G<br>MNCS প্রমোটর (MNCS Promoter) .... H<br>অন্যান্য কমিউনিটি স্বাস্থ্য কর্মী - এনজিও কর্মী, স্বেচ্ছাসেবী (Other CHWs, NGO worker, volunteer) .... I<br>**অন্যান্য (Other)**<br>প্রশিক্ষণ প্রাপ্ত টিবিএ (প্রশিক্ষণ প্রাপ্ত ধন্নী, চাউনী, দাই) (TTBA) .... J<br>প্রশিক্ষণহীন টিবিএ (ধন্নী, চাউনী, দাই) TBA(Dai/Dhorni/Chauni) .... K<br>হোমিওপ্যাথ/হোমিওপ্যাথ ঔষধের দোকান (Homeopath/Homeopath drug store) .... L<br>আয়ুর্বেদিক চিকিৎসক / আয়ুর্বেদিক ঔষধের দোকান /হেকিম/কবিরাজ (Ayurved/ Ayurvedic drug store /Hekim/Kabiraj) .... M<br>গ্রাম ডাক্তার (Village doctor) .... N<br>এলোপ্যাথী ঔষধের দোকান (Allopath drug store) .... O<br>ইমাম/ঝাড় ফুক/ওঝা (Spiritual healer) .... P<br>পরিবারের অন্যান্য সদস্য/আত্মীয়/ প্রতিবেশী/বন্ধু Family/relative/Neighbor/friend .... Q<br>অন্যান্য Others ________________ .. X<br>(নির্দিষ্ট করুন)<br>জানি না/মনে নাই Don't know/can't remember .... Y	

No	Questions and filters	Options and coding category	Skip
E09	শেষ যখন আপনি গর্ভবতী ছিলেন, তখন গর্ভকালীন মেডিকেল চেক-আপ আপনি কোথায় নিয়েছিলেন/করিয়েছিলেন?<br><br>*[মহিলাকে জিজ্ঞেস করুন]* আরও কিছু? *[মহিলার নিজে থেকে দেয়া সবগুলো উত্তরই বৃত্তায়িত করুন । উত্তরগুলো পড়ে শুনাবেন না। একাধিক উত্তর হতে পারে।]*<br><br>*[মহিলা যেখান থেকে সেবা পেয়েছেন, সেই স্বাস্থ্যকেন্দ্রের নাম লিখুন। যদি একাধিক জায়গা থেকে সেবা নিয়ে থাকেন, তাহলে সবগুলো জায়গারই নাম এবং কোড লিখুন।]*<br><br>Code :|____| নাম Name: ______________________________<br><br>Code :|____| নাম Name: ______________________________<br><br>Code :|____| নাম Name: ______________________________<br><br>From where did you receive Antenatal Check-up? Do not readout the answers. Ask anywhere else? Record all the answers. Record the name of the places where the women seek care.	**বাড়ী (Home)**<br>নিজ বাড়ী, স্বামী/শ্বশুড় বাড়ী (Own home, husband/father in laws house)........A<br>বাবার বাড়ী (My natal home) ....B<br>অন্য কোন বাড়ী (Others)________________________C<br>(নির্দিষ্ট করুন)<br>**সরকারী স্বাস্থ্য কেন্দ্র (Govt Health center)**<br>মেডিকেল কলেজ হাসপাতাল (Medical College Hospital) ....D<br>জেলা/সদর হাসপাতাল (District /Sadar Hospital) ....E<br>মা ও শিশু স্বাস্থ্য কেন্দ্র (MCWC)..F<br>উপজেলা স্বাস্থ্য কমপ্লেক্স (UHC) ....G<br>ইউনিয়ন স্বাস্থ্য ও পরিবার কল্যাণ কেন্দ্র / সাব সেন্টার/আরডি (FWC/SC/RD) H<br>কমিউনিটি ক্লিনিক (Community clinic)..I<br>সেটেলাইট ক্লিনিক/ ইপিআই কেন্দ্র (Satellite clinic/EPI centre)...................J<br>অন্যান্য সরকারী স্বাস্থ্য কেন্দ্র (Other Govt Health facility) ....K<br>**বেসরকারী স্বাস্থ্য কেন্দ্র (Non Govt Health center)**<br>এনজিও হাসপাতাল (NGO hospital)...L<br>এনজিও স্থায়ী স্বাস্থ্য কেন্দ্র (NGO static health centre) ....M<br>এনজিও সেটেলাইট ক্লিনিক (NGO satellite clinic)................................N<br>পুষ্টি কেন্দ্র (NNP centre)..O<br>অন্যান্য বেসরকারী স্বাস্থ্য কেন্দ্র (Other NGO Health facility).....................P<br>**প্রাইভেট (Private Health sector)**<br>হাসপাতাল/ ক্লিনিক (Hospital/clinic)...Q<br>স্বাস্থ্য কেন্দ্র /ডিসপেনসারী (Health centre/Dispensary) ....R<br>এমবিবিএস ডাক্তারের চেম্বার (MBBS doctor's chamber).........................S<br>গ্রাম ডাক্তারের চেম্বার (Village doctor's chamber) ....T<br>প্যারামেডিক/মেডিকেল এসিসটেন্ট/সাকমোর চেম্বার (Paramedic/MA/SACMO chamber) U<br>এলোপ্যাথী ঔষধের দোকান (Allopath drug store) ....V<br>অন্যান্য প্রাইভেট_স্বাস্থ্য কেন্দ্র (Other private Health facility......................W<br>অন্যান্য (Others) _____________________________________ ..X<br>(নির্দিষ্ট করুন)	
E10	*[Question E08 দেখুন, code A থেকে E এর যে কোন একটি বা একাধিক বৃত্তায়িত থাকলে কোড/কোডগুলো এখানে লিখুন: _____ , _____, ____ । এবার কোড/কোডগুলো দেখে দেখে মহিলাকে প্রশ্ন করুন ঃ]*<br><br>আপনি বললেন যে, আপনি ----, -----, ---- এর/এদের কাছ থেকে গর্ভকালীন মেডিকেল চেক-আপ করেছিলেন/নিয়েছিলেন। এখন আমাকে বলুন যে,<br>আপনি এর/এদের কাছ থেকে মোট কতবার গর্ভকালীন মেডিকেল চেক-আপ করিয়েছিলেন/নিয়েছিলেন?<br>আপনি এর/এদের ভিতর যার কাছ থেকে প্রথম গর্ভকালীন মেডিকেল চেক-আপ করিয়েছিলেন/নিয়েছিলেন, তখন আপনি কত মাসের গর্ভবতী ছিলেন?<br><br>*[মহিলা যদি code A থেকে E এর ভিতর একাধিক স্বাস্থ্যকর্মীর থেকে সেবা গ্রহন করে থাকেন তাহলে তাদের সবার কথাই বলুন এবং তাদের সবার কাছ থেকে সব মিলিয়ে মোট কতবার গর্ভকালীন মেডিকেল চেক-আপ করিয়েছিলেন/নিয়েছিলেন তা লিখুন এবং যার কাছ থেকে সর্বপ্রথম চেক-আপ করিয়েছিলেন/নিয়েছিলেন তখন তিনি কতমাসের গর্ভবতী ছিলেন তা লিখুন।]*<br><br>How many times have you seek ANC to the mentioned skilled health care providers (Code A-E) during your most recent pregnancy? AND How many months pregnant were you when you FIRST seek ANC from the mentioned skilled health care provides (Code A-E)?	বার Times ....|____|____| <br>জানা নাই/মনে নাই DK/can't remember ....99<br><br>মাসের গর্ভবতী Month-pregnant (during 1st check-up) ....|____|____| <br>জানা নাই/মনে নাই DK/can't remember ....98<br><br>প্রযোজ্য নয় Not applicable 97	

No	Questions and filters	Options and coding category	Skip
E11	*[Question E08 দেখুন, code F থেকে R এর যেকোন একটি বা একাধিক বৃত্তায়িত থাকলে কোড/কোডগুলো এখানে লিখুন: _____ , _____, ____ । এবার কোড/কোডগুলো দেখে দেখে মহিলাকে প্রশ্ন করুন ঃ]*<br><br>আপনি বললেন যে, আপনি ----, -----, ---- এর/এদের কাছ থেকে গর্ভকালীন মেডিকেল চেক-আপ করেছিলেন/নিয়েছিলেন। এখন আমাকে বলুন যে,<br>আপনি এর/এদের কাছ থেকে মোট কতবার গর্ভকালীন মেডিকেল চেক-আপ করিয়েছিলেন/নিয়েছিলেন?<br><br>*[মহিলা যদি code F থেকে R এর ভিতর একাধিক স্বাস্থ্যকর্মীর থেকে সেবা গ্রহন করে থাকেন তাহলে তাদের সবার কথাই বলুন এবং তাদের সবার কাছ থেকে সব মিলিয়ে মোট কতবার গর্ভকালীন মেডিকেল চেক-আপ করেছিলেন/নিয়েছিলেন তা লিখুন]*<br><br>How many times have you seek ANC to mentioned personels/health workers (Code F-R) during your most recent pregnancy?	বার Times .... |____|____| <br>জানা নাই/মনে নাই DK/can't remember .... 99<br><br>প্রযোজ্য নয় Not applicable 97	
E12	গর্ভকালীন মেডিকেল চেক-আপের জন্য আপনি যাদের দেখিয়েছিলেন, তাদের কাছ থেকে কি কি উপদেশ আপনি পেয়েছিলেন?<br><br>*[মহিলাকে জিজ্ঞেস করুন]* আরও কিছু? *[মহিলার নিজে থেকে দেয়া সবগুলো উত্তরই বৃত্তায়িত করুন । উত্তরগুলো পড়ে শুনাবেন না। একাধিক উত্তর হতে পারে।]*<br><br>What sort of advices have you received from these people during your most recent pregnancy? Can you recall what advice the person gave you regarding care during pregnancy? *[Do not read the answers, Ask: is there anything else? Record all the un-prompted answers]*	**গর্ভকালীন যত্ন বিষয়ক (Pregnancy care)**<br>অতিরিক্ত ও পুষ্টিকর খাবার খাওয়ার কথা বলেছে take extra and nutritious food..... A<br>বেশী করে পানি খাওয়ার কথা বলেছে Advised to drink more water........ B<br>ব্যক্তিগত পরিষ্কার পরিচ্ছন্নতার কথা বলেছে Talked about personal hygience ... C<br>দিনের বেলা ২ ঘন্টা বিশ্রাম, ভারী কাজ না করা 2hr rest/avoid heavy work ..... D<br>টিটি নেয়ার কথা বলেছে Advised to take TT vaccination .... E<br>আয়রণ ট্যাবলেট খাওয়ার কথা বলেছে Advised to take Iron tablet........ F<br>গর্ভকালীন বিপদ চিহ্নের কথা জানিয়েছে danger signs during pregnancy........ G<br>গর্ভকালীন বিপদ চিহ্ন দেখা দিলে হাসপাতালে যাওয়া CareSeeking for Danger S .. H<br>**ডেলিভারী কালীন যত্ন বিষয়ক (Delivery care)**<br>জরুরী অবস্থার জন্য টাকা জমানোর কথা Savings for emergency .... I<br>জরুরী অবস্থায় যানবাহনের ব্যবস্থা করা Arrangement of transport for emergency..... J<br>প্রশিক্ষণপ্রাপ্ত সেবাদানকারী দিয়ে ডেলিভারী করানো Delivery by Skilled providers ...... K<br>আলো বাতাস পূর্ন স্থানে ডেলিভারী করা delivery at a well-lighted place........ L<br>**নবজাতকের যত্ন বিষয়ক (Neonatal care)**<br>দুই টুকরা কাপড় প্রস্তুত রাখার কথা বলেছে Advised to prepare 2 pieces of cloth ..... M<br>নবজাতকের যত্ন নেয়ার জন্য লোক ঠিক করার কথা বলেছে newborn care person N<br>নবজাতকের বিপদ চিহ্ন/লক্ষণ সম্পর্কে জেনেছিলাম danger signs of newborn . O<br>বুকের দুধ খাওয়ানোর কথা Breastfeeding .... P<br>কম ওজনের বাচ্চার যত্নের কথা LBW baby care........ Q<br>জন্মের পর পরই বাচ্চাকে পরিস্কার করা/শুকিয়ে নেয়ার কথা drying newborn .. R<br>জন্মের পর পরই বাচ্চাকে কাপড় দিয়ে মুড়িয়ে নেয়ার কথা wrapping newborn . S<br>নাভীতে কিছু না দেয়ার কথা Nothing to umbilicus........ T<br>ভারনিক্স/সাদা চামড়া না তোলার কথা Intact vernix .... U<br>প্রসবের ৩ দিন/৭২ ঘন্টা পর প্রথম গোসল করানোর কথা delay bath 3 days ... V<br>**গর্ভ পরবর্তী সেবা (Post partum care)**<br>গর্ভপরবর্তী বিপদ চিহ্নের কথা জানিয়েছে danger signs during Post partum...... W<br>গর্ভপরবর্তী বিপদ চিহ্ন দেখা দিলে হাসপাতালে যাওয়া Care Seeking for Danger S. X	
E13	শেষ যখন আপনি গর্ভবতী ছিলেন, তখন গর্ভকালীন মেডিকেল চেক-আপের সময় কখনও কি আপনার ----------- ? (প্রত্যেকটি বিষয় পড়ে শোনান)<br>ওজন নেয়া হয়েছিল?<br>ব্লাড প্রেসার পরীক্ষা করা হয়েছিল?<br>প্রস্রাব পরীক্ষা করা হয়েছিল?<br>রক্ত পরীক্ষা করা হয়েছিল?<br>আলট্রাসনোগ্রাম করা হয়েছিল?<br>আপনার পেটে হাত দিয়ে পরীক্ষা করা হয়েছিল?<br><br>As part of your antenatal care during this pregnancy, were any of the following done at least once?	হ্যাঁ YES না NO<br>ওজন Weight .... 1........ 2<br>ব্লাড প্রেসার Blood pressure .... 1........ 2<br>প্রস্রাব পরীক্ষা Urine test........ 1........ 2<br>রক্ত পরীক্ষা Blood test........ 1........ 2<br>আলট্রাসনোগ্রাম Ultra sonogram........ 1........ 2<br>পেটে হাত দিয়ে পরীক্ষা Abdominal exam .... 1........ 2	

No	Questions and filters	Options and coding category	Skip
E14	শেষ যখন আপনি গর্ভবতী ছিলেন, তখন আপনাকে দেখতে বা আপনার স্বাস্থ্যের ব্যাপারে উপদেশ দিতে আপনার বাসায় কেউ কি এসেছিলেন?<br>During your most recent pregnacy, did anyone visit you to discuss different issues related to your health?	হ্যাঁ Yes.................................1<br>না No.................................2<br>জানি না/ মনে নাই Don't know/Can't remember.................................9	 →E20<br>→E20
E15	শেষ যখন আপনি গর্ভবতী ছিলেন, তখন আপনাকে দেখতে বা আপনার স্বাস্থ্যের ব্যাপারে উপদেশ দিতে আপনার বাসায় কে এসেছিলেন?<br>*[মহিলাকে জিজ্ঞেস করুন]* আরও কেউ? *[মহিলার নিজে থেকে দেয়া সবগুলো উত্তরই বৃত্তায়িত করুন । উত্তরগুলো পড়ে শুনাবেন না। একাধিক উত্তর হতে পারে।]*<br>*[যদি মহিলার উত্তর সি,এস,বি,এ (Code E) বা MNCS প্রমোটর (Code H) হয়, তাহলে তাদের নাম লিখুন।]*<br><br>**Code :E** নাম Name: ________________________________<br><br>**Code :H** নাম Name: ________________________________<br><br>Who had visited you to discuss issues regarding your health during your most recent pregnancy? *[Do not read out the answers. Ask: Anything else? Circle all the answers]*	**দক্ষ/প্রশিক্ষণ প্রাপ্ত স্বাস্থ্য কর্মী (Medically trained)**<br>পাশ করা ডাক্তার (MBBS doctor).................................A<br>নার্স/ধাত্রী (Nurse/midwife).................................B<br>প্যারামেডিক/মেডিকেল এসিসটেন্ট/সাকমো (Paramedic/MA/SACMO).................................C<br>পরিবার কল্যাণ পরিদর্শক (FWV).................................D<br>সি,এস,বি,এ (CSBA).................................E<br>**অন্যান্য স্বাস্থ্য কর্মী (Other health worker)**<br>স্বাস্থ্য সহকারী/ পরিবার কল্যান সহকারী (HA /FWA).................................F<br>পুষ্টি কর্মী (CNP).................................G<br>MNCS প্রমোটর (MNCS Promoter).................................H<br>অন্যান্য কমিউনিটি স্বাস্থ্য কর্মী - এনজিও কর্মী, স্বেচ্ছাসেবী (Other CHWs, NGO worker, volunteer).................................I<br>**অন্যান্য (Other)**<br>প্রশিক্ষণ প্রাপ্ত টিবিএ (প্রশিক্ষণ প্রাপ্ত ধন্নী, চাউনী, দাই) (TTBA).................................J<br>প্রশিক্ষণহীন টিবিএ (ধন্নী, চাউনী, দাই) TBA(Dai/Dhorni/Chauni).................................K<br>হোমিওপ্যাথ/হোমিওপ্যাথ ঔষধের দোকান (Homeopath/Homeopath drug store).................................L<br>আয়ুর্বেদিক চিকিৎসক / আয়ুর্বেদিক ঔষধের দোকান /হেকিম/কবিরাজ (Ayurved/ Ayurvedic drug store /Hekim/Kabiraj).................................M<br>গ্রাম ডাক্তার (Village doctor).................................N<br>এলোপ্যাথী ঔষধের দোকান (Allopath drug store).................................O<br>ইমাম/ঝাড় ফুক/ওঝা (Spiritual healer).................................P<br>পরিবারের অন্যান্য সদস্য/আত্মীয়/ প্রতিবেশী/বন্ধু Family/relative/Neighbor/friend.................................Q<br>অন্যান্য Others ________________________________..X<br>(নির্দিষ্ট করুন)<br>জানি না/মনে নাই Don't know/can't remember.................................Z	
E16	*[Question E15 দেখুন, code A থেকে I এর যেকোন একটি বা একাধিক বৃত্তায়িত থাকলে কোড/কোডগুলো এখানে লিখুন: _____ , _____, ____ । এবার কোড/কোডগুলো দেখে দেখে মহিলাকে প্রশ্ন করুন ঃ]*<br><br>আপনি বললেন যে, আপনাকে দেখতে বা আপনার স্বাস্থ্যের ব্যাপারে উপদেশ দিতে আপনার বাসায় ----, -----, ----- এসেছিলেন।<br>এখন আমাকে বলুন যে, আপনাকে দেখতে বা আপনার স্বাস্থ্যের ব্যাপারে উপদেশ দিতে আপনার বাসায় উনি/উনারা মোট কতবার এসেছিলেন?<br>উনি/উনাদের মাঝে যে প্রথম আপনাকে দেখতে বা আপনার স্বাস্থ্যের ব্যাপারে উপদেশ দিতে আপনার বাসায় এসেছিলেন, তখন আপনি কতমাসের গর্ভবতী ছিলেন।<br><br>*[মহিলা যদি code A থেকে I এর ভিতর একাধিক স্বাস্থ্যকর্মী মহিলাকে দেখতে বা মহিলার স্বাস্থ্যের ব্যাপারে উপদেশ দিতে মহিলার বাসায় এসে থাকেন, তাহলে তাদের সবার কথাই বলুন এবং তারা মহিলার গর্ভকালীন সময়ে মোট কতবার এসেছিলেন, তা লিখুন এবং এদের ভিতর যে সর্বপ্রথম এসেছিলেন তখন মহিলা কত মাসের গর্ভবতী ছিলেন তা লিখুন।]*<br><br>How many tmes did the person visited you to discuss regarding your health while you were pregnant with <name>? How many months pregnant were you when the person FIRST visited you (in this pregnancy)?	<u>মোট ভিজিটের সংখ্যা:</u><br><br>বার Times.................................|____|____| <br>জানা নাই/মনে নাই DK/can't remember.................................99<br><br><u>১ম ভিজিটের সময়:</u><br><br>মাসের গর্ভবতী Month-pregnant (during 1st visit).................................|____|____| <br>জানা নাই/মনে নাই DK/can't remember.................................98<br><br>প্রযোজ্য নয় Not applicable.................................97	

No	Questions and filters	Options and coding category	Skip
E17	শেষ যখন আপনি গর্ভবতী ছিলেন, তখন আপনাকে দেখতে বা আপনার স্বাস্থ্যের ব্যাপারে উপদেশ দিতে যে আপনার বাসায় এসেছিলেন, তারা আপনার বাড়ীতে এসে গর্ভকালীন সেবা সংক্রান্ত কি কি উপদেশ/পরামর্শ দিয়েছিলেন?<br><br>*[মহিলাকে জিজ্ঞেস করুন]* আরও কিছু? *[মহিলার নিজে থেকে দেয়া সবগুলো উত্তরই বৃত্তায়িত করুন । উত্তরগুলো পড়ে শুনাবেন না। একাধিক উত্তর হতে পারে।]*<br><br>Did the person who visited you provide you any advice regarding care during pregnancy? Can you recall what advice the person gave you regarding care during pregnancy? *[Do not read the answers and record the un-prompted responses.]*	**গর্ভকালীন যত্ন বিষয়ক (Pregnancy care)**<br>অতিরিক্ত ও পুষ্টিকর খাবার খাওয়ার কথা বলেছে take extra and nutritious food.....A<br>বেশী করে পানি খাওয়ার কথা বলেছে Advised to drink more water............B<br>ব্যক্তিগত পরিষ্কার পরিচ্ছন্নতার কথা বলেছে Talked about personal hygience ...C<br>দিনের বেলা ২ ঘন্টা বিশ্রাম, ভারী কাজ না করা 2hr rest/avoid heavy work .....D<br>টিটি নেয়ার কথা বলেছে Advised to take TT vaccination ....E<br>আয়রণ ট্যাবলেট খাওয়ার কথা বলেছে Advised to take Iron tablet...............F<br>গর্ভকালীন বিপদ চিহ্নের কথা জানিয়েছে danger signs during pregnancy.........G<br>গর্ভকালীন বিপদ চিহ্ন দেখা দিলে হাসপাতালে যাওয়া CareSeeking for Danger S ..H<br>**ডেলিভারী কালীন যত্ন বিষয়ক (Delivery care)**<br>জরুরী অবস্থার জন্য টাকা জমানোর কথা Savings for emergency ....I<br>জরুরী অবস্থায় যানবাহনের ব্যবস্থা করা Arrangement of transport for emergency.....J<br>প্রশিক্ষণপ্রাপ্ত সেবাদানকারী দিয়ে ডেলিভারী করানো Delivery by Skilled providers ......K<br>আলো বাতাস পূর্ন স্থানে ডেলিভারী করা delivery at a well-lighted place.........L<br>**নবজাতকের যত্ন বিষয়ক (Neonatal care)**<br>দুই টুকরা কাপড় প্রস্তুত রাখার কথা বলেছে Advised to prepare 2 pieces of cloth .....M<br>নবজাতকের যত্ন নেয়ার জন্য লোক ঠিক করার কথা বলেছে newborn care person N<br>নবজাতকের বিপদ চিহ্ন/লক্ষণ সম্পর্কে জেনেছিলাম danger signs of newborn .O<br>বুকের দুধ খাওয়ানোর কথা Breastfeeding ....P<br>কম ওজনের বাচ্চার যত্নের কথা LBW baby care..................................Q<br>জন্মের পর পরই বাচ্চাকে পরিস্কার করা/শুকিয়ে নেয়ার কথা drying newborn ..R<br>জন্মের পর পরই বাচ্চাকে কাপড় দিয়ে মুড়িয়ে নেয়ার কথা wrapping newborn.S<br>নাভীতে কিছু না দেয়ার কথা Nothing to umbilicus................................T<br>ভারনিক্স/সাদা চামড়া না তোলার কথা Intact vernix ....U<br>প্রসবের ৩ দিন/৭২ ঘন্টা পর প্রথম গোসল করানোর কথা delay bath 3 days ...V<br>**গর্ভ পরবর্তী সেবা (Post partum care)**<br>গর্ভপরবর্তী বিপদ চিহ্নের কথা জানিয়েছে danger signs during Post partum......W<br>গর্ভপরবর্তী বিপদ চিহ্ন দেখা দিলে হাসপাতালে যাওয়া Care Seeking for Danger S..X	
E18	যে আপনার বাসায় এসেছিলেন আপনাকে দেখতে, সে জন্মের পর বাচ্চার যত্ন কিভাবে নিতে হবে সে সম্পর্কে বুঝানোর সময় কি কিছু দেখিয়েছিলেন? দেখিয়ে থাকলে, কি কি দেখিয়েছিল?<br><br>*[প্রথমে উত্তরগুলো পড়ে শোনাবেন না, জিজ্ঞেস করুন ঃ]* আরও কিছু, *[মায়ের নিজে থেকে দেয়া উত্তরগুলো Un-prompted কলামে বৃত্তায়িত করুন।*<br>*এবার মায়ের নিজে থেকে উল্লেখ না করা উত্তরগুলো পড়ে শুনান এবং মায়ের এবারের দেয়া উত্তরগুলো prompted কলামে বৃত্তায়িত করুন*<br><br>Did the person/health worker demonstrate immediate newborn care by showing you things? What did the health worker show you while talking about immediate newborn care?	Un-Prompted .... Prompted<br>পুতুল Doll ....A ....A<br>ছবিওয়ালা কার্ড Card with pictures ....B ....B<br>কাপড় Cloth ....C ....C<br>অন্যান্য Others ....X<br>নির্দিষ্ট করুন (Specify)<br>সে বুঝানোর জন্য কিছু দেখান নি Didn't show me anything.......Y<br>মনে নাই Don't know/cant remember ....Z	
E19	যে আপনার বাসায় এসেছিলেন আপনাকে দেখতে, সে কি আপনাকে কিছু দিয়েছিলেন? উনি আপনাকে কি কি জিনিস দিয়েছিলেন?<br><br>*[প্রথমে উত্তরগুলো পড়ে শোনাবেন না, জিজ্ঞেস করুনঃ]* আরও কিছু, *[মায়ের নিজে থেকে দেয়া উত্তরগুলো Un-prompted কলামে বৃত্তায়িত করুন*<br>*এবার মায়ের নিজে থেকে উল্লেখ না করা উত্তরগুলো পড়ে শুনান এবং মায়ের এবারের দেয়া উত্তরগুলো prompted কলামে বৃত্তায়িত করুন*<br><br>Did the person/health worker give you anything while she visited you at your home during your pregnancy? Can you please name and/or show us those? *[Record all the unprompted and promted answers.]*	Un-Prompted . Prompted<br>রেফারেল স্লিপ Referral Slip ....A ....A<br>ডেলিভারী সম্ভাব্য তারিখের কার্ড Delivery notification card ....B ....B<br>ডেলিভারী কিট Clean delivery kit ....C ....C<br>আয়রন ট্যাবলেট/সিরাপ Iron Tablet/Syrup..........................D ....D<br>ভিজিএফ কার্ড (ভাউচার) VGF Card (Voucher) ....E ....E<br>অন্যান্য Others ____________________________ .X<br>(নির্দিষ্ট করুন)<br>মনে নাই Don't know/cant remember ....Y<br>কিছুই দেয় নি Nothing ....Z	

No	Questions and filters	Options and coding category	Skip
E20	আপনি কি TT টিকা পেয়েছিলেন? আপনার কি TT কার্ড আছে? আমাকে কি আপনার TT কার্ডটি দেখাবেন?<br>Did you recieve TT vaccination? Do you have your TT Card? Can you please let me see that TT card.	হ্যাঁ, এবং TT কার্ডটি দেখিয়েছেন Yes, Card shown.................................. 1<br>হ্যাঁ, তবে TT কার্ড নেই/দেখাতে পারেন নি/খুজে পান নি Yes, but card not shown 2<br>না, TT টিকা পান নি No, TT not received .... 3<br>জানি না/মনে নেই Don't know/can't remember 9	 <br>→ E22<br>→ E22
E21	*[মহিলার টিটি টিকা পাবার ইতিহাস এখানে লিখতে হবে। মহিলার টিটি কার্ড দেখতে চান।*<br>*যদি মহিলা কার্ড দেখাতে পারেন এবং টিটি পাবার তারিখ কার্ড থেকে পাওয়া যায়, তাহলে টিটি পাবার তারিখগুলো তারিখের ঘরে লিখুন।*<br>*যদি মহিলা কার্ড দেখাতে না পারেন, তাহলে মহিলাকে একটি একটি করে প্রথম ৫টি টিটি টিকা পাবার ইতিহাস জানতে চান এবং তা লিখুন।*<br>*টিকা পেয়ে থাকলে 'হ্যাঁ' এবং না পেয়ে থাকলে 'না'-তে বৃত্তায়িত করুন।*<br>*মহিলা টিটি টিকা পেয়েছেন, কিন্তু তারিখ বলতে বা মনে করতে পারছেন না। এমন ক্ষেত্রে 'হ্যাঁ' তে বৃত্তায়িত করুন এবং তারিখের ঘরে 99/99/99 লিখুন।*<br>*একটি টিকা পাবার তারিখ থেকে পরবর্তী টিকাটি পাবার সময়ের পার্থক্য মাস হিসাবে লিখুন।*<br>*টিকাটি সর্বশেষ গর্ভাবস্থায় পেয়ে থাকলে, সেটি টিক চিহ্ন দিয়ে চিহ্নিত করুন।]*<br>*[Record all the TT vaccination history of the woman, either from the TT card or from recalling the event]*	(see grid below)	
E22	একজন মহিলা গর্ভাকালীন সময়ে অসুস্থ না হলেও শরীরে রক্ত হওয়ার জন্য আয়রণ ট্যাবলেট/সিরাপ অথবা ভিটামিন বা অন্য ওষুধ খাওয়ার দরকার আছে কি?<br>Is there any need to consume Iron/Folate, vitamin or other drug during pregnancy even if she is not sick?	হ্যাঁ Yes না No<br>আয়রন/ফলেট ট্যাবলেট/সিরাপ (Iron/Folate)......................... 1 .... 2<br>ভিটামিন (Vitamin)... 1 .... 2<br>অন্যান্য Others ________________________________.... 1 2	
E23	আপনি যখন শেষ গর্ভবতী ছিলেন, তখন আপনি আয়রণ ট্যাবলেট বা আয়রন সিরাপ খেয়েছিলেন কি? *[প্রয়োজনে মহিলাকে আয়রন ট্যাবলেট বা আয়রন সিরাপের বোতল দেখান]*<br>Did you take iron tablets or iron syrup (to increase blood) during this pregnancy? [If necessary then show the tablet or bottle of syrup and then ask]	হ্যাঁ Yes..1<br>না No ....2<br>জানি না/ মনে নাই Don't know/Can't remember 9	 → E26<br>→ E26
E24	আপনি যখন শেষ গর্ভবতী ছিলেন, তখন আপনি মোট কতমাস আয়রন ট্যাবলেট/সিরাপ খেয়েছিলেন?<br>How long did you take the iron tablets/syrup during your last pregnancy?	মাস Months .... |___|___| মনে নাই/ জানি না DK/can't remember..99	

E21 grid:

	মহিলা কি টিটি টিকা পেয়েছেন: না	হ্যাঁ	টিটি পাবার তারিখ	আগেরটি থেকে পার্থক্য	এই গর্ভে পেয়েছেন
TT1	0	1	|__|__|/|__|__|/|__|__|	xxx	☐
TT2	0	1	|__|__|/|__|__|/|__|__|	|__|__|	☐
TT3	0	1	|__|__|/|__|__|/|__|__|	|__|__|	☐
TT4	0	1	|__|__|/|__|__|/|__|__|	|__|__|	☐
TT5	0	1	|__|__|/|__|__|/|__|__|	|__|__|	☐
TT6	0	1	|__|__|/|__|__|/|__|__|	|__|__|	☐
TT7	0	1	|__|__|/|__|__|/|__|__|	|__|__|	☐
TT8	0	1	|__|__|/|__|__|/|__|__|	|__|__|	☐
TT9	0	1	|__|__|/|__|__|/|__|__|	|__|__|	☐
TT10	0	1	|__|__|/|__|__|/|__|__|	|__|__|	☐

No	Questions and filters	Options and coding category	Skip
E25	শেষ যখন আপনি গর্ভবতী ছিলেন, তখন আপনি কোথা থেকে আয়রন ট্যাবলেট পেয়েছিলেন?<br><br>*[মহিলাকে জিজ্ঞেস করুন]* আরও কিছু? *[মহিলার নিজে থেকে দেয়া সবগুলো উত্তরই বৃত্তায়িত করুন । উত্তরগুলো পড়ে শুনাবেন না। একাধিক উত্তর হতে পারে।]*<br><br>Where did you get this iron tablets/syrup? *[Do not read out the answers. Ask: Anything else? Circle all the answers]*	**বাড়ী (Home)**<br>নিজ বাড়ী, স্বামী/শ্বশুড় বাড়ী (Own home, husband/father in laws house).........A<br>বাবার বাড়ী (My natal home) ....B<br>অন্য কোন বাড়ী (Others)________ C<br>(নির্দিষ্ট করুন)<br>**সরকারী স্বাস্থ্য কেন্দ্র (Govt Health center)**<br>মেডিকেল কলেজ হাসপাতাল (Medical College Hospital) ....D<br>জেলা/সদর হাসপাতাল (District /Sadar Hospital) ....E<br>মা ও শিশু স্বাস্থ্য কেন্দ্র (MCWC)........F<br>উপজেলা স্বাস্থ্য কমপ্লেক্স (UHC) ....G<br>ইউনিয়ন স্বাস্থ্য ও পরিবার কল্যাণ কেন্দ্র / সাব সেন্টার/আরডি (FWC/SC/RD) H<br>কমিউনিটি ক্লিনিক (Community clinic)........I<br>সেটেলাইট ক্লিনিক/ ইপিআই কেন্দ্র (Satellite clinic/EPI centre)........J<br>অন্যান্য সরকারী স্বাস্থ্য কেন্দ্র (Other Govt Health facility) ....K<br>**বেসরকারী স্বাস্থ্য কেন্দ্র (Non Govt Health center)**<br>এনজিও হাসপাতাল (NGO hospital)........L<br>এনজিও স্থায়ী স্বাস্থ্য কেন্দ্র (NGO static health centre) ....M<br>এনজিও সেটেলাইট ক্লিনিক (NGO satellite clinic)........N<br>পুষ্টি কেন্দ্র (NNP centre)........O<br>অন্যান্য বেসরকারী স্বাস্থ্য কেন্দ্র (Other NGO Health facility)........P<br>**প্রাইভেট (Private Health sector)**<br>হাসপাতাল/ ক্লিনিক (Hospital/clinic)........Q<br>স্বাস্থ্য কেন্দ্র /ডিসপেনসারী (Health centre/Dispensary) ....R<br>এমবিবিএস ডাক্তারের চেম্বার (MBBS doctor's chamber)........S<br>গ্রাম ডাক্তারের চেম্বার (Village doctor's chamber) ....T<br>প্যারামেডিক/মেডিকেল এসিসটেন্ট/সাকমোর চেম্বার (Paramedic/MA/SACMO chamber) U<br>এলোপ্যাথী ঔষধের দোকান (Allopath drug store) ....V<br>অন্যান্য প্রাইভেট_স্বাস্থ্য কেন্দ্র (Other private Health facility........W<br>অন্যান্য (Others) ________ ..X<br>(নির্দিষ্ট করুন)	
E26	শেষ যখন আপনি গর্ভবতী ছিলেন, তখন (গর্ভবতী থাকাকালীন সময়ে) আপনার এই ডেলিভারীটি কোথায় হবে, সেটি কি ঠিক করে রেখেছিলেন?<br>*[উত্তর 'হ্যাঁ' হলে, জানতে চান,]* কোথায় ডেলিভারী/শিশুটি হবে বলে ঠিক করে রেখেছিলেন?<br>Did you select the place during pregnancy where your child would be deliverd? If yes, ask which place did you select?	বাসায় At Home ....1<br>স্বাস্থ্যকেন্দ্রে Health Center ....2<br>আগে থেকে ঠিক করে রাখি নাই Didn't have any plan 9	 → E33<br>→ E33
E27	আপনার ডেলিভারীটি বাসার কোন স্থানে বা কোন ঘরে হবে তা কি ঠিক করে রেখেছিলেন? স্থানটি/ঘরটি কি ধরনের? স্থানটি/ঘরটিতে কি আলো বাতাস ছিল?<br>Delivery sould be conducted in a well lighted and airy place. Did you select such place beforehand?	হ্যাঁ, স্থান/ঘরটি আলো বাতাস পূর্ণ Yes, room was well lighted........1<br>না, আলো বাতাস পূর্ণ না No, wasn't well lighted........2<br>না, কোন স্থান/ঘর আগে থেকে ঠিক করা হয় নাই No, wasn't pre-fixed ....8<br>জানি না/ মনে নাই Don't know/Can't remember 9	
E28	শেষ যখন আপনি গর্ভবতী ছিলেন, তখন (গর্ভবতী থাকাকালীন সময়ে) আপনার ডেলিভারীতে সাহায্য করার জন্য বা ডেলিভারীটি করানোর জন্য আপনারা কাউকে কি ঠিক করে রেখেছিলেন?<br>During your most recent pregnancy, did you or your family select someone to help you during delivery?	হ্যাঁ Yes........1<br>না No ....2<br>জানি না/ মনে নাই Don't know/Can't remember 9	 → E30<br>→ E30

No	Questions and filters	Options and coding category	Skip
E29	ডেলিভারীতে সাহায্য করার জন্য বা ডেলিভারীটি করানোর জন্য আপনারা কাকে ঠিক করে রেখেছিলেন?<br><br>*[কেবলমাত্র একটি উত্তর হবে]*<br><br>*[যদি মহিলার উত্তর সি,এস,বি,এ (Code 15) বা MNCS প্রমোটর (Code 23) হয়, তাহলে তাদের নাম লিখুন।]*<br><br>**Code :15** নাম Name: ______________________________<br><br>**Code :23** নাম Name: ______________________________<br><br>Whom did you or your family select to help you during the delivery? Ask for the name of the person who was principally selected to assist delivery and record the name	**দক্ষ/প্রশিক্ষণ প্রাপ্ত স্বাস্থ্য কর্মী (Medically trained)**<br>পাশ করা ডাক্তার (MBBS doctor) .... 11<br>নার্স/ধাত্রী (Nurse/midwife) .... 12<br>প্যারামেডিক/মেডিকেল এসিসটেন্ট/সাকমো (Paramedic/MA/SACMO) .... 13<br>পরিবার কল্যাণ পরিদর্শক (FWV) .... 14<br>সি,এস,বি,এ (CSBA) .... 15<br>**অন্যান্য স্বাস্থ্য কর্মী (Other health worker)**<br>স্বাস্থ্য সহকারী/ পরিবার কল্যান সহকারী (HA /FWA) .... 21<br>পুষ্টি কর্মী (CNP) .... 22<br>MNCS প্রমোটর (MNCS Promoter) .... 23<br>অন্যান্য কমিউনিটি স্বাস্থ্য কর্মী - এনজিও কর্মী, স্বেচ্ছাসেবী (Other CHWs, NGO worker, volunteer) .... 24<br>**অন্যান্য (Other)**<br>প্রশিক্ষণ প্রাপ্ত টিবিএ (প্রশিক্ষণ প্রাপ্ত ধন্নী, চাউনী, দাই) (TTBA) .... 31<br>প্রশিক্ষণহীন টিবিএ (ধন্নী, চাউনী, দাই) TBA(Dai/Dhorni/Chauni) .... 32<br>হোমিওপ্যাথ/হোমিওপ্যাথ ঔষধের দোকান (Homeopath/Homeopath drug store) .... 33<br>আয়ুর্বেদিক চিকিৎসক / আয়ুর্বেদিক ঔষধের দোকান /হেকিম/কবিরাজ (Ayurved/ Ayurvedic drug store /Hekim/Kabiraj) .... 34<br>গ্রাম ডাক্তার (Village doctor) .... 35<br>এলোপ্যাথী ঔষধের দোকান (Allopath drug store) .... 36<br>ইমাম/ঝাড় ফুক/ওঝা (Spiritual healer) .... 37<br>পরিবারের অন্যান্য সদস্য/আত্মীয়/প্রতিবেশী/বন্ধু Family/relative/Neighbor/friend .... 38<br>অন্যান্য Others ______________________ .. 39<br>(নির্দিষ্ট করুন)<br>জানি না/মনে নাই Don't know/can't remember 99	
E30	ডেলিভারীর পর পরই বাচ্চার যত্ন করার জন্য আপনি শেষবার গর্ভবতী থাকা অবস্থাতেই কি কাউকে ঠিক করে রেখেছিলেন কি?<br>During your most recent pregnancy, did you or your family selects a person to help you with care for the newborn after delivery?	হ্যাঁ Yes .... 1<br>না No .... 2<br>জানি না/ মনে নাই Don’t know/Can’t remember 9	 → E32<br>→ E32
E31	ডেলিভারীর পর পরই বাচ্চার যত্ন করার জন্য আপনারা কাকে ঠিক করে রেখেছিলেন?<br><br>*[কেবলমাত্র একটি উত্তর হবে]*<br><br>*[যদি মহিলার উত্তর সি,এস,বি,এ (Code 15) বা MNCS প্রমোটর (Code 23) হয়, তাহলে তাদের নাম লিখুন।]*<br><br>**Code :15** নাম Name: ______________________________<br><br>**Code :23** নাম Name: ______________________________<br><br>Who did you or your family select to help you with care for the newborn after delivery? *Ask for the name of the person who was principally selected to assist delivery and record the name. Record his/her code from the list]*	**দক্ষ/প্রশিক্ষণ প্রাপ্ত স্বাস্থ্য কর্মী (Medically trained)**<br>পাশ করা ডাক্তার (MBBS doctor) .... 11<br>নার্স/ধাত্রী (Nurse/midwife) .... 12<br>প্যারামেডিক/মেডিকেল এসিসটেন্ট/সাকমো (Paramedic/MA/SACMO) .... 13<br>পরিবার কল্যাণ পরিদর্শক (FWV) .... 14<br>সি,এস,বি,এ (CSBA) .... 15<br>**অন্যান্য স্বাস্থ্য কর্মী (Other health worker)**<br>স্বাস্থ্য সহকারী/ পরিবার কল্যান সহকারী (HA /FWA) .... 21<br>পুষ্টি কর্মী (CNP) .... 22<br>MNCS প্রমোটর (MNCS Promoter) .... 23<br>অন্যান্য কমিউনিটি স্বাস্থ্য কর্মী - এনজিও কর্মী, স্বেচ্ছাসেবী (Other CHWs, NGO worker, volunteer) .... 24<br>**অন্যান্য (Other)**<br>প্রশিক্ষণ প্রাপ্ত টিবিএ (প্রশিক্ষণ প্রাপ্ত ধন্নী, চাউনী, দাই) (TTBA) .... 31<br>প্রশিক্ষণহীন টিবিএ (ধন্নী, চাউনী, দাই) TBA(Dai/Dhorni/Chauni) .... 32<br>হোমিওপ্যাথ/হোমিওপ্যাথ ঔষধের দোকান (Homeopath/Homeopath drug store) .... 33<br>আয়ুর্বেদিক চিকিৎসক / আয়ুর্বেদিক ঔষধের দোকান /হেকিম/কবিরাজ (Ayurved/ Ayurvedic drug store /Hekim/Kabiraj) .... 34<br>গ্রাম ডাক্তার (Village doctor) .... 35<br>এলোপ্যাথী ঔষধের দোকান (Allopath drug store) .... 36<br>ইমাম/ঝাড় ফুক/ওঝা (Spiritual healer) .... 37<br>পরিবারের অন্যান্য সদস্য/আত্মীয়/প্রতিবেশী/বন্ধু Family/relative/Neighbor/friend .... 38<br>অন্যান্য Others ______________________ .. 39<br>(নির্দিষ্ট করুন)<br>জানি না/মনে নাই Don't know/can't remember 99	

No	Questions and filters	Options and coding category	Skip
E32	ডেলিভারীর সময় ব্যবহারের জন্য শেষবার গর্ভবতী থাকা অবস্থাতেই ডেলিভারী কিট ক্রয় বা সংগ্রহ করে রেখেছিলেন কি?<br>Did you buy or collect delivery kit for uing during delivery?	হ্যাঁ Yes....1<br>না No ....2<br>জানি না/ মনে নাই Don't know/Can't remember 9	
E33	ডেলিভারীর সময় যদি কোন অসুবিধা হয়, তাহলে যেন তাড়াতাড়ি হাসপাতাল বা স্বাস্থ্যকেন্দ্রে যাওয়া যায়, সে জন্য আগে থেকেই কি যানবাহনের ব্যবস্থা করে রেখেছিলেন?<br>Did you arrange emergency transport before delivery in case complication during delivery?	হ্যাঁ Yes....1<br>না No ....2<br>জানি না/ মনে নাই Don't know/Can't remember 9	
E34	ডেলিভারীর সময়টায় টাকা পয়সা লাগতে পারে, তাই আগে থেকেই টাকা পয়সা জমা করে রেখেছিলেন কি?<br>Did you save money for emergency need during delivery?	হ্যাঁ Yes....1<br>না No ....2<br>জানি না/ মনে নাই Don't know/Can't remember 9	
E35	ডেলিভারীর পর পর শিশুটিকে মুড়ানো মোছানোর জন্য কমপক্ষে দুই টুকরা কাপড় কি জোগাড় করে রেখেছিলেন?<br>Did you arrange two pieces of cloth for drying and wrapping the baby?	হ্যাঁ Yes....1<br>না No ....2<br>জানি না/ মনে নাই Don't know/Can't remember 9	
E36	শেষ যখন আপনি গর্ভবতী ছিলেন, তখন কি আপনার কোন সমস্যা/জটিলতা হয়েছিল?<br>During your most recent pregnancy, did you develop any problem/complication?	হ্যাঁ Yes....1<br>না No ....2<br>জানি না/ মনে নাই Don't know/Can't remember 9	 → F01<br>→ F01
E37	শেষ যখন আপনি গর্ভবতী ছিলেন, তখন আপনার কি ধরনের সমস্যা/জটিলতা হয়েছিল?<br>*[মহিলাকে জিজ্ঞেস করুন]* আরও কোন সমস্যা/জটিলতা? *[মহিলার নিজে থেকে দেয়া সবগুলো উত্তরই বৃত্তায়িত করুন । উত্তরগুলো পড়ে শুনাবেন না । একাধিক উত্তর হতে পারে ।]*<br>Please tell me what was that problem/complication? *[Do not read out the answers. Ask: Anything else? Circle all the answers]*	তীব্র মাথা ব্যথা Severe Headache ....A<br>চোখে ঝাপসা দেখা Blurred Vision ....B<br>গর্ভের বাচ্চার নড়াচড়া কমে যাওয়া/বন্ধ হওয়া Fetal movement reduced/absent ....C<br>উচ্চ রক্তচাপ High Blood Pressure ....D<br>মুখমন্ডলে পানি আসা/ফুলে যাওয়া Face edema/swelling ....E<br>হাতে পানি আসা/ফুলে যাওয় Hand edema/swelling ....F<br>খিচুনী/ফিট Convulsions/fits ....G<br>যোনীপথে অতিরিক্ত রক্তস্রাব Excessive Vaginal Bleeding ....H<br>তলপেটে তীব্র ব্যথা Severe abdominal pain ....I<br>পায়ে পানি আসা Oedema of the legs ....J<br>তীব্র জ্বর Hight Fever ....K<br>সময় পূর্ন হওয়ার আগে পানি ভাঙ্গা Premature rupture of membrane ....L<br>অচেতন হওয়া/জ্ঞান হারিয়ে ফেলা Loss of consciousness ....M<br>শ্বাস নিতে কষ্ট হওয়া Difficulty breathing....N<br>প্রচন্ড দুর্বলতা Severe weakness....O<br>অন্যান্য Others ______ X<br>নির্দিষ্ট করুন	
E38	[E37 দেখুন এবং সঠিক কোড বৃত্তায়িত করুন ।]<br>Interviewer: Check E37<br>Is/Are there any complication/s mentioned by the women?	এক বা একাধিক কোড বৃত্তায়িত One or more codes is/are circled ....1<br>কোন কোড বৃত্তায়িত হয়নি No code is circled 2	 → F01
E39	আপনার মতে সমস্যা/ জটিলতা টি কি খুব মারাত্মক নাকি মোটমুটি নাকি সামান্য ছিল?<br>From your opinion, was this problem/complication severe or mild to moderate?	সামান্য ছিল Mild ....1<br>মোটামুটি ছিল Moderate ....2<br>মারাত্মক ছিল Severe....3<br>অন্যান্য Other : ______ ...7<br>জানেন না Doesn't know 9	
E40	শেষ যখন আপনি গর্ভবতী ছিলেন, তখন সমস্যা/জটিলতার জন্য আপনি কি কাউকে দেখিয়েছিলেন বা কারও সাহায্য নিয়েছিলেন?<br>Did you seek any sort of treatment for this problem/complication?	হ্যাঁ Yes....1<br>না No ....2<br>জানি না/ মনে নাই Don't know/Can't remember 9	 → E46<br>→ F01

No	Questions and filters	Options and coding category	Skip
E41	এই সমস্যা/জটিলতার জন্য আপনি কাকে দেখিয়েছিলেন বা কার সাহায্য নিয়েছিলেন?<br><br>*[মহিলাকে জিজ্ঞেস করুন]* আরও কেউ? *[মহিলার নিজে থেকে দেয়া সবগুলো উত্তরই বৃত্তায়িত করুন । উত্তরগুলো পড়ে শুনাবেন না। একাধিক উত্তর হতে পারে।]*<br><br>*[যদি মহিলার উত্তর সি,এস,বি,এ (Code E) বা MNCS প্রমোটর (Code H) হয়, তাহলে তাদের নাম লিখুন।]*<br><br>**Code :E** নাম Name: ______<br><br>**Code :H** নাম Name: ______<br><br>From whom did you seek treatment for this problem/complication? Do not read out the answers. Ask: Anything else? Circle all the answers	**দক্ষ/প্রশিক্ষণ প্রাপ্ত স্বাস্থ্য কর্মী (Medically trained)**<br>পাশ করা ডাক্তার (MBBS doctor) .... A<br>নার্স/ধাত্রী (Nurse/midwife) .... B<br>প্যারামেডিক/মেডিকেল এসিসটেন্ট/সাকমো (Paramedic/MA/SACMO) .... C<br>পরিবার কল্যাণ পরিদর্শক (FWV) .... D<br>সি,এস,বি,এ (CSBA) .... E<br>**অন্যান্য স্বাস্থ্য কর্মী (Other health worker)**<br>স্বাস্থ্য সহকারী/ পরিবার কল্যান সহকারী (HA /FWA) .... F<br>পুষ্টি কর্মী (CNP) .... G<br>MNCS প্রমোটর (MNCS Promoter) .... H<br>অন্যান্য কমিউনিটি স্বাস্থ্য কর্মী - এনজিও কর্মী, স্বেচ্ছাসেবী (Other CHWs, NGO worker, volunteer) .... I<br>**অন্যান্য (Other)**<br>প্রশিক্ষণ প্রাপ্ত টিবিএ (প্রশিক্ষণ প্রাপ্ত ধন্নী, চাউনী, দাই) (TTBA) .... J<br>প্রশিক্ষণহীন টিবিএ (ধন্নী, চাউনী, দাই) TBA(Dai/Dhorni/Chauni) .... K<br>হোমিওপ্যাথ/হোমিওপ্যাথ ঔষধের দোকান (Homeopath/Homeopath drug store) .... L<br>আয়ুর্বেদিক চিকিৎসক / আয়ুর্বেদিক ঔষধের দোকান /হেকিম/কবিরাজ (Ayurved/ Ayurvedic drug store /Hekim/Kabiraj) .... M<br>গ্রাম ডাক্তার (Village doctor) .... N<br>এলোপ্যাথী ঔষধের দোকান (Allopath drug store) .... O<br>ইমাম/ঝাড় ফুক/ওঝা (Spiritual healer) .... P<br>পরিবারের অন্যান্য সদস্য/আত্মীয়/ প্রতিবেশী/বন্ধু Family/relative/Neighbor/friend .... Q<br>অন্যান্য Others ______ .. X<br>(নির্দিষ্ট করুন)<br>জানি না/মনে নাই Don't know/can't remember .... Z	
E42	এই সমস্যা/জটিলতার জন্য আপনি কোথায় দেখিয়েছিলেন বা সেবা কোথায় পেয়েছিলেন?<br><br>*[মহিলাকে জিজ্ঞেস করুন ,]* আরও কোথাও? *[মহিলার নিজে থেকে দেয়া সবগুলো উত্তরই বৃত্তায়িত করুন । উত্তরগুলো পড়ে শুনাবেন না। একাধিক উত্তর হতে পারে।]*<br><br>*[মহিলা যেখান থেকে সেবা পেয়েছেন, সেই স্বাস্থ্যকেন্দ্রের নাম লিখুন। যদি একাধিক জায়গা থেকে সেবা নিয়ে থাকেন, তাহলে সবগুলো জায়গারই নাম এবং কোড লিখুন।]*<br><br>Code :|____| নাম Name: ______<br><br>Code :|____| নাম Name: ______<br><br>Code :|____| নাম Name: ______<br><br>Where did you go to seek care for this problem/complication? Do not read out the answers. Ask: Anything else? Circle all the answers	**বাড়ী (Home)**<br>নিজ বাড়ী, স্বামী/শ্বশুড় বাড়ী (Own home, husband/father in laws house) .... A<br>বাবার বাড়ী (My natal home) .... B<br>অন্য কোন বাড়ী (Others)______ C<br>(নির্দিষ্ট করুন)<br>**সরকারী স্বাস্থ্য কেন্দ্র (Govt Health center)**<br>মেডিকেল কলেজ হাসপাতাল (Medical College Hospital) .... D<br>জেলা/সদর হাসপাতাল (District /Sadar Hospital) .... E<br>মা ও শিশু স্বাস্থ্য কেন্দ্র (MCWC) .... F<br>উপজেলা স্বাস্থ্য কমপ্লেক্স (UHC) .... G<br>ইউনিয়ন স্বাস্থ্য ও পরিবার কল্যাণ কেন্দ্র / সাব সেন্টার/আরডি (FWC/SC/RD) H<br>কমিউনিটি ক্লিনিক (Community clinic) .... I<br>সেটেলাইট ক্লিনিক/ ইপিআই কেন্দ্র (Satellite clinic/EPI centre) .... J<br>অন্যান্য সরকারী স্বাস্থ্য কেন্দ্র (Other Govt Health facility) .... K<br>**বেসরকারী স্বাস্থ্য কেন্দ্র (Non Govt Health center)**<br>এনজিও হাসপাতাল (NGO hospital) .... L<br>এনজিও স্থায়ী স্বাস্থ্য কেন্দ্র (NGO static health centre) .... M<br>এনজিও সেটেলাইট ক্লিনিক (NGO satellite clinic) .... N<br>পুষ্টি কেন্দ্র (NNP centre) .... O<br>অন্যান্য বেসরকারী স্বাস্থ্য কেন্দ্র (Other NGO Health facility) .... P<br>**প্রাইভেট (Private Health sector)**<br>হাসপাতাল/ ক্লিনিক (Hospital/clinic) .... Q<br>স্বাস্থ্য কেন্দ্র /ডিসপেনসারী (Health centre/Dispensary) .... R<br>এমবিবিএস ডাক্তারের চেম্বার (MBBS doctor's chamber) .... S<br>গ্রাম ডাক্তারের চেম্বার (Village doctor's chamber) .... T<br>প্যারামেডিক/মেডিকেল এসিসটেন্ট/সাকমোর চেম্বার (Paramedic/MA/SACMO chamber) U<br>এলোপ্যাথী ঔষধের দোকান (Allopath drug store) .... V<br>অন্যান্য প্রাইভেট_স্বাস্থ্য কেন্দ্র (Other private Health facility .... W<br>অন্যান্য (Others) ______ .. X<br>(নির্দিষ্ট করুন)	

No	Questions and filters	Options and coding category	Skip
E43	এই সমস্যা/জটিলতার জন্য কাউকে দেখানোর ব্যাপারে কেউ কি আপনাকে পরামর্শ দিয়েছিল ?<br>Did anyone advise you or refer you to seek care for your complication?	হ্যাঁ Yes........1<br>না No........2<br>জানি না/ মনে নাই Don't know/Can't remember........9	 → F01<br>→ F01
E44	এই সমস্যা/জটিলতার জন্য কাউকে দেখানোর ব্যাপারে কে আপনাকে পরামর্শ দিয়েছিল?<br><br>*[মহিলাকে জিজ্ঞেস করুন]* আরও কেউও? *[মহিলার নিজে থেকে দেয়া সবগুলো উত্তরই বৃত্তায়িত করুন । উত্তরগুলো পড়ে শুনাবেন না। একাধিক উত্তর হতে পারে।]*<br><br>*[যদি মহিলার উত্তর সি,এস,বি,এ (Code E) বা MNCS প্রমোটর (Code H) হয়, তাহলে তাদের নাম লিখুন।]*<br><br>**Code :E** নাম Name: ________________<br><br>**Code :H** নাম Name: ________________<br><br>Who had advised you or refered you to seek care for your complication? Do not read out the answers. Ask: Anything else? Circle all the answers	**দক্ষ/প্রশিক্ষণ প্রাপ্ত স্বাস্থ্য কর্মী (Medically trained)**<br>পাশ করা ডাক্তার (MBBS doctor)........A<br>নার্স/ধাত্রী (Nurse/midwife)........B<br>প্যারামেডিক/মেডিকেল এসিসটেন্ট/সাকমো (Paramedic/MA/SACMO)........C<br>পরিবার কল্যাণ পরিদর্শক (FWV)........D<br>সি,এস,বি,এ (CSBA)........E<br>**অন্যান্য স্বাস্থ্য কর্মী (Other health worker)**<br>স্বাস্থ্য সহকারী/ পরিবার কল্যান সহকারী (HA /FWA)........F<br>পুষ্টি কর্মী (CNP)........G<br>MNCS প্রমোটর (MNCS Promoter)........H<br>অন্যান্য কমিউনিটি স্বাস্থ্য কর্মী - এনজিও কর্মী, স্বেচ্ছাসেবী (Other CHWs, NGO worker, volunteer)........I<br>**অন্যান্য (Other)**<br>প্রশিক্ষণ প্রাপ্ত টিবিএ (প্রশিক্ষণ প্রাপ্ত ধন্নী, চাউনী, দাই) (TTBA)........J<br>প্রশিক্ষণহীন টিবিএ (ধন্নী, চাউনী, দাই) TBA(Dai/Dhorni/Chauni)........K<br>হোমিওপ্যাথ/হোমিওপ্যাথ ঔষধের দোকান (Homeopath/Homeopath drug store)........L<br>আয়ুর্বেদিক চিকিৎসক / আয়ুর্বেদিক ঔষধের দোকান /হেকিম/কবিরাজ (Ayurved/ Ayurvedic drug store /Hekim/Kabiraj)........M<br>গ্রাম ডাক্তার (Village doctor)........N<br>এলোপ্যাথী ঔষধের দোকান (Allopath drug store)........O<br>ইমাম/ঝাড় ফুক/ওঝা (Spiritual healer)........P<br>পরিবারের অন্যান্য সদস্য/আত্মীয়/ প্রতিবেশী/বন্ধু Family/relative/Neighbor/friend........Q<br>অন্যান্য Others ________________..X<br>(নির্দিষ্ট করুন)<br>জানি না/মনে নাই Don't know/can't remember........Z	
E45	এই সমস্যা/জটিলতার জন্য কাউকে দেখানোর ব্যাপারে যিনি আপনাকে পরামর্শ করেছিলেন, তিনি কি আপনাকে কোন কাগজ (রেফারেল স্লিপ) দিয়েছিলেন? (রেফারেল স্লিপের একটি নমুনা দেখান)<br>Did the person who had referred you issue you a referral slip to show it to the health facility/carer? (show the referral slip to the respondent)	হ্যাঁ Yes........1<br>না No........2<br>জানি না/ মনে নাই Don't know/Can't remember........9	→ F01<br>→ F01<br>→ F01
E46	আপনি কেন এই সমস্যা/জটিলতার জন্য কাউকে দেখান নি?<br><br>*[মহিলাকে জিজ্ঞেস করুন ,]* আরও কোন কারন? *[মহিলার নিজে থেকে দেয়া সবগুলো উত্তরই বৃত্তায়িত করুন । উত্তরগুলো পড়ে শুনাবেন না। একাধিক উত্তর হতে পারে।]*<br><br>Why did you not seek care for this complication? Do not read out the answers. Ask: Anything else? Circle all the answers	সেবার প্রয়োজন আছে বলে মনে হয় নি/সেবার প্রয়োজন নেই Didn't think it was necessary to seek care........A<br>জানতাম না কোথায় যেতে হবে Not known where to go........B<br>অনেক খরচ/ টাকা পয়সা ছিল না Too costly/ Lack of money........C<br>স্বাস্থ্য কেন্দ্র বাসা হতে অনেক দুরে Too far from house........D<br>যানবাহনের সমস্যা Transport problem........E<br>সাথে যাবার মত কেউ ছিল না No one accompanied........F<br>স্বাস্থ্যকেন্দ্রে যাবার মত সময় ছিল না Not enough time to go........G<br>পরিবার আমাকে যেতে দেন নি Family didn't allow me to go........H<br>ধর্মে মানা/বাধা Religeous bar........I<br>স্বাস্থ্যকেন্দ্রের সেবাদানের সময় সীমা সুবিধাজনক নয় Service hr inconvenient...J<br>স্বাস্থ্যকেন্দ্র বন্ধ ছিল/কোন স্বাস্থ্যকর্মী ছিলেন না HF found closed/nobody there.....K<br>স্বাস্থ্যকেন্দ্রে সেবা অনুন্নত মানের Poor quality of services at facility........L<br>স্বাস্থ্যকেন্দ্রে নিম্নমানের এবং অদক্ষ সেবাপ্রদানকারী Poor quality & staffs at HF ..M<br>স্বাস্থ্যকেন্দ্রে পর্দার অভাব Lack of privacy........N<br>স্বাস্থ্যকেন্দ্রের সেবাদানকারীদের ব্যবহার খারাপ Unpleasant behavior at center ...O<br>স্বাস্থ্যকেন্দ্রের অনেকক্ষন বসে থাকতে হয় সেবা পাবার জন্য Long queue at HF .P<br>স্বাস্থ্যকেন্দ্রের ঔষধ পত্র পাওয়া যায়না Inadequate drugs at the health center ....Q<br>অন্যান্য Others ________________..X	

Section F: Delivery Care

This Section contains some information care of the mother during delivery

No	Questions and filters	Options and coding category	Skip
F01	*[১ লা জুন 2011 বা এর পর হওয়া সর্বশেষ ডেলিভারীর সময় মহিলার আত্মীয় স্বজনদের ভিতর যারা উপস্থিত ছিলেন, তাদেরকে মহিলাকে উত্তরে সাহায্য করার জন্য এই অংশে ও পরের অংশে ডাকুন। তাদের ভিতর এখন এই ইন্টারভিউয়ের সময় উপস্থিত ছিলেন যারা, তারা কারা? তাদের সাথে মহিলার সম্পর্ক কি?]*<br><br>*[Ask the mother to call the people who were present during the time of delivery. These persons will help the mother to answer the questions in this section]*	মা Mother .... A<br>শ্বাশুড়ী Mother-in-law .... B<br>বোন/ননদ/জা Sister .... C<br>চাচী/মামী/খালা/ফুফুAunt .... D<br>দাদী/নানী Grandmother .... E<br>ভাগনি/ভাতিজী Niece .... F<br>অন্য কোন মহিলা আত্মীয় other female relative ____________ .. X<br>ডেলিভারীর সময় কোন আপনজন উপস্থিত ছিলেন না No relatives at delivery . Y<br>যারা ডেলিভারীর সময় ছিলেন, তারা এখন এখানে নেই None present now ..... Z	
F02	ডেলিভারী সাধারনত: কাকে দিয়ে করানো উচিত বলে আপনি মনে করেন ?<br><br>*[কেবলমাত্র একটি উত্তর হবে]*<br><br>Whom do you think should assist during delivery?	**দক্ষ/প্রশিক্ষণ প্রাপ্ত স্বাস্থ্য কর্মী (Medically trained)**<br>পাশ করা ডাক্তার (MBBS doctor) .... 11<br>নার্স/ধাত্রী (Nurse/midwife) .... 12<br>প্যারামেডিক/মেডিকেল এসিসটেন্ট/সাকমো (Paramedic/MA/SACMO) .... 13<br>পরিবার কল্যাণ পরিদর্শক (FWV) .... 14<br>সি,এস,বি,এ (CSBA) .... 15<br>**অন্যান্য স্বাস্থ্য কর্মী (Other health worker)**<br>স্বাস্থ্য সহকারী/ পরিবার কল্যান সহকারী (HA /FWA) .... 21<br>পুষ্টি কর্মী (CNP) .... 22<br>MNCS প্রমোটর (MNCS Promoter) .... 23<br>অন্যান্য কমিউনিটি স্বাস্থ্য কর্মী - এনজিও কর্মী, স্বেচ্ছাসেবী (Other CHWs, NGO worker, volunteer) .... 24<br>**অন্যান্য (Other)**<br>প্রশিক্ষণ প্রাপ্ত টিবিএ (প্রশিক্ষণ প্রাপ্ত ধন্নী, চাউনী, দাই) (TTBA) .... 31<br>প্রশিক্ষণহীন টিবিএ (ধন্নী, চাউনী, দাই) TBA(Dai/Dhorni/Chauni) .... 32<br>হোমিওপ্যাথ/হোমিওপ্যাথ ঔষধের দোকান (Homeopath/Homeopath drug store) .... 33<br>আয়ুর্বেদিক চিকিৎসক / আয়ুর্বেদিক ঔষধের দোকান /হেকিম/কবিরাজ (Ayurved/ Ayurvedic drug store /Hekim/Kabiraj) .... 34<br>গ্রাম ডাক্তার (Village doctor) .... 35<br>এলোপ্যাথী ঔষধের দোকান (Allopath drug store) .... 36<br>ইমাম/ঝাড় ফুক/ওঝা (Spiritual healer) .... 37<br>পরিবারের অন্যান্য সদস্য/আত্মীয়/প্রতিবেশী/বন্ধু Family/relative/Neighbor/friend ... 38<br>অন্যান্য Others ____________ .. 39<br>(নির্দিষ্ট করুন)<br>জানি না/মনে নাই Don't know/can't remember 99	→F04
F03	*[যদি মা উপরের প্রশ্নের একটি উত্তর (জানি না ছাড়া অন্য কোন উত্তর) দিয়ে থাকেন, তাহলে মাকে জিজ্ঞেস করুন যে,]*<br><br>আপনি যে বললেন, ডেলিভারীটি ------কে দিয়ে করানো উচিত - এই তথ্য আপনি কোথা থেকে বা কার কাছ থেকে জেনেছেন?<br><br>*[মহিলাকে জিজ্ঞেস করুন]* আরও কেউ? *[মহিলার নিজে থেকে দেয়া সবগুলো উত্তরই বৃত্তায়িত করুন । উত্তরগুলো পড়ে শুনাবেন না। একাধিক উত্তর হতে পারে।]*<br><br>If mother mentions one or more of the above, then ask. from where /whom did you get this knowledge/information?	ডাক্তার/নার্স/ধাত্রী/প্যারামেডিক (Doctor/ Nurse/Midwife/ Paramedics) .... A<br>স্বাস্থ্য কেন্দ্র/হাসপাতাল (Health facility/ Clinic/ Hospital) .... B<br>কমিউনিটি স্বাস্থ্য কর্মী - স্বাস্থ্য সহকারী/পরিবার কল্যান সহকারী, পুষ্টি কর্মী, এনজিও কর্মী, MNCS প্রমোটর, স্বেচ্ছাসেবী (CHWs -HAS, FWAs, CNPs, MNCS promoter,NGO worker, volunteer) .... C<br>কমিউনিটি গ্রুপ মিটিং / মিটিং /সভা থেকে (Community meeting) .... D<br>রেডিও/টিভি (Radio /TV) .... E<br>সংবাদপত্র/খবরের কাগজ/বইপত্র (Newspaper/Books) .... F<br>পোষ্টার / কোন ছাপানো কাগজ (Poster/Leaflet) .... G<br>পরিবারের অন্যান্য সদস্য/আত্মীয়/প্রতিবেশী/বন্ধু Family/relatives/Neighbor/friend ..... H<br>অন্যান্য Others____________ ... X<br>(নির্দিষ্ট করুন)<br>মনে নাই Can't remember .... Y	

No	Questions and filters	Options and coding category	Skip
F04	আপনার সর্বশেষ ডেলিভারীটি কোথায় হয়েছিল?<br><br>[কেবলমাত্র একটি উত্তর হবে]<br><br>*[মহিলার ডেলিভারীটি যেখানে হয়েছিল, সেটি কোন স্বাস্থ্যকেন্দ্র হয়ে থাকলে, তার নাম লিখুন।]*<br><br>Code :|____|____| <br>নাম Name: ______________________________<br><br>Where did the birth/delivery of your most recent pregnancy take place?	**বাড়ী (Home)**<br>নিজ বাড়ী, স্বামী/শশুড় বাড়ী (Own home, husband/father in laws h)............11<br>বাবার বাড়ী (My natal home) ....12<br>অন্য কোন বাড়ী (Others)________________________________..13<br>(নির্দিষ্ট করুন)<br>**সরকারী স্বাস্থ্য কেন্দ্র (Govt Health center)**<br>মেডিকেল কলেজ হাসপাতাল (Medical College Hospital) ....21<br>জেলা/সদর হাসপাতাল (District /Sadar Hospital) ....22<br>মা ও শিশু স্বাস্থ্য কেন্দ্র (MCWC)...23<br>উপজেলা স্বাস্থ্য কমপ্লেক্স (UHC) ....24<br>ইউনিয়ন স্বাস্থ্য ও পরিবার কল্যাণ কেন্দ্র / সাব সেন্টার/আরডি (FWC/SC/RD) 25<br>কমিউনিটি ক্লিনিক (Community clinic)...26<br>সেটেলাইট ক্লিনিক/ ইপিআই কেন্দ্র (Satellite clinic/EPI centre)..................27<br>অন্যান্য সরকারী স্বাস্থ্য কেন্দ্র (Other Govt Health facility) ....28<br>**বেসরকারী স্বাস্থ্য কেন্দ্র (Non Govt Health center)**<br>এনজিও হাসপাতাল (NGO hospital)...31<br>এনজিও স্থায়ী স্বাস্থ্য কেন্দ্র (NGO static health centre) ....32<br>এনজিও সেটেলাইট ক্লিনিক (NGO satellite clinic)................................33<br>পুষ্টি কেন্দ্র (NNP centre)...34<br>অন্যান্য বেসরকারী স্বাস্থ্য কেন্দ্র (Other NGO Health facility).....................35<br>**প্রাইভেট (Private Health sector)**<br>হাসপাতাল/ ক্লিনিক (Hospital/clinic)...41<br>স্বাস্থ্য কেন্দ্র /ডিসপেনসারী (Health centre/Dispensary) ....42<br>এমবিবিএস ডাক্তারের চেম্বার (MBBS doctor's chamber).........................43<br>গ্রাম ডাক্তারের চেম্বার (Village doctor's chamber) ....44<br>প্যারামেডিক/মেডিকেল এসিসটেন্ট/সাকমোর (Paramedic/MA/SACMO chamber) ...45<br>এলোপ্যাথী ঔষধের দোকান (Allopath drug store) ....46<br>অন্যান্য প্রাইভেট স্বাস্থ্য কেন্দ্র (Other private Health facility......................47<br>অন্যান্য (Others) ________________________________.98<br>(নির্দিষ্ট করুন)	
F05	*[মহিলার সর্বশেষ ডেলিভারীটি কোথায় হয়েছিল? বাড়ী/বাসায় নাকি স্বাস্থ্যকেন্দ্রে? প্রশ্ন নং F04 দেখে নিন।]*<br>*[Check F04, and indicate where did the last delivery took place?]*	বাড়ীতে/বাসায় At Home ....1<br>স্বাস্থ্যকেন্দ্রে Health Center 2	 →F08
F06	আপনার আত্মীয়দের মধ্য থেকে ডেলিভারীতে কেউ সাহায্য করেছিল কি?<br>Was there anyone of your family who assisted the delivery?	হ্যাঁ Yes...1<br>না No ....2<br>জানি না/ মনে নাই Don't know/Can't remember 9	 →F08<br>→F08
F07	আপনার আত্মীয়দের মধ্য থেকে কে ডেলিভারীতে সাহায্য করেছিল?<br><br>*[মহিলাকে জিজ্ঞেস করেন,]* আরও কেউ? *[মহিলার নিজে থেকে দেয়া সবগুলো উত্তরই বৃত্তায়িত করুন । উত্তরগুলো পড়ে শুনাবেন না। একাধিক উত্তর হতে পারে।]*<br><br>Who had assisted the delivery from among your family members? *[Don't prompt].*	মা Mother ....A<br>শ্বাশুড়ী Mother-in-law ....B<br>বোন/ননদ/জা Sister ....C<br>চাচী/মামী/খালা/ফুফুAunt ....D<br>দাদী/নানী Grandmother .... E<br>ভাগনি/ভাতিজী Niece .... F<br>স্বামী Husband ....G<br>অন্য কোন মহিলা আত্মীয় other female relative ____________________.. X<br>অন্য কোন পুরুষ আত্মীয় other male relative________________________Y	
F08	আপনার আত্মীয় নন এমন কেউ ডেলিভারীতে সাহায্য করেছিল কি?<br>Was there anyone from outside your family who assisted the delivery?	হ্যাঁ Yes...1<br>না No ....2<br>জানি না/ মনে নাই Don't know/Can't remember 9	 →F10<br>→F10

No	Questions and filters	Options and coding category	Skip
F09	আপনার আত্মীয় নন এমন কে আপনার ডেলিভারীতে সাহায্য করেছিল?<br><br>*[মহিলাকে জিজ্ঞেস করুন]* আরও কেউ? *[মহিলার নিজে থেকে দেয়া সবগুলো উত্তরই বৃত্তায়িত করুন । উত্তরগুলো পড়ে শুনাবেন না। একাধিক উত্তর হতে পারে।]*<br><br>*[যদি মহিলার উত্তর সি,এস,বি,এ (Code E) বা MNCS প্রমোটর (Code H) হয়, তাহলে তাদের নাম লিখুন।]*<br><br>**Code :E** নাম Name: ________________________________<br><br>**Code :H** নাম Name: ________________________________<br><br>Who from outside your family assisted in your delivery? *[Don't prompt. If the answer is either code E or H, write down their name.]*	**দক্ষ/প্রশিক্ষণ প্রাপ্ত স্বাস্থ্য কর্মী (Medically trained)**<br>পাশ করা ডাক্তার (MBBS doctor) .... A<br>নার্স/ধাত্রী (Nurse/midwife) .... B<br>প্যারামেডিক/মেডিকেল এসিসটেন্ট/সাকমো (Paramedic/MA/SACMO) .... C<br>পরিবার কল্যাণ পরিদর্শক (FWV) .... D<br>সি,এস,বি,এ (CSBA) .... E<br>**অন্যান্য স্বাস্থ্য কর্মী (Other health worker)**<br>স্বাস্থ্য সহকারী/ পরিবার কল্যান সহকারী (HA /FWA) .... F<br>পুষ্টি কর্মী (CNP) .... G<br>MNCS প্রমোটর (MNCS Promoter) .... H<br>অন্যান্য কমিউনিটি স্বাস্থ্য কর্মী - এনজিও কর্মী, স্বেচ্ছাসেবী (Other CHWs, NGO worker, volunteer) .... I<br>**অন্যান্য (Other)**<br>প্রশিক্ষণ প্রাপ্ত টিবিএ (প্রশিক্ষণ প্রাপ্ত ধন্নী, চাউনী, দাই) (TTBA) .... J<br>প্রশিক্ষণহীন টিবিএ (ধন্নী, চাউনী, দাই) TBA(Dai/Dhorni/Chauni) .... K<br>হোমিওপ্যাথ/হোমিওপ্যাথ ঔষধের দোকান (Homeopath/Homeopath drug store) .... L<br>আয়ুর্বেদিক চিকিৎসক / আয়ুর্বেদিক ঔষধের দোকান /হেকিম/কবিরাজ (Ayurved/ Ayurvedic drug store /Hekim/Kabiraj) .... M<br>গ্রাম ডাক্তার (Village doctor) .... N<br>এলোপ্যাথী ঔষধের দোকান (Allopath drug store) .... O<br>ইমাম/ঝাড় ফুক/ওঝা (Spiritual healer) .... P<br>পরিবারের অন্যান্য সদস্য/আত্মীয়/ প্রতিবেশী/বন্ধু Family/relative/Neighbor/friend .... Q<br>অন্যান্য Others ______________________ .. X<br>(নির্দিষ্ট করুন)<br>জানি না/মনে নাই Don't know/can't remember .... Z	
F10	আপনার সর্বশেষ এই ডেলিভারীর সময়ে কি কোন সমস্যা/জটিলতা হয়েছিল?<br><br>*[মহিলাকে প্রতিটি সমস্যা/জটিলতা পড়ে শোনান এবং উত্তরটি বৃত্তায়িত করুন।]*<br><br>Around the time of birth of <Name>, did you have any of the following problems : [Ask all the questions of the list]	Y N DK<br>দীর্ঘ / প্রলম্বিত প্রসব (১২ ঘন্টার বেশী) ব্যথা ছিল prolong labor 12 hr 1....2....9<br>বাধাগ্রস্থ প্রসব Obstructed labour .... 1....2....9<br>অতিরিক্ত রক্তস্রাব Excessive Vaginal Bleeding .... 1....2....9<br>দুর্গন্ধযুক্ত স্রাব A bad smelling vaginal discharge .... 1....2....9<br>খিচুঁনী হয়েছিল/এক্লামসিয়া Convulsion/eclampsia .... 1....2....9<br>শিশুর হাত /পা আগে বের হয়ে এসেছিল Hand or Feet came first ..... 1....2....9<br>ফুল না পরা Retained Placenta .... 1....2....9<br>তীব্র মাথা ব্যথা Severe Headache .... 1....2....9<br>পা/মুখ ফুলে গিয়েছিল Oedema/swelling of feet or face .... 1....2....9<br>অন্যান্য Others______________________ .. 1....2....9<br>(নির্দিষ্ট করুন)	
F11	*F10 দেখুন এবং সঠিক কোড বৃত্তায়িত করুন।*<br>Check F10, Is/Are there any complication/s mentioned by the women?	এক বা একাধিক কোড 1 বৃত্তায়িত One or more codes is/are circled .... 1<br>একটিও কোড 1 বৃত্তায়িত হয়নি 1 has been circled nowhere 2	→ G01
F12	আপনার মতে সমস্যা/জটিলতাটি কি খুব মারাত্মক নাকি মোটমুটি নাকি সামান্য ছিল?<br>From your opinion, was this problem/complication severe or mild to moderate?	সামান্য ছিল Mild .... 1<br>মোটামুটি ছিল Moderate .... 2<br>মারাত্মক ছিল Severe .... 3<br>অন্যান্য Other : ______________________ ... 7<br>জানেন না Doesn't know 9	
F13	এই সমস্যা/জটিলতার জন্য আপনি কি কাউকে দেখিয়েছিলেন বা কারও সাহায্য নিয়েছিলেন?<br>Did you seek any sort of treatment for this problem/complication?	হ্যাঁ Yes .... 1<br>না No .... 2<br>জানি না/ মনে নাই Don't know/Can't remember 9	 → F19<br>→ G01

No	Questions and filters	Options and coding category	Skip
F14	এই সমস্যা/জটিলতার জন্য আপনি কাকে দেখিয়েছিলেন বা কার সাহায্য নিয়েছিলেন?<br><br>*[মহিলাকে জিজ্ঞেস করুন]* আরও কেউ? *[মহিলার নিজে থেকে দেয়া সবগুলো উত্তরই বৃত্তায়িত করুন । উত্তরগুলো পড়ে শুনাবেন না। একাধিক উত্তর হতে পারে।]*<br><br>*[যদি মহিলার উত্তর সি,এস,বি,এ (Code E) বা MNCS প্রমোটর (Code H) হয়, তাহলে তাদের নাম লিখুন।]*<br><br>**Code :E** নাম Name: ________________<br><br>**Code :H** নাম Name: ________________<br><br>From whom did you seek treatment for this problem/complication? [*Do not read out the answers. Ask: Anything else? Circle all the answers. if the answer is either code E or F, write the name]*	**দক্ষ/প্রশিক্ষণ প্রাপ্ত স্বাস্থ্য কর্মী (Medically trained)**<br>পাশ করা ডাক্তার (MBBS doctor) .... A<br>নার্স/ধাত্রী (Nurse/midwife)........ B<br>প্যারামেডিক/মেডিকেল এসিসটেন্ট/সাকমো (Paramedic/MA/SACMO) .... C<br>পরিবার কল্যাণ পরিদর্শক (FWV)........ D<br>সি,এস,বি,এ (CSBA) .... E<br>**অন্যান্য স্বাস্থ্য কর্মী (Other health worker)**<br>স্বাস্থ্য সহকারী/ পরিবার কল্যান সহকারী (HA /FWA) .... F<br>পুষ্টি কর্মী (CNP) .... G<br>MNCS প্রমোটর (MNCS Promoter)........ H<br>অন্যান্য কমিউনিটি স্বাস্থ্য কর্মী - এনজিও কর্মী, স্বেচ্ছাসেবী (Other CHWs, NGO worker, volunteer)........ I<br>**অন্যান্য (Other)**<br>প্রশিক্ষণ প্রাপ্ত টিবিএ (প্রশিক্ষণ প্রাপ্ত ধন্নী, চাউনী, দাই) (TTBA) .... J<br>প্রশিক্ষণহীন টিবিএ (ধন্নী, চাউনী, দাই) TBA(Dai/Dhorni/Chauni)........ K<br>হোমিওপ্যাথ/হোমিওপ্যাথ ঔষধের দোকান (Homeopath/Homeopath drug store)........ L<br>আয়ুর্বেদিক চিকিৎসক / আয়ুর্বেদিক ঔষধের দোকান /হেকিম/কবিরাজ (Ayurved/ Ayurvedic drug store /Hekim/Kabiraj) .... M<br>গ্রাম ডাক্তার (Village doctor)........ N<br>এলোপ্যাথী ঔষধের দোকান (Allopath drug store) .... O<br>ইমাম/ঝাড় ফুক/ওঝা (Spiritual healer)........ P<br>পরিবারের অন্যান্য সদস্য/আত্মীয়/ প্রতিবেশী/বন্ধু Family/relative/Neighbor/friend........ Q<br>অন্যান্য Others ________________ .. X<br>(নির্দিষ্ট করুন)<br>জানি না/মনে নাই Don't know/can't remember........ Z	
F15	এই সমস্যা/জটিলতার জন্য আপনি কোথায় দেখিয়েছিলেন বা সেবা কোথায় পেয়েছিলেন?<br><br>*[মহিলাকে জিজ্ঞেস করুন]* আরও কোথাও? *[মহিলার নিজে থেকে দেয়া সবগুলো উত্তরই বৃত্তায়িত করুন । উত্তরগুলো পড়ে শুনাবেন না। একাধিক উত্তর হতে পারে।]*<br><br>*[মহিলা যেখান থেকে সেবা পেয়েছেন, সেই স্বাস্থ্যকেন্দ্রের নাম লিখুন। যদি একাধিক জায়গা থেকে সেবা নিয়ে থাকেন, তাহলে সবগুলো জায়গারই নাম এবং কোড লিখুন।]*<br><br>Code :|____| নাম Name: ________________<br><br>Code :|____| নাম Name: ________________<br><br>Code :|____| নাম Name: ________________<br><br>Where did you go to seek care for this problem/complication? Do not read out the answers. Ask: Anything else? Circle all the answers	**বাড়ী (Home)**<br>নিজ বাড়ী, স্বামী/শ্বশুড় বাড়ী (Own home, husband/father in laws house)........ A<br>বাবার বাড়ী (My natal home) .... B<br>অন্য কোন বাড়ী (Others)________________ C<br>(নির্দিষ্ট করুন)<br>**সরকারী স্বাস্থ্য কেন্দ্র (Govt Health center)**<br>মেডিকেল কলেজ হাসপাতাল (Medical College Hospital) .... D<br>জেলা/সদর হাসপাতাল (District /Sadar Hospital) .... E<br>মা ও শিশু স্বাস্থ্য কেন্দ্র (MCWC)........ F<br>উপজেলা স্বাস্থ্য কমপ্লেক্স (UHC) .... G<br>ইউনিয়ন স্বাস্থ্য ও পরিবার কল্যাণ কেন্দ্র / সাব সেন্টার/আরডি (FWC/SC/RD) H<br>কমিউনিটি ক্লিনিক (Community clinic)........ I<br>সেটেলাইট ক্লিনিক/ ইপিআই কেন্দ্র (Satellite clinic/EPI centre)........ J<br>অন্যান্য সরকারী স্বাস্থ্য কেন্দ্র (Other Govt Health facility) .... K<br>**বেসরকারী স্বাস্থ্য কেন্দ্র (Non Govt Health center)**<br>এনজিও হাসপাতাল (NGO hospital)........ L<br>এনজিও স্থায়ী স্বাস্থ্য কেন্দ্র (NGO static health centre) .... M<br>এনজিও সেটেলাইট ক্লিনিক (NGO satellite clinic)........ N<br>পুষ্টি কেন্দ্র (NNP centre)........ O<br>অন্যান্য বেসরকারী স্বাস্থ্য কেন্দ্র (Other NGO Health facility)........ P<br>**প্রাইভেট (Private Health sector)**<br>হাসপাতাল/ ক্লিনিক (Hospital/clinic)........ Q<br>স্বাস্থ্য কেন্দ্র /ডিসপেনসারী (Health centre/Dispensary) .... R<br>এমবিবিএস ডাক্তারের চেম্বার (MBBS doctor's chamber)........ S<br>গ্রাম ডাক্তারের চেম্বার (Village doctor's chamber) .... T<br>প্যারামেডিক/মেডিকেল এসিসটেন্ট/সাকমোর চেম্বার (Paramedic/MA/SACMO chamber) U<br>এলোপ্যাথী ঔষধের দোকান (Allopath drug store) .... V<br>অন্যান্য প্রাইভেট_স্বাস্থ্য কেন্দ্র (Other private Health facility........ W<br>অন্যান্য (Others) ________________ .. X<br>(নির্দিষ্ট করুন)	

No	Questions and filters	Options and coding category	Skip
F16	এই সমস্যা/জটিলতার জন্য কাউকে দেখানোর ব্যাপারে কেউ কি আপনাকে পরামর্শ দিয়েছিল ?<br>Did anyone advise you or refer you to seek care for your complication?	হ্যাঁ Yes..1<br>না No..2<br>জানি না/ মনে নাই Don't know/Can't remember..9	 →G01<br>→G01
F17	এই সমস্যা/জটিলতার জন্য কাউকে দেখানোর ব্যাপারে কে আপনাকে পরামর্শ দিয়েছিল?<br><br>*[মহিলাকে জিজ্ঞেস করুন] আরও কেউ? [মহিলার নিজে থেকে দেয়া সবগুলো উত্তরই বৃত্তায়িত করুন । উত্তরগুলো পড়ে শুনাবেন না। একাধিক উত্তর হতে পারে।]*<br><br>*[যদি মহিলার উত্তর সি,এস,বি,এ (Code E) বা MNCS প্রমোটর (Code H) হয়, তাহলে তাদের নাম লিখুন।]*<br><br>**Code :E** নাম Name: ____________________<br><br>**Code :H** নাম Name: ____________________<br><br>Who had advised you or refered you to seek care for your omplication? *[Do not read out the answers. Ask: Anything else? Circle all the answers. If the answer is either E or F, write down the name.]*	**<u>দক্ষ/প্রশিক্ষণ প্রাপ্ত স্বাস্থ্য কর্মী (Medically trained)</u>**<br>পাশ করা ডাক্তার (MBBS doctor)..A<br>নার্স/ধাত্রী (Nurse/midwife)..B<br>প্যারামেডিক/মেডিকেল এসিসটেন্ট/সাকমো (Paramedic/MA/SACMO)........C<br>পরিবার কল্যাণ পরিদর্শক (FWV)..D<br>সি,এস,বি,এ (CSBA)..E<br>**<u>অন্যান্য স্বাস্থ্য কর্মী (Other health worker)</u>**<br>স্বাস্থ্য সহকারী/ পরিবার কল্যান সহকারী (HA /FWA)........................F<br>পুষ্টি কর্মী (CNP)..G<br>MNCS প্রমোটর (MNCS Promoter)..H<br>অন্যান্য কমিউনিটি স্বাস্থ্য কর্মী - এনজিও কর্মী, স্বেচ্ছাসেবী (Other CHWs, NGO worker, volunteer)..I<br>**<u>অন্যান্য (Other)</u>**<br>প্রশিক্ষণ প্রাপ্ত টিবিএ (প্রশিক্ষণ প্রাপ্ত ধন্নী, চাউনী, দাই) (TTBA)................J<br>প্রশিক্ষণহীন টিবিএ (ধন্নী, চাউনী, দাই) TBA(Dai/Dhorni/Chauni)..............K<br>হোমিওপ্যাথ/হোমিওপ্যাথ ঔষধের দোকান (Homeopath/Homeopath drug store)..L<br>আয়ুর্বেদিক চিকিৎসক / আয়ুর্বেদিক ঔষধের দোকান /হেকিম/কবিরাজ (Ayurved/ Ayurvedic drug store /Hekim/Kabiraj)..................M<br>গ্রাম ডাক্তার (Village doctor)..N<br>এলোপ্যাথী ঔষধের দোকান (Allopath drug store)..................O<br>ইমাম/ঝাড় ফুক/ওঝা (Spiritual healer)..P<br>পরিবারের অন্যান্য সদস্য/আত্মীয়/ প্রতিবেশী/বন্ধু Family/relative/Neighbor/friend.......Q<br>অন্যান্য Others ____________________..X<br>(নির্দিষ্ট করুন)<br>জানি না/মনে নাই Don't know/can't remember..Z	
F18	এই সমস্যা/জটিলতার জন্য কাউকে দেখানোর ব্যাপারে যিনি আপনাকে পরামর্শ করেছিলেন, তিনি কি আপনাকে কোন কাগজ (রেফারেল সিলিপ) দিয়েছিলেন? (রেফারেল সিলিপের একটি নমুনা দেখান)<br>Did the person who had referred you issue you a referral slip to show it to the health facility/carer ? (show the referral slip to the respondent)	হ্যাঁ Yes..1<br>না No..2<br>জানি না/ মনে নাই Don't know/Can't remember..9	→ G01<br>→ G01<br>→ G01
F19	আপনি কেন এই সমস্যা/জটিলতার জন্য কাউকে দেখান নি?<br><br>*[মহিলাকে জিজ্ঞেস করুন] আরও কোন কারন? [মহিলার নিজে থেকে দেয়া সবগুলো উত্তরই বৃত্তায়িত করুন । উত্তরগুলো পড়ে শুনাবেন না। একাধিক উত্তর হতে পারে।]*<br><br>Why did you not seek care for this complication? *[Do not read out the answers. Ask: Anything else? Circle all the answers].*	সেবার প্রয়োজন আছে বলে মনে হয় নি/সেবার প্রয়োজন নেই<br>Didn't think it was necessary to seek care..A<br>জানতাম না কোথায় যেতে হবে Not known where to go........................B<br>অনেক খরচ/ টাকা পয়সা ছিল না Too costly/ Lack of money........................C<br>স্বাস্থ্য কেন্দ্র বাসা হতে অনেক দুরে Too far from house........................D<br>যানবাহনের সমস্যা Transport problem..E<br>সাথে যাবার মত কেউ ছিল না No one accompanied........................F<br>স্বাস্থ্যকেন্দ্রে যাবার মত সময় ছিল না Not enough time to go........................G<br>পরিবার আমাকে জেতে দেন নি Family didn't allow me to go........................H<br>ধর্মে মানা/বাধা Religeous bar..I<br>স্বাস্থ্যকেন্দ্রের সেবাদানের সময় সীমা সুবিধাজনক নয় Service hr inconvenient ...J<br>স্বাস্থ্যকেন্দ্র বন্ধ ছিল/কোন স্বাস্থ্যকর্মী ছিলেন না HF found closed/nobody there.....K<br>স্বাস্থ্যকেন্দ্রে সেবা অনুন্নত মানের Poor quality of services at facility................L<br>স্বাস্থ্যকেন্দ্রে নিম্নমানের এবং অদক্ষ সেবাপ্রদানকারী Poor quality & staffs at HF ..M<br>স্বাস্থ্যকেন্দ্রে পর্দার অভাব Lack of privacy..N<br>স্বাস্থ্যকেন্দ্রের সেবাদানকারীদের ব্যবহার খারাপ Unpleasant behavior at center ...O<br>স্বাস্থ্যকেন্দ্রের অনেকক্ষন বসে থাকতে হয় সেবা পাবার জন্য Long queue at HF .P<br>স্বাস্থ্যকেন্দ্রের ঔষধ পত্র পাওয়া যায়না Inadequate drugs at the health center ....Q<br>অন্যান্য Others ____________________..X	

Section G: Immediate Newborn Care

This section contains some information about immediate newborn care and feeding practice

No	Questions and filters	Options and coding category	Skip
G01	*[প্রশ্ন নং D15এবং D16 দেখুন]*<br>*[মহিলার ১লা জুন, 2011 বা এর পরে হওয়া সর্বশেষ গর্ভের ফলাফল কি ছিল?]*<br>*[Check Q D15 and D16 for the answer. What was the outcome of your most recent pregnancy since June 2011].*	জীবিত জন্ম Live Birth ....1<br>মৃত জন্ম Still Birth....2<br>গর্ভপাত/গর্ভনষ্ট Abortion 3	 →H01<br>→I00a
G02	*[প্রশ্ন নং D13 দেখুন এবং ১লা জুন, 2011 -এর পর হওয়া সর্বশেষ জীবিত শিশুটির নাম এবং শিশুটির লাইন নং টি এখানে লিখুন।]*<br>*[CHECK D13, write down the name of line no of the most recent Alive child of the women born on 1st June 2011 or later.]*	নাম Name: ________________ |____|____| line no	
G03	(নাম) এর জন্মের কত মিনিট পর ফুল পড়েছিল?<br>How many minutes after delivery of <Name> the placenta was delivered?	মিনিট MINUTES .... |___|___| ফুল পড়েনি আমাকে হাসপাতালে নিয়ে যাওয়া হয়েছিল Placenta was not delivered, I was moved to the hospital ....95<br>জানি না/মনে নাই Don't know/can't remember 99	
G04	(নাম)-কে কখন মোছানো/শুকানো হয়েছিল, ফুল পড়ার আগে না-কি ফুল পড়ার পরে?<br>When the baby's body was wiped/dried, before delivery of placenta or after delivery of placenta?	ফুল পড়ার আগে Before delivery of placenta....1<br>ফুল পড়ার পর After delivery of placenta....2<br>কখনই মোছানো/শুকানো হয়নি Wasn't wiped ....3<br>জানি না/ মনে নাই Don't know/ can't remember 9	→G06<br>→G06<br><br>→G10
G05	যদি জন্মের পর (নাম)-কে কখনই শুকানো বা মোছানো না হয়ে থাকে, তাহলে কেন হয়নি?<br>If the child wasn't wiped after its birth, then why he/she wasn't wiped?	________________ |___|___| ________________ |___|___|	G10
G06	জন্মের কতক্ষন পর (নাম)-কে মোছানো/শুকানো করা হয়েছিল?<br>How many minutes /hours after delivery of <Name> he/she was wiped/dried?	মিনিট Minutes ....1 |___|___| ঘন্টা Hours....2 |___|___| জানি না Don't know/can't remember....99	

No	Questions and filters	Options and coding category	Skip
G07	জন্মের পর (নাম)-এর গা কে মুছিয়েছিল/শুকিয়েছিল?<br>*[শুধুমাত্র একটি উত্তর বৃত্তায়িত করুন]*<br>*[যদি মহিলার উত্তর সি,এস,বি,এ (Code 15) বা MNCS প্রমোটর (Code 23) হয়, তাহলে তাদের নাম লিখুন ।]*<br>**Code :15** নাম Name: ____________________________<br>**Code :23** নাম Name: ______________________________<br>Who wiped (dried) the baby? *[Circle only one code. If the answer is either code 15 or 23, write their name.]*	**দক্ষ/প্রশিক্ষণ প্রাপ্ত স্বাস্থ্য কর্মী (Medically trained)**<br>পাশ করা ডাক্তার (MBBS doctor) .... 11<br>নার্স/ধাত্রী (Nurse/midwife)....... 12<br>প্যারামেডিক/মেডিকেল এসিসটেন্ট/সাকমো (Paramedic/MA/SACMO) .... 13<br>পরিবার কল্যাণ পরিদর্শক (FWV)....... 14<br>সি,এস,বি,এ (CSBA) .... 15<br>**অন্যান্য স্বাস্থ্য কর্মী (Other health worker)**<br>স্বাস্থ্য সহকারী/ পরিবার কল্যান সহকারী (HA /FWA) .... 21<br>পুষ্টি কর্মী (CNP) .... 22<br>MNCS প্রমোটর (MNCS Promoter)....... 23<br>অন্যান্য কমিউনিটি স্বাস্থ্য কর্মী - এনজিও কর্মী, স্বেচ্ছাসেবী (Other CHWs, NGO worker, volunteer)....... 24<br>**অন্যান্য (Other)**<br>প্রশিক্ষণ প্রাপ্ত টিবিএ (প্রশিক্ষণ প্রাপ্ত ধন্নী, চাউনী, দাই) (TTBA) .... 31<br>প্রশিক্ষণহীন টিবিএ (ধন্নী, চাউনী, দাই) TBA(Dai/Dhorni/Chauni)....... 32<br>হোমিওপ্যাথ/হোমিওপ্যাথ ঔষধের দোকান (Homeopath/Homeopath drug store) .... 33<br>আয়ুর্বেদিক চিকিৎসক / আয়ুর্বেদিক ঔষধের দোকান /হেকিম/কবিরাজ (Ayurved/ Ayurvedic drug store /Hekim/Kabiraj)....... 34<br>গ্রাম ডাক্তার (Village doctor)....... 35<br>এলোপ্যাথী ঔষধের দোকান (Allopath drug store) .... 36<br>ইমাম/ঝাড় ফুক/ওঝা (Spiritual healer)....... 37<br>পরিবারের অন্যান্য সদস্য/আত্মীয়/প্রতিবেশী/বন্ধু Family/relative/Neighbor/friend....... 38<br>অন্যান্য Others ______________________ ..39<br>(নির্দিষ্ট করুন)<br>জানি না/মনে নাই Don't know/can't remember 99	
G08	জন্মের পর (নাম)-কে যে মুছিয়েছিলেন/শুকিয়েছিলেন তাকে কি এই কাজের জন্য আগে থেকেই ঠিক করে রাখা হয়েছিল?<br>Was this person selected for taking care of (wiping/drying) the newborn before delivery?	হ্যাঁ Yes....... 1<br>না No .... 2<br>জানি না/ মনে নাই Don't know/Can't remember 9	 →G10<br>→G10
G09	*[প্রশ্ন নং E31 চেক করুন এবং মহিলাকে জিজ্ঞেস করুন:]*<br>তিনিই কি সেই একই ব্যক্তি/মহিলা যার কথা আপনি আগে বলেছিলেন?<br>Check the anwer of E31 and look for consistency. Ask the mother: Is he/she the same person you have mentioned earler?	হ্যাঁ Yes....... 1<br>না No 2	
G10	জন্মের পর কখন <নাম> কে কাপড় দিয়ে মুড়িয়ে নেয়া হয়েছিল? ফুল পড়ার আগে না-কি ফুল পড়ার পরে?<br>After delivery, when was the baby wrapped with a cloth, before delivery of placenta or after delivery of placenta?	ফুল পড়ার আগে Before delivery of placenta....... 1<br>ফুল পড়ার পর After delivery of placenta....... 2<br>মুড়িয়ে নেয়া হয়নি Wasn't wrapped .... 3<br>জানি না/ মনে নাই Don't know/ can't remember 9	→G12<br>→G12<br><br>→G17
G11	যদি জন্মের পর পর <নাম> কে কখনই কাপড় দিয়ে মোড়ানো না হয়ে থাকে, তাহলে কেন মোড়াননি?<br>If the child wasn't wrapped after its birth, then why he/she wasn't wrapped?	______________________ |__|__| ______________________ |__|__|	G17
G12	জন্মের কতক্ষন পর (নাম) কে কাপড় দিয়ে মুড়িয়ে নেয়া হয়েছিল? [এক ঘন্টার নীচে হলে মিনিটে লিখুন]<br>How many minutes after delivery of <Name> was wrapped?	মিনিট Minutes .... 1 |__|__| ঘন্টা Hours....... 2 |__|__| জানি না Don't know/can't remember....... 99	

No	Questions and filters	Options and coding category	Skip
G13	জন্মের পর (নাম)-কে কাপড় দিয়ে কে মুড়িয়েছিল?<br>*[শুধুমাত্র একটি উত্তর বৃত্তায়িত করুন]*<br>*[যদি মহিলার উত্তর সি,এস,বি,এ (Code 15) বা MNCS প্রমোটর (Code 23) হয়, তাহলে তাদের নাম লিখুন।]*<br>**Code :15** নাম Name: ____________<br>**Code :23** নাম Name: ____________<br>Who wrapped the baby with a cloth? *[Circle only one code. If answer is either 15 or 23, write their name.]*	**দক্ষ/প্রশিক্ষণ প্রাপ্ত স্বাস্থ্য কর্মী (Medically trained)**<br>পাশ করা ডাক্তার (MBBS doctor) .... 11<br>নার্স/ধাত্রী (Nurse/midwife)........ 12<br>প্যারামেডিক/মেডিকেল এসিসটেন্ট/সাকমো (Paramedic/MA/SACMO) .... 13<br>পরিবার কল্যাণ পরিদর্শক (FWV)........ 14<br>সি,এস,বি,এ (CSBA) .... 15<br>**অন্যান্য স্বাস্থ্য কর্মী (Other health worker)**<br>স্বাস্থ্য সহকারী/ পরিবার কল্যান সহকারী (HA /FWA) .... 21<br>পুষ্টি কর্মী (CNP) .... 22<br>MNCS প্রমোটর (MNCS Promoter)........ 23<br>অন্যান্য কমিউনিটি স্বাস্থ্য কর্মী - এনজিও কর্মী, স্বেচ্ছাসেবী (Other CHWs, NGO worker, volunteer)........ 24<br>**অন্যান্য (Other)**<br>প্রশিক্ষণ প্রাপ্ত টিবিএ (প্রশিক্ষণ প্রাপ্ত ধন্নী, চাউনী, দাই) (TTBA) .... 31<br>প্রশিক্ষণহীন টিবিএ (ধন্নী, চাউনী, দাই) TBA(Dai/Dhorni/Chauni)........ 32<br>হোমিওপ্যাথ/হোমিওপ্যাথ ঔষধের দোকান (Homeopath/Homeopath drug store) .... 33<br>আয়ুর্বেদিক চিকিৎসক / আয়ুর্বেদিক ঔষধের দোকান /হেকিম/কবিরাজ (Ayurved/ Ayurvedic drug store /Hekim/Kabiraj)........ 34<br>গ্রাম ডাক্তার (Village doctor)........ 35<br>এলোপ্যাথী ঔষধের দোকান (Allopath drug store) .... 36<br>ইমাম/ঝাড় ফুক/ওঝা (Spiritual healer)........ 37<br>পরিবারের অন্যান্য সদস্য/আত্মীয়/প্রতিবেশী/বন্ধু Family/relative/Neighbor/friend........ 38<br>অন্যান্য Others ____________ ..39<br>(নির্দিষ্ট করুন)<br>জানি না/মনে নাই Don't know/can't remember........ 99	
G14	জন্মের পর (নাম)-কে যে মুড়িয়েছিলেন তাকে কি এই কাজের জন্য আগে থেকেই ঠিক করে রাখা হয়েছিল?<br>Was this person selected for taking care of (wrapping) the newborn before the delivery?	হ্যাঁ Yes........ 1<br>না No .... 2<br>জানি না/ মনে নাই Don't know/Can't remember 9	 →G16<br>→G16
G15	*[প্রশ্ন নং E31 চেক করুন এবং মহিলাকে জিজ্ঞেস করুন]*<br>তিনিই কি সেই একই ব্যক্তি/মহিলা যার কথা আপনি আগে বলেছিলেন?<br>Interviewer: Check the anwer of E29 and look for consistency. Ask the mother: Is he/she the same person you have mentioned earler?	হ্যাঁ Yes........ 1<br>না No 2	
G16	কি ধরনের কাপড় দিয়ে জন্মের পর (নাম)-কে মুড়ানো হয়েছিলো?<br>What kinds of cloths were used to wrap the child?	একটি নতুন কেনা কাপড় A new cloth........ 1<br>ঘরের পরিস্কার কাপড় An fresh and clean household cloth........ 2<br>ঘরের যে কোন কাপড় Any household colth........ 3<br>অন্যান্য Others ____________ . 7<br>জানা নাই/মনে নাই Don't know/can't remember........ 9	
G17	শিশুর জন্মের পর নাড়ী কি দিয়ে কাটা উচিত বলে আপনি মনে করেন?<br>*[কি দিয়ে নাড়ী কাটা উচিত, এই প্রশ্নের উত্তর একটি হবে]*<br>What should be used to cut the cord after the birth?*[Select only one answer]*	ডেলিভারী ব্যাগ/কিট এর ব্লেড Blade from the delivery kit .... 1<br>নতুন ব্লেড New blade........ 2<br>গরম পানিতে ফুটানো ব্লেড boiled blade .... 3<br>বাড়ীর পুরাতন ব্লেড Old blade in the house .... 4<br>বাঁশের কঞ্চি/বাত/টল Bamboo strips/ bata/toll........ 5<br>কাঁচি Scissor .... 6<br>অন্যান্য Others ____________ ....8<br>জানা নাই/মনে নাই Don't know/can't remember........ 9	

No	Questions and filters	Options and coding category	Skip
G18	শিশুর জন্মের পর নাড়ী কাটা এবং বাঁধার পর তাতে কি কিছু দেয়া উচিত? কি দেয়া উচিত বলে আপনি মনে করেন?<br><br>*[নাড়ী কাটা ও বাধার পর কি লাগানো উচিত, এই উত্তরের বেলায় মহিলাকে জিজ্ঞেস করুন]* আরও কিছু? *[মহিলার নিজে থেকে দেয়া সবগুলো উত্তরই বৃত্তায়িত করুন । উত্তরগুলো পড়ে শুনাবেন না। একাধিক উত্তর হতে পারে।]*<br><br>What should be applied after cutting the tying the cord. Don't prompt.	কোন কিছুই দেয়া উচিত নয় Nothing should be applied ....A<br>অ্যান্টিবায়োটিক (পাউডার/মলম) Antibiotics (Powder / Ointment) ....B<br>অ্যান্টিসেপটিক (ডেটল/স্যাভলন/হেক্সাসল) Antiseptic (Detol/savlon/hexisol) .... C<br>স্পিরিট/এলকোহল Spirit/Alcohol ....D<br>সরিষার তেল (রসুন সহ বা বাদে) Mustard oil(with or without garlic)............ E<br>চিবানো চাল Chewed rice .... F<br>হলুদের রস/গুড়া Turmeric juice/powder...G<br>আদার রস Ginger juice ....H<br>সিঁদুর Shidur .... I<br>বরিক পাউডার Boric powder .... J<br>জেনসিয়ান ভায়োলেট/নীল কালি Gentian violet/Blue ink............................K<br>ট্যালকম পাউডার Talcom Powder.. L<br>ছাই Ash ....M<br>নারিকেল তেল Coconut oil ....N<br>চুলার পোড়া মাটি Dust of earth-burner ....O<br>অন্যান্য Other _______________________________________ X<br>জানা নাই/মনে নাই Don't know/can't remember .... Y	
G19	*[প্রশ্ন নং G17 এবং G18দেখুন। মহিলা যদি এক বা একাধিক উত্তর দিয়ে থাকেন, তাহলে প্রশ্ন করুন:]*<br><br>জন্মের পর একটি শিশুর নাড়ী কাটা ও যত্ন কিভাবে নিতে হয়, তা আপনি বললেন -- এখন আমাকে বলুন যে, এই সব তথ্য আপনি কোথা থেকে বা কার কাছ থেকে জেনেছেন?<br><br>*[মহিলাকে জিজ্ঞেস করুন]* আরও কিছু? *[মহিলার নিজে থেকে দেয়া সবগুলো উত্তরই বৃত্তায়িত করুন । উত্তরগুলো পড়ে শুনাবেন না। একাধিক উত্তর হতে পারে।]*	ডাক্তার/নার্স/ধাত্রী/প্যারামেডিক (Doctor/ Nurse/Midwife/ Paramedics) ....A<br>স্বাস্থ্য কেন্দ্র/হাসপাতাল (Health facility/ Clinic/ Hospital)............................B<br>কমিউনিটি স্বাস্থ্য কর্মী - স্বাস্থ্য সহকারী/পরিবার কল্যান সহকারী, পুষ্টি কর্মী, এনজিও কর্মী, MNCS প্রমোটর, স্বেচ্ছাসেবী (CHWs -HAS, FWAs, CNPs, MNCS promoter,NGO worker, volunteer)..C<br>কমিউনিটি গ্রুপ মিটিং / মিটিং /সভা থেকে (Community meeting) ....D<br>রেডিও/টিভি (Radio /TV) ....E<br>সংবাদপত্র/খবরের কাগজ/বইপত্র (Newspaper/Books) ....F<br>পোষ্টার / কোন ছাপানো কাগজ (Poster/Leaflet)...G<br>পরিবারের অন্যান্য সদস্য/আত্মীয়/প্রতিবেশী/বন্ধু Family/relatives/Neighbor/friend .....H<br>অন্যান্য Others_______________________________________...X<br>(নির্দিষ্ট করুন)<br>মনে নাই Can't remember ....Y	
G20	জন্মের পর (নাম)-এর নাড়ী কি দিয়ে কাটা হয়েছিল?<br>What was used to cut the cord? Circle only one code	ডেলিভারী ব্যাগ/কিট এর ব্লেড Blade from the delivery kit ....1<br>নতুন ব্লেড New blade..2<br>গরম পানিতে ফুটানো ব্লেড boiled blade ....3<br>বাড়ীর পুরাতন ব্লেড Old blade in the house ....4<br>বাঁশের কঞ্চি/বাত/টল Bamboo strips/ bata/toll...5<br>কাঁচি Scissor ....6<br>অন্যান্য Others _______________________________________....8<br>জানা নাই/মনে নাই Don't know/can't remember 9	 <br><br><br><br><br>→G24<br>→G24
G21	জন্মের পর নাড়ী কাটার আগে নাড়ি কাটার জিনিসপত্র গুলো সিদ্ধ করে/পানিতে ফুটিয়ে নেয়া হয়েছিল কি?<br>Was the instrument boiled before cutting the cord?	হ্যাঁ Yes...1<br>না No ....2<br>জানি না/ মনে নাই Don't know/Can't remember 9	
G22	জন্মের পর নাড়ী কাটা এবং বাঁধার পর নাড়িতে কোন কিছু দেয়া হয়েছিল কি?<br>Was anything applied to the cord after cutting and tying it?	হ্যাঁ Yes...1<br>না No ....2<br>জানি না/ মনে নাই Don't know/Can't remember 9	 →G24<br>→G24

No	Questions and filters	Options and coding category	Skip
G23	জন্মের পর নাড়ী কাটা এবং বাঁধার পর নাড়ীতে কি দেয়া হয়েছিল?<br><br>*[মহিলাকে জিজ্ঞেস করুন]* আরও কিছু? *[মহিলার নিজে থেকে দেয়া সবগুলো উত্তরই বৃত্তায়িত করুন । উত্তরগুলো পড়ে শুনাবেন না । একাধিক উত্তর হতে পারে ।]*<br><br>What was applied to the cord after cutting and tying the cord? *[Do not read out the answers, Ask: Anything else?, Circle all the answers]*	কোন কিছুই দেয়া হয়নি Nothing was applied .... A<br>অ্যান্টিবায়োটিক (পাউডার/মলম) Antibiotics (Powder / Ointment) .... B<br>অ্যান্টিসেপটিক (ডেটল/স্যাভলন/হেক্সাসল) Antiseptic (Detol/savlon/hexisol) .... C<br>স্পিরিট/এলকোহল Spirit/Alcohol .... D<br>সরিষার তেল (রসুন সহ বা বাদে) Mustard oil(with or without garlic) .... E<br>চিবানো চাল Chewed rice .... F<br>হলুদের রস/গুড়া Turmeric juice/powder .... G<br>আদার রস Ginger juice .... H<br>সিঁদূর Shidur .... I<br>বরিক পাউডার Boric powder .... J<br>জেনসিয়ান ভায়োলেট/নীল কালি Gentian violet/Blue ink .... K<br>ট্যালকম পাউডার Talcom Powder .... L<br>ছাই Ash .... M<br>নারিকেল তেল Coconut oil .... N<br>চুলার পোড়া মাটি Dust of earth-burner .... O<br>অন্যান্য Other ____________ X<br>জানা নাই/মনে নাই Don't know/can't remember .... Y	
G24	জন্মের পর পরই (নাম) কি স্বাভাবিক ভাবে কেঁদেছিল/শ্বাস নিয়েছিল?<br>Did <name> cry/ breathe normally immediately after birth?	হ্যাঁ Yes .... 1<br>না No .... 2<br>জানি না/ মনে নাই Don't know/Can't remember 9	→G27
G25	জন্মের পর পরই (নাম)-কে কাঁদানোর জন্য বা শ্বাস নেয়ানোর জন্য কিছু করতে হয়েছিল কি?<br><br>*[মহিলাকে জিজ্ঞেস করুন]* আরও কিছু? *[মহিলার নিজে থেকে দেয়া সবগুলো উত্তরই বৃত্তায়িত করুন । উত্তরগুলো পড়ে শুনাবেন না । একাধিক উত্তর হতে পারে ।]*<br><br>Was anything done to help the baby cry or breath immediately after birth? Do not read out the answers, Ask: Anything else? Circle all the answers	বাচ্চার গা শুকানো হয়েছে Dried the baby .... A<br>বাচ্চাকে মুড়িয়ে নেয়া হয়েছে Wrapped the baby .... B<br>বাচ্চার পিঠে ঘষা হয়েছে Rubbed the back .... C<br>বাচ্চার পায়ের পাতা ঘষা হয়েছে Rubbed the feet .... D<br>মুখ থেকে মুখে শ্বাস নেয়ানোর চেষ্টা করা হয়েছে Mouth to mouth breathing ..... E<br>নাড়ীতে তাপ দেয়া হয়েছে Heated the cord .... F<br>বাচ্চাকে থাপ্পড় দেয়া হয়েছে Slapped the baby .... G<br>বাচ্চার মাথা নীচ দিকে দিয়ে ঝুলানো হয়েছে Hold the baby upside down .... H<br>অন্যান্য Other ____________ . X<br>নির্দিষ্ট করুন<br>কিছু করা হয় নাই Nothing done .... Y<br>জানা নাই/মনে নাই Don't know/Can't remember .... Z	 <br><br><br><br><br><br><br><br><br>→G27<br>→G27

No	Questions and filters	Options and coding category	Skip
G26	জন্মের পর (নাম)-কে কাঁদানোর/শ্বাস নেয়ানোর জন্য কে চেষ্টা বা কিছু করেছিল?<br>*[শুধুমাত্র একটি কোড বৃত্তায়িত করুন]*<br>*[যদি মহিলার উত্তর সি,এস,বি,এ (Code 15) বা MNCS প্রমোটর (Code 23) হয়, তাহলে তাদের নাম লিখুন।]*<br>**Code :15** নাম Name: ______________________________<br>**Code :23** নাম Name: ______________________________<br>Who took initiative to resuscitate or to help the baby cry? *[Circle only one cod. If answer is either 15 or 23, write their name.]*	**দক্ষ/প্রশিক্ষণ প্রাপ্ত স্বাস্থ্য কর্মী (Medically trained)**<br>পাশ করা ডাক্তার (MBBS doctor) .... 11<br>নার্স/ধাত্রী (Nurse/midwife)........ 12<br>প্যারামেডিক/মেডিকেল এসিসটেন্ট/সাকমো (Paramedic/MA/SACMO) .... 13<br>পরিবার কল্যাণ পরিদর্শক (FWV)........ 14<br>সি,এস,বি,এ (CSBA) .... 15<br>**অন্যান্য স্বাস্থ্য কর্মী (Other health worker)**<br>স্বাস্থ্য সহকারী/ পরিবার কল্যান সহকারী (HA /FWA) .... 21<br>পুষ্টি কর্মী (CNP) .... 22<br>MNCS প্রমোটর (MNCS Promoter)........ 23<br>অন্যান্য কমিউনিটি স্বাস্থ্য কর্মী - এনজিও কর্মী, স্বেচ্ছাসেবী (Other CHWs, NGO worker, volunteer)........ 24<br>**অন্যান্য (Other)**<br>প্রশিক্ষণ প্রাপ্ত টিবিএ (প্রশিক্ষণ প্রাপ্ত ধন্নী, চাউনী, দাই) (TTBA) .... 31<br>প্রশিক্ষণহীন টিবিএ (ধন্নী, চাউনী, দাই) TBA(Dai/Dhorni/Chauni)........ 32<br>হোমিওপ্যাথ/হোমিওপ্যাথ ঔষধের দোকান (Homeopath/Homeopath drug store) .... 33<br>আয়ুর্বেদিক চিকিৎসক / আয়ুর্বেদিক ঔষধের দোকান /হেকিম/কবিরাজ (Ayurved/ Ayurvedic drug store /Hekim/Kabiraj)........ 34<br>গ্রাম ডাক্তার (Village doctor)........ 35<br>এলোপ্যাথী ঔষধের দোকান (Allopath drug store) .... 36<br>ইমাম/ঝাড় ফুক/ওঝা (Spiritual healer)........ 37<br>পরিবারের অন্যান্য সদস্য/আত্মীয়/প্রতিবেশী/বন্ধু Family/relative/Neighbor/friend... 38<br>অন্যান্য Others ______________________ ..39<br>(নির্দিষ্ট করুন)<br>জানি না/মনে নাই Don't know/can't remember 99	
G27	জন্মের পর পর (নাম)-এর ওজন কি নেয়া হয়েছিল?<br>*[(নাম)-এর জন্ম হাসপাতাল বা কোন স্বাস্থ্যকেন্দ্রে হলেও মাকে এই প্রশ্নটি করুন।]*<br>Do you know whether the birth weight was taken just after the birth of NAME? (even if the child was born in the hospital)	হ্যাঁ Yes........ 1<br>না No .... 2<br>জানি না/ মনে নাই Don't know/Can't remember 9	 →G29<br>→G29
G28	জন্মের পর (নাম)-এর ওজন কত ছিল?<br>Can you tell me what the birth weight of NAME was?	জন্ম ওজন, কেজি-তে (measured in KG) .... 1 |___|.|___|___| KG<br>জন্ম ওজন, পাউন্ড-এ (measured in LB) .... 2 |___|.|___|___| LB<br>জানি না/মনে নাই Don't know/CR 9999	
G29	জন্মের পর (নাম) আকারে কতটুকু ছিল? স্বাভাবিকের চেয়ে অনেক ছোট, নাকি স্বাভাবিকের চেয়ে একটু ছোট, নাকি স্বাভাবিক, নাকি স্বাভাবিকের চেয়ে বড়?<br>Was the size of your baby according to you?	অনেক ছোট Much smaller than usual .... 1<br>স্বাভাবিকের থেকে ছোট Smaller than usual .... 2<br>স্বাভাবিক Normal/Usual .... 3<br>স্বাভাবিকের থেকে বড় Larger than usual .... 4<br>ছোট না বড়, বুঝি নাই Can't decide 8	
G30	জন্মের কত সময় পর (নাম)-কে প্রথম গোসল করানো হয়েছিল?<br>*[যদি এক ঘন্টার কম হয়, তাহলে ঘন্টার ঘরে 00 লিখুন, যদি এক দিনের কম হয়, তাহলে ঘন্টায় রেকর্ড করুন, যদি এক সপ্তাহের কম হয়, তাহলে দিনে রেকর্ড করুন]*<br>How long after delivery was (NAME) bathed for the first time?	ঘন্টা Hours .... 1 |___|___| দিন Days .... 2 |___|___| সপ্তাহ Weeks .... 3 |___|___| জানি না Don't know/can't remember 999	
G31	শিশুর জন্মের পর পর শিশুটির শরীর গরম রাখা প্রয়োজনীয় কি? কিভাবে শিশুর শরীর গরম রাখা যায়?<br>*[মহিলাকে জিজ্ঞেস করুন]* আরও কিছু? *[মহিলার নিজে থেকে দেয়া সবগুলো উত্তরই বৃত্তায়িত করুন । উত্তরগুলো পড়ে শুনাবেন না। একাধিক উত্তর হতে পারে।]*<br>Do you know, what should be done to keep the baby warm? *[Don't prompt]*	শুকানো Dried the baby .... A<br>কাপড়/কাঁথা/তোয়ালে দিয়ে মুড়ানো Wrapped the baby .... B<br>বুকের চামড়ার উপরে বাচ্চাকে চেপে রাখা<br>Kept the baby on bare skin to skin contact........ C<br>রান্নাঘরে ডেলিভারী করানো Conducted delivery at kitchen .... D<br>ডেলিভারী কক্ষে আগুন জ্বালিয়ে রাখার ব্যবস্থা করা<br>Lighted some fire at delivery room........ E<br>গরম তেল শরীরে মালিশ করা Rubbed the baby with hot oil .... F<br>আমার কোলে বাচ্চাকে রাখা Kept newborn on my lap .... G<br>অন্য কারো কোলে বাচ্চাকে রাখা Kept newborn at others lap .... H<br>জন্মের পর শিশুকে গরম রাখার প্রয়োজন নাই No need to keep baby warm .... I<br>অন্যান্য Others ______________________ X<br>নির্দিষ্ট করুন	→G33

No	Questions and filters	Options and coding category	Skip
G32	জন্মের পর শিশুকে গরম রাখার এই সব তথ্য আপনি কোথা থেকে বা কার কাছ থেকে জেনেছেন?<br><br>*[মহিলাকে জিজ্ঞেস করুন]* আরও কিছু? *[মহিলার নিজে থেকে দেয়া সবগুলো উত্তরই বৃত্তায়িত করুন । উত্তরগুলো পড়ে শুনাবেন না। একাধিক উত্তর হতে পারে।]*<br><br>Where did you get this knowledge/information? *[don't prompt]*	ডাক্তার/নার্স/ধাত্রী/প্যারামেডিক (Doctor/ Nurse/Midwife/ Paramedics) ....A<br>স্বাস্থ্য কেন্দ্র/হাসপাতাল (Health facility/ Clinic/ Hospital)............................B<br>কমিউনিটি স্বাস্থ্য কর্মী - স্বাস্থ্য সহকারী/পরিবার কল্যান সহকারী, পুষ্টি কর্মী, এনজিও কর্মী, MNCS প্রমোটর, স্বেচ্ছাসেবী (CHWs -HAS, FWAs, CNPs, MNCS promoter,NGO worker, volunteer)..C<br>কমিউনিটি গ্রুপ মিটিং / মিটিং /সভা থেকে (Community meeting) ....D<br>রেডিও/টিভি (Radio /TV) ....E<br>সংবাদপত্র/খবরের কাগজ/বইপত্র (Newspaper/Books) ....F<br>পোষ্টার / কোন ছাপানো কাগজ (Poster/Leaflet)..G<br>পরিবারের অন্যান্য সদস্য/আত্মীয়/প্রতিবেশী/বন্ধু Family/relatives/Neighbor/friend .....H<br>অন্যান্য Others________________________________...X<br>(নির্দিষ্ট করুন)<br>মনে নাই Can't remember ....Y	
G33	জন্মের পর পর (নাম)-এর শরীর গরম রাখার জন্য কি কিছু করেছিলেন?<br>After (NAME) was born, did you do anything to keep (NAME) warm?	হ্যাঁ Yes..1<br>না No ....2<br>জানি না/ মনে নাই Don't know/Can't remember 9	 →G35<br>→G35
G34	জন্মের পর পর (নাম)-এর শরীর গরম রাখার জন্য কি করেছিলেন?<br><br>*[মহিলাকে জিজ্ঞেস করুন]* আরও কিছু? *[মহিলার নিজে থেকে দেয়া সবগুলো উত্তরই বৃত্তায়িত করুন । উত্তরগুলো পড়ে শুনাবেন না। একাধিক উত্তর হতে পারে।]*<br><br>What have you done to keep (NAME) warm following delivery? *[Do not read out the answers, Ask: Anything else? Circle all the answers]*	শুকানো Dried the baby ....A<br>কাপড়/কাঁথা/তোয়ালে দিয়ে মুড়ানো Wrapped the baby ....B<br>বুকের চামড়ার উপরে বাচ্চাকে চেপে রাখা Kept the baby on bare skin to skin contact..C<br>রান্নাঘরে ডেলিভারী করানো Conducted delivery at kitchen ....D<br>ডেলিভারী কক্ষে আগুন জ্বালিয়ে রাখার ব্যবস্থা করা Lighted some fire at delivery room.. E<br>গরম তেল শরীরে মালিশ করা Rubbed the baby with hot oil .... F<br>আমার কোলে বাচ্চাকে রাখা Kept newborn on my lap ....G<br>অন্য কারো কোলে বাচ্চাকে রাখা Kept newborn at others lap ....H<br>অন্যান্য Others ________________________________X<br>নির্দিষ্ট করুন	
G35	জন্মের প্রথম 15 দিনের মধ্যে রাতে ঘুমানোর সময় (নাম)-কে কি বুকে রাখতেন, নাকি একই বিছানায় রাখতেন, নাকি আলাদা রাখতেন?<br>In the first fifteen days do you sleep with (NAME) against your chest at night, or do you lay him/her on the bed/cot, or elsewhere?	বাচ্চাকে বুকে রাখতাম Kept baby on chest ....1<br>বাচ্চাকে একই বিছানায় রাখতাম Lay baby on same cot ....2<br>বাচ্চাকে একই ঘরে অন্য বিছানায় রাখতাম lay baby on separate cot...............3<br>আলাদা অন্য ঘরে রাখতাম different room ....4<br>জানি না Don't know 9	 →G37<br>→G37<br>→G37<br>→G37
G36	জন্মের 15 দিনের মধ্যে দিন এবং রাতের বেলায় প্রতিদিন কতক্ষন (নাম)-এর খালি বুক আপনার বুকের মধ্যে (খালি বুকে) রেখেছিলেন?<br>In the first fifteen days of life how frequently per day did you hold (NAME) skin-to-skin against your breasts/bare chest during the daytime and nighttime?	ঘন্টা Hours .... 1 |___|___| মিনিট Minutes .... 2 |___|___| জানি না/মনে নাই Don't know/Can't remember.................................. 999	
G37	আপনি কি জানেন, জন্মের পর শিশুকে কখন প্রথম বুকের দুধ খাওয়ানো উচিত?<br>When breast-feeding should be started for a newborn?	জন্মের পর পর Immediately ....000<br>ঘন্টা Hours .... 1 |___|___| দিন Days .... 2 |___|___| জানি না কত সময় পর খাওয়ানো উচিত Don't know 999	 <br><br>→ G39
G38	আপনি বললেন যে, জন্মের ---- (সময়) পর শিশুকে বুকের দুধ দেয়া উচিত। এই তথ্য আপনি কোথা থেকে বা কার কাছ থেকে জেনেছেন?<br>Where did you get this knowledge?	ডাক্তার/নার্স/ধাত্রী/প্যারামেডিক (Doctor/ Nurse/Midwife/ Paramedics) ....A<br>স্বাস্থ্য কেন্দ্র/হাসপাতাল (Health facility/ Clinic/ Hospital)............................B<br>কমিউনিটি স্বাস্থ্য কর্মী - স্বাস্থ্য সহকারী/পরিবার কল্যান সহকারী, পুষ্টি কর্মী, এনজিও কর্মী, MNCS প্রমোটর, স্বেচ্ছাসেবী (CHWs -HAS, FWAs, CNPs, MNCS promoter,NGO worker, volunteer)..C<br>কমিউনিটি গ্রুপ মিটিং / মিটিং /সভা থেকে (Community meeting) ....D<br>রেডিও/টিভি (Radio /TV) ....E<br>সংবাদপত্র/খবরের কাগজ/বইপত্র (Newspaper/Books) ....F<br>পোষ্টার / কোন ছাপানো কাগজ (Poster/Leaflet)..G<br>পরিবারের অন্যান্য সদস্য/আত্মীয়/প্রতিবেশী/বন্ধু Family/relatives/Neighbor/friend .....H<br>অন্যান্য Others________________________________...X<br>(নির্দিষ্ট করুন)<br>মনে নাই Can't remember ....Y	

No	Questions and filters	Options and coding category	Skip
G39	(নাম)-কে কি কখনও বুকের দুধ খাইয়েছিলেন?<br>Have you ever breastfed NAME?	হ্যাঁ Yes....................1<br>না No....................2	 →H01
G40	ফুল পড়া বা বের হওয়ার আগেই কি (নাম)-কে বুকের দুধ খাওয়ানো শুরু করেছিলেন?<br>Was the baby put to breast before delivery of the placenta?	হ্যাঁ Yes....................1<br>না No....................2<br>জানি না/ মনে নাই Don't know/Can't remember....................9	
G41	জন্মের কতক্ষন পর (নাম)-কে প্রথম বুকের দুধ দিতে শুরু করেছিলেন?<br>*[জন্মের 1 ঘন্টার মধ্যে হলে 000 বৃত্তায়িত করুন। 24 ঘন্টার মধ্যে হলে 1 বৃত্তায়িত করে বক্সে সঠিক ঘন্টা লিখুন। 24 ঘন্টা বা তার অধিক হলে কোড 2 বৃত্তায়িত করে উত্তর দিনে লিখুন।]*<br>When was the baby breast-fed for the first time after birth? If before/within an hour circle "000", If before/ within 24 hours then circle 1 and write in the space for hour, If 24 hours/after 24 hours then circle 2 and write in the space for days	জন্মের পর পর Immediately....................000<br>ঘন্টা Hours.................... 1 |___|___| দিন Days.................... 2 |___|___|	
G42	(নাম)-কে কি জন্মের পর শাল দুধ খাওয়ানো হয়েছিল?<br>Was (NAME) given colostrums immediately after birth?	হ্যাঁ Yes....................1<br>না No....................2<br>জানি না/ মনে নাই Don't know/Can't remember....................9	
G43	(নাম)-এর জন্মের পর প্রথম ৩ দিন বুকের দুধ ছাড়া অন্য কিছু খাওয়ানো বা পান করতে দেয়া হয়েছিল কি?<br>Was the baby given any other food or liquid before starting breast milk?	হ্যাঁ Yes....................1<br>না No....................2<br>জানি না/ মনে নাই Don't know/Can't remember....................9	 →H01<br>→H01
G44	জন্মের পর প্রথম ৩ দিন বুকের দুধ ছাড়া (নাম)-কে কি কি খাবার বা পানীয় খাওয়ানো হয়েছিল?<br>*[মহিলাকে জিজ্ঞেস করুন]* আরও কিছু? *[মহিলার নিজে থেকে দেয়া সবগুলো উত্তরই বৃত্তায়িত করুন । উত্তরগুলো পড়ে শুনাবেন না। একাধিক উত্তর হতে পারে।]*<br>What other food or liquid was given before giving breast milk? Ask: Did you feed any other things? Write down all the answers	মধু Honey....................A<br>মিশ্রীর/চিনির পানি Misri/Sugar water....................B<br>শুধু পানি Water....................C<br>ফলের রস Fruit juice....................D<br>টিনজাত দুধ/শিশু খাদ্য (বেবী ফর্মূলা) Tinned milk/ infant formula from shop....E<br>গরু/ছাগলের দুধ Cow's milk....................F<br>চা Tea....................G<br>গ্রাইপ ওয়াটার Gripe water....................H<br>অন্যান্য তরল Other liquids....................I<br>লেই (পানিতে মিশানো চালের গুড়া, আটা, ময়দা) Rice/wheat/flour soft paste....J<br>ফল যেমন: কলা/পেঁপে/আম Banana/Papaya/mango....................K<br>ভাত/রুটি Rice/bread....................L<br>ডাল Lentil/pulse/dal....................M<br>মিষ্টি Sweet....................N<br>অন্যান্য Other________________.X<br>নির্দিষ্ট করুন	

Section H: Postnatal Care

This Section contains some information regarding care of the mother during her post natal period

ডেলিভারীর পর মায়ের সেবা
(Postnatal Care for Mothers)

No	Questions and filters	Options and coding category	Skip
H01	ডেলিভারীর পর, মা এবং শিশু অসুস্থ না হলেও তাদের ডাক্তারী বা মেডিকেল চেক আপ করা উচিত বলে আপনি কি মনে করেন?<br>Is there any need to have medical check-up of the mother and the baby after delivery even if the mother and the baby are not sick?	হ্যাঁ Yes....................1<br>না No....................2<br>জানি না/ মনে নাই Don't know/Can't remember....................9	 → H05<br>→ H05
H02	ডেলিভারীর পর, মা এবং শিশু অসুস্থ না হলেও তাদের প্রথম কবে (ডেলিভারীর কতদিন পর) ডাক্তারী বা মেডিকেল চেক আপ করা উচিত বলে আপনি মনে করেন?<br>Do you know when the first PNC visit is needed?	দিন পর Days after ....|___|___| জানি না/মনে নাই DK/can't remember....................99	
H03	আপনি বললেন, ডেলিভারীর পর মেডিকেল চেক আপ করা উচিত। কার কাছ থেকে এই মেডিকেল চেক আপ করানো উচিত?<br>*[মহিলাকে জিজ্ঞেস করুন]* আরও কেউ? *[মহিলার নিজে থেকে দেয়া সবগুলো উত্তরই বৃত্তায়িত করুন । উত্তরগুলো পড়ে শুনাবেন না। একাধিক উত্তর হতে পারে।]*<br>*[যদি মহিলার উত্তর সি,এস,বি,এ (Code E) বা MNCS প্রমোটর (Code H) হয়, তাহলে তাদের নাম লিখুন।]*<br>**Code :E** নাম Name: ________________<br>**Code :H** নাম Name: ________________<br>From whom should a mother and newborn get postnatal check-up?<br>*[don't prompt]*	**দক্ষ/প্রশিক্ষণ প্রাপ্ত স্বাস্থ্য কর্মী (Medically trained)**<br>পাশ করা ডাক্তার (MBBS doctor)....................A<br>নার্স/ধাত্রী (Nurse/midwife)....................B<br>প্যারামেডিক/মেডিকেল এসিসটেন্ট/সাকমো (Paramedic/MA/SACMO)....................C<br>পরিবার কল্যাণ পরিদর্শক (FWV)....................D<br>সি,এস,বি,এ (CSBA)....................E<br>**অন্যান্য স্বাস্থ্য কর্মী (Other health worker)**<br>স্বাস্থ্য সহকারী/ পরিবার কল্যান সহকারী (HA /FWA)....................F<br>পুষ্টি কর্মী (CNP)....................G<br>MNCS প্রমোটর (MNCS Promoter)....................H<br>অন্যান্য কমিউনিটি স্বাস্থ্য কর্মী - এনজিও কর্মী, স্বেচ্ছাসেবী (Other CHWs, NGO worker, volunteer)....................I<br>**অন্যান্য (Other)**<br>প্রশিক্ষণ প্রাপ্ত টিবিএ (প্রশিক্ষণ প্রাপ্ত ধন্নী, চাউনী, দাই) (TTBA)....................J<br>প্রশিক্ষণহীন টিবিএ (ধন্নী, চাউনী, দাই) TBA(Dai/Dhorni/Chauni)....................K<br>হোমিওপ্যাথ/হোমিওপ্যাথ ঔষধের দোকান (Homeopath/Homeopath drug store)....................L<br>আয়ুর্বেদিক চিকিৎসক / আয়ুর্বেদিক ঔষধের দোকান /হেকিম/কবিরাজ (Ayurved/ Ayurvedic drug store /Hekim/Kabiraj)....................M<br>গ্রাম ডাক্তার (Village doctor)....................N<br>এলোপ্যাথী ঔষধের দোকান (Allopath drug store)....................O<br>ইমাম/ঝাড় ফুক/ওঝা (Spiritual healer)....................P<br>পরিবারের অন্যান্য সদস্য/আত্মীয়/ প্রতিবেশী/বন্ধু Family/relative/Neighbor/friend....................Q<br>অন্যান্য Others ________________..X<br>(নির্দিষ্ট করুন)<br>জানি না/মনে নাই Don't know/can't remember....................Z	
H04	আপনি বললেন, ডেলিভারীর পর মেডিকেল চেক আপ করা উচিত। আপনি এই তথ্য কোথা থেকে বা কার কাছ থেকে জেনেছেন?<br>*[মহিলাকে জিজ্ঞেস করুন]* আরও কোথাও? *[মহিলার নিজে থেকে দেয়া সবগুলো উত্তরই বৃত্তায়িত করুন । উত্তরগুলো পড়ে শুনাবেন না। একাধিক উত্তর হতে পারে।]*<br>*[If mother mentions one or more of the above listed danger signs, Ask].*<br>where did you get this information/knowledge?	ডাক্তার/নার্স/ধাত্রী/প্যারামেডিক (Doctor/ Nurse/Midwife/ Paramedics)....................A<br>স্বাস্থ্য কেন্দ্র/হাসপাতাল (Health facility/ Clinic/ Hospital)....................B<br>কমিউনিটি স্বাস্থ্য কর্মী - স্বাস্থ্য সহকারী/পরিবার কল্যান সহকারী, পুষ্টি কর্মী, এনজিও কর্মী, MNCS প্রমোটর, স্বেচ্ছাসেবী (CHWs -HAS, FWAs, CNPs, MNCS promoter,NGO worker, volunteer)....................C<br>কমিউনিটি গ্রুপ মিটিং / মিটিং /সভা থেকে (Community meeting)....................D<br>রেডিও/টিভি (Radio /TV)....................E<br>সংবাদপত্র/খবরের কাগজ/বইপত্র (Newspaper/Books)....................F<br>পোষ্টার / কোন ছাপানো কাগজ (Poster/Leaflet)....................G<br>পরিবারের অন্যান্য সদস্য/আত্মীয়/প্রতিবেশী/বন্ধু Family/relatives/Neighbor/friend....................H<br>অন্যান্য Others________________...X<br>(নির্দিষ্ট করুন)<br>মনে নাই Can't remember....................Z	

No	Questions and filters	Options and coding category	Skip
H05	আপনার সর্বশেষ ডেলিভারীর পর আপনার নিজের মেডিকেল চেক-আপের জন্য আপনি কি কাউকে দেখিয়েছিলেন?<br>After the birth of NAME did you visit any health worker for PNC?	হ্যাঁ Yes....1<br>না No....2	 →H10
H06	আপনার সর্বশেষ ডেলিভারীর পর নিজের মেডিকেল চেক-আপের জন্য আপনি কাকে দেখিয়েছিলেন?<br><br>*[মহিলাকে জিজ্ঞেস করুন]* আরও কেউ? *[মহিলার নিজে থেকে দেয়া সবগুলো উত্তরই বৃত্তায়িত করুন । উত্তরগুলো পড়ে শুনাবেন না। একাধিক উত্তর হতে পারে।]*<br><br>*[যদি মহিলার উত্তর সি,এস,বি,এ (Code E) বা MNCS প্রমোটর (Code H) হয়, তাহলে তাদের নাম লিখুন।]*<br><br>**Code :E** নাম Name: ____________<br><br>**Code :H** নাম Name: ____________<br><br>To whom you went for PNC and how many times? Do not read out the answers. ASK: Anything else? Circle and write down all the answers	**<u>দক্ষ/প্রশিক্ষণ প্রাপ্ত স্বাস্থ্য কর্মী (Medically trained)</u>**<br>পাশ করা ডাক্তার (MBBS doctor)....A<br>নার্স/ধাত্রী (Nurse/midwife)....B<br>প্যারামেডিক/মেডিকেল এসিসটেন্ট/সাকমো (Paramedic/MA/SACMO)....C<br>পরিবার কল্যাণ পরিদর্শক (FWV)....D<br>সি,এস,বি,এ (CSBA)....E<br>**<u>অন্যান্য স্বাস্থ্য কর্মী (Other health worker)</u>**<br>স্বাস্থ্য সহকারী/ পরিবার কল্যান সহকারী (HA /FWA)....F<br>পুষ্টি কর্মী (CNP)....G<br>MNCS প্রমোটর (MNCS Promoter)....H<br>অন্যান্য কমিউনিটি স্বাস্থ্য কর্মী - এনজিও কর্মী, স্বেচ্ছাসেবী (Other CHWs, NGO worker, volunteer)....I<br>**<u>অন্যান্য (Other)</u>**<br>প্রশিক্ষণ প্রাপ্ত টিবিএ (প্রশিক্ষণ প্রাপ্ত ধন্নী, চাউনী, দাই) (TTBA)....J<br>প্রশিক্ষণহীন টিবিএ (ধন্নী, চাউনী, দাই) TBA(Dai/Dhorni/Chauni)....K<br>হোমিওপ্যাথ/হোমিওপ্যাথ ঔষধের দোকান (Homeopath/Homeopath drug store)....L<br>আয়ুর্বেদিক চিকিৎসক / আয়ুর্বেদিক ঔষধের দোকান /হেকিম/কবিরাজ (Ayurved/ Ayurvedic drug store /Hekim/Kabiraj)....M<br>গ্রাম ডাক্তার (Village doctor)....N<br>এলোপ্যাথী ঔষধের দোকান (Allopath drug store)....O<br>ইমাম/ঝাড় ফুক/ওঝা (Spiritual healer)....P<br>পরিবারের অন্যান্য সদস্য/আত্মীয়/ প্রতিবেশী/বন্ধু Family/relative/Neighbor/friend....Q<br>অন্যান্য Others ____________..X<br>(নির্দিষ্ট করুন)<br>জানি না/মনে নাই Don't know/can't remember....Z	

<table>
<tr><th>No</th><th>Questions and filters</th><th>Options and coding category</th><th>Skip</th></tr>
<tr><td>H07</td><td>আপনার সর্বশেষ ডেলিভারীর পর নিজের মেডিকেল চেক-আপের জন্য আপনি কোথায় দেখিয়েছিলেন?

[মহিলাকে জিজ্ঞেস করুন] আরও কোথাও? *[মহিলার নিজে থেকে দেয়া সবগুলো উত্তরই বৃত্তায়িত করুন । উত্তরগুলো পড়ে শুনাবেন না। একাধিক উত্তর হতে পারে।]*

[মহিলা যেখান থেকে সেবা পেয়েছেন, সেই স্বাস্থ্যকেন্দ্রের নাম লিখুন। যদি একাধিক জায়গা থেকে সেবা নিয়ে থাকেন, তাহলে সবগুলো জায়গারই নাম এবং কোড লিখুন।]

Code :|____| নাম Name: ______________________________

Code :|____| নাম Name: ______________________________

Code :|____| নাম Name: ______________________________

From where did you receive Post natal Care? Do not readout the answers. Ask anywhere else?Record all the answers</td><td>**বাড়ী (Home)**
নিজ বাড়ী, স্বামী/শ্বশুড় বাড়ী (Own home, husband/father in laws house).........A
বাবার বাড়ী (My natal home) ....B
অন্য কোন বাড়ী (Others)____C
(নির্দিষ্ট করুন)
সরকারী স্বাস্থ্য কেন্দ্র (Govt Health center)
মেডিকেল কলেজ হাসপাতাল (Medical College Hospital) ....D
জেলা/সদর হাসপাতাল (District /Sadar Hospital) ....E
মা ও শিশু স্বাস্থ্য কেন্দ্র (MCWC)..F
উপজেলা স্বাস্থ্য কমপ্লেক্স (UHC) ....G
ইউনিয়ন স্বাস্থ্য ও পরিবার কল্যাণ কেন্দ্র / সাব সেন্টার/আরডি (FWC/SC/RD) H
কমিউনিটি ক্লিনিক (Community clinic)...I
সেটেলাইট ক্লিনিক/ ইপিআই কেন্দ্র (Satellite clinic/EPI centre)...................J
অন্যান্য সরকারী স্বাস্থ্য কেন্দ্র (Other Govt Health facility) ....K
বেসরকারী স্বাস্থ্য কেন্দ্র (Non Govt Health center)
এনজিও হাসপাতাল (NGO hospital)..L
এনজিও স্থায়ী স্বাস্থ্য কেন্দ্র (NGO static health centre) ....M
এনজিও সেটেলাইট ক্লিনিক (NGO satellite clinic).................................N
পুষ্টি কেন্দ্র (NNP centre)...O
অন্যান্য বেসরকারী স্বাস্থ্য কেন্দ্র (Other NGO Health facility)......................P
প্রাইভেট (Private Health sector)
হাসপাতাল/ ক্লিনিক (Hospital/clinic)..Q
স্বাস্থ্য কেন্দ্র /ডিসপেনসারী (Health centre/Dispensary) ....R
এমবিবিএস ডাক্তারের চেম্বার (MBBS doctor's chamber) ....S
গ্রাম ডাক্তারের চেম্বার (Village doctor's chamber) ....T
প্যারামেডিক/মেডিকেল এসিসটেন্ট/সাকমোর চেম্বার (Paramedic/MA/SACMO chamber) U
এলোপ্যাথী ঔষধের দোকান (Allopath drug store) V
অন্যান্য প্রাইভেট_স্বাস্থ্য কেন্দ্র (Other private Health facility.......................W
অন্যান্য (Others) ____ ..X
(নির্দিষ্ট করুন)</td><td></td></tr>
<tr><td>H08</td><td>*[Question H06 দেখুন, code A থেকে I এর যেকোন একটি বা একাধিক বৃত্তায়িত থাকলে কোডগুলো এখানে লিখুন: _____ , _____, _____ এবার কোড/কোডগুলো দেখে দেখে মহিলাকে প্রশ্ন করুন ঃ]*

আপনি বললেন যে, আপনি ----, -----, ---- এর কাছ থেকে ডেলিভারীর পর মেডিকেল চেক-আপ করেছিলেন। এখন আমাকে বলুন যে,
আপনি এর/এদের ভিতর যার কাছ থেকে প্রথম মেডিকেল চেক-আপ করেছিলেন, তার কাছ থেকে ডেলিভারীর কতদিন পর প্রথম মেডিকেল চেক-আপ করিয়েছিলেন? এবং
আপনি এর/এদের কাছে ডেলিভারীর 7 দিনের ভিতর মোট কতবার মেডিকেল চেক-আপ করিয়েছিলেন?

[মহিলা যদি code A থেকে I এর ভিতর একাধিক স্বাস্থ্যকর্মীর থেকে সেবা গ্রহন করে থাকেন তাহলে তাদের সবার কথাই বলুন এবং তাদের ভিতর থেকে যার কাছে সর্বপ্রথম সেবা গ্রহন করেছিলেন তার কাছে ডেলিভারীর কতদিন পর প্রথম সেবা গ্রহন করেছিলেন সেটি এখানে লিখুন, এবং তাদের সবার কাছ থেকে ডেলিভারীর পর ১ম ৭ দিনে মোট কতবার সেবা গ্রহন করেছিলেন তা লিখুন।]

How many days after delivery did you visit a health worker (code A to I) for the first Postnatal Care for yourself? In the first week after the delivery how many times in total have you seek care from health worker(s) (code A to I)?</td><td>দিন পর Days after .... \|___\|___\|

জানি না/মনে নাই DK/can't remember 96

বার No. Of times ....\|___\|
একবারও আসেনি Didn't came in the 1st week 97
জানি না/ মনে নাই Don't know/don't remember....................................98

প্রযোজ্য নয় Not applicable ....99</td><td></td></tr>
</table>

No	Questions and filters	Options and coding category	Skip
H09	ডেলিভারীর পর আপনি যাদের কাছে মেডিকেল চেক-আপ করিয়েছিলেন, তাদের কাছ থেকে আপনি আপনার স্বাস্থ্যের ব্যাপারে কি কি উপদেশ/পরামর্শ বা সেবা পেয়েছিলেন?<br>*[মহিলাকে জিজ্ঞেস করুন]* আরও কিছু? *[মহিলার নিজে থেকে দেয়া সবগুলো উত্তরই বৃত্তায়িত করুন । উত্তরগুলো পড়ে শুনাবেন না। একাধিক উত্তর হতে পারে।]*<br>What sort of advices have you received from the person regarding your care after the delivery?	মায়ের স্বাস্থ্য পরীক্ষা করানোর কথা / পরামর্শ দিয়েছিলেন Asked for health chek up for the mother ....A<br>মায়ের বিপদের লক্ষণসমূহ সম্পর্কে পরামর্শ দিয়েছে Counseled on danger signs for mother ....B<br>বিপদের লক্ষণ থাকলে কোথায় যেতে হবে সে সম্পর্কে পরামর্শ দিয়েছে Counseled on where to go for complications ....C<br>সঠিক খাদ্যগ্রহন ও আয়রন-ফলিক এসিড গ্রহন সম্পর্কে পরামর্শ দিয়েছে Counseled on proper diet, routine iron and folic acid ....D<br>ভিটামিন-এ গ্রহন সম্পর্কে পরামর্শ দিয়েছে Counseled on vitamin-A ....E<br>পরিবার পরিকল্পনা পদ্ধতি সম্পর্কে পরামর্শ দিয়েছে about family planning ....F<br>মহিলার স্বাস্থ্য পরীক্ষা করেছে Examined the woman ....G<br>অসুস্থতার জন্য মহিলাকে স্বাস্থ্য কেন্দ্রে রেফার করেছে Referred the woman to the health center for illness ....H<br>অন্যান্য Others ________________ X<br>নির্দিষ্ট করুন<br>কোন উপদেশ পাই নি No advice received ....Z	Skip to H11 for all
H10	ডেলিভারীর পর আপনার নিজের মেডিকেল চেক-আপের জন্য আপনি কাউকে কেন দেখান নি?<br>*[মহিলাকে জিজ্ঞেস করুন]* আরও কিছু? *[মহিলার নিজে থেকে দেয়া সবগুলো উত্তরই বৃত্তায়িত করুন । উত্তরগুলো পড়ে শুনাবেন না। একাধিক উত্তর হতে পারে।]*<br>If the women didn't seek PNC then ask, why she didn't seek PNC from a health care provider? Do not readout the answers. Ask anyone else? Record all the answers	সেবার প্রয়োজন আছে বলে মনে হয় নি/সেবার প্রয়োজন নেই Didn't think it was necessary to seek care ....A<br>জানতাম না কোথায় যেতে হবে Not known where to go ....B<br>অনেক খরচ/ টাকা পয়সা ছিল না Too costly/ Lack of money ....C<br>স্বাস্থ্য কেন্দ্র বাসা হতে অনেক দুরে Too far from house ....D<br>যানবাহনের সমস্যা Transport problem ....E<br>সাথে যাবার মত কেউ ছিল না No one accompanied ....F<br>স্বাস্থ্যকেন্দ্রে যাবার মত সময় ছিল না Not enough time to go ....G<br>পরিবার আমাকে যেতে দেন নি Family didn't allow me to go ....H<br>ধর্মে মানা/বাধা Religeous bar ....I<br>স্বাস্থ্যকেন্দ্রের সেবাদানের সময় সীমা সুবিধাজনক নয় Service hr inconvenient ...J<br>স্বাস্থ্যকেন্দ্র বন্ধ ছিল/কোন স্বাস্থ্যকর্মী ছিলেন না HF found closed/nobody there....K<br>স্বাস্থ্যকেন্দ্রে সেবা অনুন্নত মানের Poor quality of services at facility ....L<br>স্বাস্থ্যকেন্দ্রে নিম্নমানের এবং অদক্ষ সেবাপ্রদানকারী Poor quality & staffs at HF ..M<br>স্বাস্থ্যকেন্দ্রে পর্দার অভাব Lack of privacy ....N<br>স্বাস্থ্যকেন্দ্রের সেবাদানকারীদের ব্যবহার খারাপ Unpleasant behavior at center ...O<br>স্বাস্থ্যকেন্দ্রের অনেকক্ষন বসে থাকতে হয় সেবা পাবার জন্য Long queue at HF .P<br>স্বাস্থ্যকেন্দ্রের ঔষধ পত্র পাওয়া যায়না Inadequate drugs at the health center ....Q<br>অন্যান্য Others ________________ ..X	
H11	আপনার সর্বশেষ ডেলিভারীর পর কোন সমস্যা / জটিলতা হয়েছিল কি?<br>*[প্রত্যেকটি সমস্যা পড়ে শোনান]*<br>After the birth of (Name) did you have any of the following problem/ complication? Ask all the questions of the list and take the answer.	YES NO<br>মাথা ব্যথা/ঝাপসা দেখা/ উচ্চরক্তচাপ Headache/blurry vision/high BP ....1 ......2<br>পা/মুখ ফুলে গিয়েছিল Oedema/pre-eclampsia ....1 ......2<br>খিচুঁনী হয়েছিল Convulsion/eclampsia ....1 ......2<br>অতিরিক্ত জ্বর High fever ....1 ......2<br>যোনীপথে অতিরিক্ত রক্তস্রাব Excessive vaginal bleeding ....1 ......2<br>যোনীপথে দূর্গন্ধযুক্ত স্রাব Foul smelling vaginal discharge ....1 ......2<br>তলপেটে তীব্র ব্যথা Severe lower abdominal pain ....1 ......2<br>শ্বাস নিতে কষ্ট, দূর্বলতা Breathlessness, tiredness ....1 ......2<br>বুক ধরফরানি palpitation....1 ......2<br>ফিট/খিঁচুনী Fits and convulsion ....1 ......2<br>অন্য কোন সমস্যা others ________ ....1 ......2<br>নির্দিষ্ট করুন	
H12	*[প্রশ্ন H11 দেখুন এবং সঠিক কোড বৃত্তায়িত করুন]*<br>Interviewer: Check Question H11 and circle appropriate code	এক বা একাধিক কোড 1 বৃত্তায়িত One or more codes circled 1 ....1<br>সবগুলো কোড 2 বৃত্তায়িত All codes circled 2 2	→H21
H13	আপনার মতে সমস্যা/ জটিলতা টি কি খুব মারাত্নক নাকি মোটামুটি নাকি সামান্য ছিল?<br>From your opinion, was this problem/complication severe or mild to moderate?	সামান্য ছিল Mild ....1<br>মোটামুটি ছিল Moderate ....2<br>মারাত্মক ছিল Severe ....3<br>অন্যান্য Other : ________________ ..7<br>জানেন না Doesn't know 9	

No	Questions and filters	Options and coding category	Skip
H14	এই সমস্যা/জটিলতার জন্য আপনি কি কাউকে দেখিয়েছিলেন বা কারও সাহায্য নিয়েছিলেন?<br>Did you seek any sort of treatment for this problem/complication?	হ্যাঁ Yes....................1<br>না No....................2<br>জানি না/ মনে নাই Don't know/Can't remember....................9	 →H20<br>→H20
H15	এই সমস্যা/জটিলতার জন্য আপনি কাকে দেখিয়েছিলেন বা কার সাহায্য নিয়েছিলেন?<br><br>*[মহিলাকে জিজ্ঞেস করুন]* আরও কেউ? *[মহিলার নিজে থেকে দেয়া সবগুলো উত্তরই বৃত্তায়িত করুন । উত্তরগুলো পড়ে শুনাবেন না । একাধিক উত্তর হতে পারে ।]*<br><br>*[যদি মহিলার উত্তর সি,এস,বি,এ (Code E) বা MNCS প্রমোটর (Code H) হয়, তাহলে তাদের নাম লিখুন ।]*<br><br>**Code :E** নাম Name: ________________<br><br>**Code :H** নাম Name: ________________<br><br>From whom did you seek treatment for this problem/complication? Do not read out the answers. Ask: Anything else? Circle all the answers	**দক্ষ/প্রশিক্ষণ প্রাপ্ত স্বাস্থ্য কর্মী (Medically trained)**<br>পাশ করা ডাক্তার (MBBS doctor)....................A<br>নার্স/ধাত্রী (Nurse/midwife)....................B<br>প্যারামেডিক/মেডিকেল এসিসটেন্ট/সাকমো (Paramedic/MA/SACMO)....................C<br>পরিবার কল্যাণ পরিদর্শক (FWV)....................D<br>সি,এস,বি,এ (CSBA)....................E<br>**অন্যান্য স্বাস্থ্য কর্মী (Other health worker)**<br>স্বাস্থ্য সহকারী/ পরিবার কল্যান সহকারী (HA /FWA)....................F<br>পুষ্টি কর্মী (CNP)....................G<br>MNCS প্রমোটর (MNCS Promoter)....................H<br>অন্যান্য কমিউনিটি স্বাস্থ্য কর্মী - এনজিও কর্মী, স্বেচ্ছাসেবী (Other CHWs, NGO worker, volunteer)....................I<br>**অন্যান্য (Other)**<br>প্রশিক্ষণ প্রাপ্ত টিবিএ (প্রশিক্ষণ প্রাপ্ত ধন্নী, চাউনী, দাই) (TTBA)....................J<br>প্রশিক্ষণহীন টিবিএ (ধন্নী, চাউনী, দাই) TBA(Dai/Dhorni/Chauni)....................K<br>হোমিওপ্যাথ/হোমিওপ্যাথ ঔষধের দোকান (Homeopath/Homeopath drug store)....................L<br>আয়ুর্বেদিক চিকিৎসক / আয়ুর্বেদিক ঔষধের দোকান /হেকিম/কবিরাজ (Ayurved/ Ayurvedic drug store /Hekim/Kabiraj)....................M<br>গ্রাম ডাক্তার (Village doctor)....................N<br>এলোপ্যাথী ঔষধের দোকান (Allopath drug store)....................O<br>ইমাম/ঝাড় ফুক/ওঝা (Spiritual healer)....................P<br>পরিবারের অন্যান্য সদস্য/আত্মীয়/ প্রতিবেশী/বন্ধু Family/relative/Neighbor/friend....................Q<br>অন্যান্য Others ________________..X<br>(নির্দিষ্ট করুন)<br>জানি না/মনে নাই Don't know/can't remember....................Z	

No	Questions and filters	Options and coding category	Skip
H16	এই সমস্যা/জটিলতার জন্য আপনি কোথায় দেখিয়েছিলেন বা কোথায় সেবা পেয়েছিলেন?<br><br>*[মহিলাকে জিজ্ঞেস করুন]* আরও কোথাও? *[মহিলার নিজে থেকে দেয়া সবগুলো উত্তরই বৃত্তায়িত করুন । উত্তরগুলো পড়ে শুনাবেন না। একাধিক উত্তর হতে পারে।]*<br><br>*[মহিলা যেখান থেকে সেবা পেয়েছেন, সেই স্বাস্থ্যকেন্দ্রের নাম লিখুন। যদি একাধিক জায়গা থেকে সেবা নিয়ে থাকেন, তাহলে সবগুলো জায়গারই নাম এবং কোড লিখুন।]*<br><br>Code :|____| নাম Name: ________________________<br>Code :|____| নাম Name: ________________________<br>Code :|____| নাম Name: ________________________<br><br>Where did you go to seek care for this problem/complication? Do not read out the answers. Ask: Anything else? Circle all the answers	**<u>বাড়ী (Home)</u>**<br>নিজ বাড়ী, স্বামী/শ্বশুড় বাড়ী (Own home, husband/father in laws house)......... A<br>বাবার বাড়ী (My natal home) .... B<br>অন্য কোন বাড়ী (Others)____________ C<br>(নির্দিষ্ট করুন)<br>**<u>সরকারী স্বাস্থ্য কেন্দ্র (Govt Health center)</u>**<br>মেডিকেল কলেজ হাসপাতাল (Medical College Hospital) .... D<br>জেলা/সদর হাসপাতাল (District /Sadar Hospital) .... E<br>মা ও শিশু স্বাস্থ্য কেন্দ্র (MCWC)........ F<br>উপজেলা স্বাস্থ্য কমপ্লেক্স (UHC) .... G<br>ইউনিয়ন স্বাস্থ্য ও পরিবার কল্যাণ কেন্দ্র / সাব সেন্টার/আরডি (FWC/SC/RD) H<br>কমিউনিটি ক্লিনিক (Community clinic)........ I<br>সেটেলাইট ক্লিনিক/ ইপিআই কেন্দ্র (Satellite clinic/EPI centre)........ J<br>অন্যান্য সরকারী স্বাস্থ্য কেন্দ্র (Other Govt Health facility) .... K<br>**<u>বেসরকারী স্বাস্থ্য কেন্দ্র (Non Govt Health center)</u>**<br>এনজিও হাসপাতাল (NGO hospital)........ L<br>এনজিও স্থায়ী স্বাস্থ্য কেন্দ্র (NGO static health centre) .... M<br>এনজিও সেটেলাইট ক্লিনিক (NGO satellite clinic)........ N<br>পুষ্টি কেন্দ্র (NNP centre)........ O<br>অন্যান্য বেসরকারী স্বাস্থ্য কেন্দ্র (Other NGO Health facility)........ P<br>**<u>প্রাইভেট (Private Health sector)</u>**<br>হাসপাতাল/ ক্লিনিক (Hospital/clinic)........ Q<br>স্বাস্থ্য কেন্দ্র /ডিসপেনসারী (Health centre/Dispensary) .... R<br>এমবিবিএস ডাক্তারের চেম্বার (MBBS doctor's chamber) .... S<br>গ্রাম ডাক্তারের চেম্বার (Village doctor's chamber) .... T<br>প্যারামেডিক/মেডিকেল এসিসটেন্ট/সাকমোর চেম্বার (Paramedic/MA/SACMO chamber) U<br>এলোপ্যাথী ঔষধের দোকান (Allopath drug store) .... V<br>অন্যান্য প্রাইভেট_স্বাস্থ্য কেন্দ্র (Other private Health facility........ W<br>অন্যান্য (Others) ____________ .. X<br>(নির্দিষ্ট করুন)	
H17	এই সমস্যা/জটিলতার জন্য কাউকে দেখানোর ব্যাপারে কেউ কি আপনাকে পরামর্শ দিয়েছিল?<br>Did anyone advise you or refer you to seek care for your complication?	হ্যাঁ Yes........ 1<br>না No .... 2<br>জানি না/ মনে নাই Don't know/Can't remember 9	 →H21<br>→H21

No	Questions and filters	Options and coding category	Skip
H18	এই সমস্যা/জটিলতার জন্য কাউকে দেখানোর ব্যাপারে কে আপনাকে পরামর্শ দিয়েছিল?<br><br>*[[মহিলাকে জিজ্ঞেস করুন]* আরও কেউ? *[মহিলার নিজে থেকে দেয়া সবগুলো উত্তরই বৃত্তায়িত করুন । উত্তরগুলো পড়ে শুনাবেন না । একাধিক উত্তর হতে পারে ।]*<br><br>*[যদি মহিলার উত্তর সি,এস,বি,এ (Code E) বা MNCS প্রমোটর (Code H) হয়, তাহলে তাদের নাম লিখুন ।]*<br><br>**Code :E** নাম Name: ______________________________<br><br>**Code :H** নাম Name: ______________________________<br><br>Who had advised you or refered you to seek care for your complication? Do not read out the answers. Ask: Anything else? Circle all the answers	**দক্ষ/প্রশিক্ষণ প্রাপ্ত স্বাস্থ্য কর্মী (Medically trained)**<br>পাশ করা ডাক্তার (MBBS doctor) .... A<br>নার্স/ধাত্রী (Nurse/midwife) .... B<br>প্যারামেডিক/মেডিকেল এসিসটেন্ট/সাকমো (Paramedic/MA/SACMO) .... C<br>পরিবার কল্যাণ পরিদর্শক (FWV) .... D<br>সি,এস,বি,এ (CSBA) .... E<br>**অন্যান্য স্বাস্থ্য কর্মী (Other health worker)**<br>স্বাস্থ্য সহকারী/ পরিবার কল্যান সহকারী (HA /FWA) .... F<br>পুষ্টি কর্মী (CNP) .... G<br>MNCS প্রমোটর (MNCS Promoter) .... H<br>অন্যান্য কমিউনিটি স্বাস্থ্য কর্মী - এনজিও কর্মী, স্বেচ্ছাসেবী (Other CHWs, NGO worker, volunteer) .... I<br>**অন্যান্য (Other)**<br>প্রশিক্ষণ প্রাপ্ত টিবিএ (প্রশিক্ষণ প্রাপ্ত ধন্নী, চাউনী, দাই) (TTBA) .... J<br>প্রশিক্ষণহীন টিবিএ (ধন্নী, চাউনী, দাই) TBA(Dai/Dhorni/Chauni) .... K<br>হোমিওপ্যাথ/হোমিওপ্যাথ ঔষধের দোকান (Homeopath/Homeopath drug store) .... L<br>আয়ুর্বেদিক চিকিৎসক / আয়ুর্বেদিক ঔষধের দোকান /হেকিম/কবিরাজ (Ayurved/ Ayurvedic drug store /Hekim/Kabiraj) .... M<br>গ্রাম ডাক্তার (Village doctor) .... N<br>এলোপ্যাথী ঔষধের দোকান (Allopath drug store) .... O<br>ইমাম/ঝাড় ফুক/ওঝা (Spiritual healer) .... P<br>পরিবারের অন্যান্য সদস্য/আত্মীয়/ প্রতিবেশী/বন্ধু Family/relative/Neighbor/friend .... Q<br>অন্যান্য Others ______________________ .. X<br>(নির্দিষ্ট করুন)<br>জানি না/মনে নাই Don't know/can't remember .... Z	
H19	এই সমস্যা/জটিলতার জন্য কাউকে দেখানোর ব্যাপারে যিনি আপনাকে পরামর্শ করেছিলেন, তিনি কি আপনাকে কোন কাগজ (রেফারেল স্লিপ) দিয়েছিলেন? (রেফারেল স্লিপের একটি নমুনা দেখান)<br>Did the person who had referred you issue you a referral slip to show it to the health facility/carer ? (show the referral slip to the respondent)	হ্যাঁ Yes .... 1<br>না No .... 2<br>জানি না/ মনে নাই Don't know/Can't remember 9	→ H21<br>→ H21<br>→ H21
H20	আপনি কেন এই সমস্যা/জটিলতার জন্য কাউকে দেখান বা সেবা নেন নি?<br><br>*[মহিলাকে জিজ্ঞেস করুন]* আরও কোন কারন? *[মহিলার নিজে থেকে দেয়া সবগুলো উত্তরই বৃত্তায়িত করুন । উত্তরগুলো পড়ে শুনাবেন না । একাধিক উত্তর হতে পারে ।]*<br><br>Why did you not seek care for this complication? Do not read out the answers. Ask: Anything else? Circle all the answers	সেবার প্রয়োজন আছে বলে মনে হয় নি/সেবার প্রয়োজন নেই Didn't think it was necessary to seek care .... A<br>জানতাম না কোথায় যেতে হবে Not known where to go .... B<br>অনেক খরচ/ টাকা পয়সা ছিল না Too costly/ Lack of money .... C<br>স্বাস্থ্য কেন্দ্র বাসা হতে অনেক দুরে Too far from house .... D<br>যানবাহনের সমস্যা Transport problem .... E<br>সাথে যাবার মত কেউ ছিল না No one accompanied .... F<br>স্বাস্থ্যকেন্দ্রে যাবার মত সময় ছিল না Not enough time to go .... G<br>পরিবার আমাকে যেতে দেন নি Family didn't allow me to go .... H<br>ধর্মে মানা/বাধা Religeous bar .... I<br>স্বাস্থ্যকেন্দ্রের সেবাদানের সময় সীমা সুবিধাজনক নয় Service hr inconvenient ... J<br>স্বাস্থ্যকেন্দ্র বন্ধ ছিল/কোন স্বাস্থ্যকর্মী ছিলেন না HF found closed/nobody there ..... K<br>স্বাস্থ্যকেন্দ্রে সেবা অনুন্নত মানের Poor quality of services at facility .... L<br>স্বাস্থ্যকেন্দ্রে নিম্নমানের এবং অদক্ষ সেবাপ্রদানকারী Poor quality & staffs at HF .. M<br>স্বাস্থ্যকেন্দ্রে পর্দার অভাব Lack of privacy .... N<br>স্বাস্থ্যকেন্দ্রের সেবাদানকারীদের ব্যবহার খারাপ Unpleasant behavior at center ... O<br>স্বাস্থ্যকেন্দ্রের অনেকক্ষন বসে থাকতে হয় সেবা পাবার জন্য Long queue at HF . P<br>স্বাস্থ্যকেন্দ্রের ঔষধ পত্র পাওয়া যায়না Inadequate drugs at the health center .... Q<br>অন্যান্য Others ______________________ .. X	

No	Questions and filters	Options and coding category	Skip
H21	একজন মহিলার গর্ভকালীন সময়ে, ডেলিভারীর সময় বা ডেলিভারীর পর কি কি সমস্যা হতে পারে, আমাকে কি বলবেন?<br><br>*[মহিলাকে জিজ্ঞেস করুন]* আরও কিছু? *[মহিলার নিজে থেকে দেয়া সবগুলো উত্তরই বৃত্তায়িত করুন । উত্তরগুলো পড়ে শুনাবেন না । একাধিক উত্তর হতে পারে ।]*<br><br>Please tell me what are the complications that may occur during pregnancy period, delivery time and after delivery that need medical support?	দীর্ঘ প্রসব ব্যথা (Long Labor) .... A<br>অতিরিক্ত রক্তস্রাব (Excessive vaginal bleeding) .... B<br>অতিরিক্ত জ্বর (High fever) .... C<br>খিচুনী (Convulsion) .... D<br>হাত/পা বের হওয়া (Babies hands and feet came first during delivery) .... E<br>ফুল না পরা (Retained Placenta) .... F<br>তীব্র মাথা ব্যথা (Severe Headache) .... G<br>প্রি-এক্লামসিয়া (Pre-Eclampsia) .... H<br>বাঁধাগ্রস্থ প্রসব (Obstructed labour) .... I<br>টিটেনাস (Tetanus) .... J<br>অন্যান্য (Others) ________ ..X<br>(নির্দিষ্ট করুন)<br>জানি না (Don't know) .... Z	
H22	গর্ভকালীন সময়ে, ডেলিভারীর সময় বা ডেলিভারীর পর বিভিন্ন সমস্যার/জটিলতার জন্য কোথায় কোথায় সেবা বা চিকিৎসা নিতে যাওয়া যেতে পারে, সেটা বলবেন কি?<br><br>*[মহিলাকে জিজ্ঞেস করুন]* আরও কিছু? *[মহিলার নিজে থেকে দেয়া সবগুলো উত্তরই বৃত্তায়িত করুন । উত্তরগুলো পড়ে শুনাবেন না । একাধিক উত্তর হতে পারে ।]*<br><br>Can you tell me from where you can sought care for medical check-up for complication during pregnancy, deliveryand after delivery?	**সরকারী স্বাস্থ্য কেন্দ্র (Govt Health center)**<br>মেডিকেল কলেজ হাসপাতাল (Medical College Hospital) .... A<br>জেলা/সদর হাসপাতাল (District /Sadar Hospital) .... B<br>মা ও শিশু স্বাস্থ্য কেন্দ্র (MCWC) .... C<br>উপজেলা স্বাস্থ্য কমপ্লেক্স (UHC) .... D<br>ইউনিয়ন স্বাস্থ্য ও পরিবার কল্যাণ কেন্দ্র/সাব সেন্টার/আরডি (FWC/SC/RD)... E<br>কমিউনিটি ক্লিনিক (Community clinic) .... F<br>সেটেলাইট ক্লিনিক/ ইপিআই কেন্দ্র (Satellite clinic/EPI centre) .... G<br>অন্যান্য সরকারী স্বাস্থ্য কেন্দ্র (Other Govt Health facility) .... H<br>**বেসরকারী স্বাস্থ্য কেন্দ্র (Non Govt Health center)**<br>এনজিও হাসপাতাল (NGO hospital) .... I<br>এনজিও স্থায়ী স্বাস্থ্য কেন্দ্র (NGO static health centre) .... J<br>এনজিও সেটেলাইট ক্লিনিক (NGO satellite clinic) .... K<br>পুষ্টি কেন্দ্র (NNP centre) .... L<br>অন্যান্য বেসরকারী স্বাস্থ্য কেন্দ্র (Other NGO Health facility) .... M<br>**প্রাইভেট (Private Health sector)**<br>হাসপাতাল/ ক্লিনিক (Hospital/clinic) .... N<br>স্বাস্থ্য কেন্দ্র /ডিসপেনসারী (Health centre/Dispensary) .... O<br>এমবিবিএস ডাক্তারের চেম্বার (MBBS doctor's chamber) .... P<br>গ্রাম ডাক্তারের চেম্বার (Village doctor's chamber) .... Q<br>প্যারামেডিক/মেডিকেল এসিসটেন্ট/সাকমোর চেম্বার (Paramedic/ MA/SACMO chamber) R<br>এলোপ্যাথী ঔষধের দোকান (Allopath drug store) .... S<br>অন্যান্য প্রাইভেট_স্বাস্থ্য কেন্দ্র (Other private Health facility) .... T<br>অন্যান্য (Others) ________ X<br>(নির্দিষ্ট করুন)<br>কোথায় যেতে হবে, জানা নাই (Don't know) .... Z	
H23	*[প্রশ্ন নং H21 এবং H22 দেখুন। যদি মা একটিও সমস্যা/জটিলতার কথা বা/এবং তার জন্য কোথায় সেবা বা চিকিৎসা নিতে যাওয়া যায় তার কথা বলতে পারেন, তাহলে মহিলাকে জিজ্ঞেস করুন;]*<br>আপনি বললেন যে, -----, ---- সমস্যা/জটিলতা হলেও হতে পারে। এবং সেবা বা চিকিৎসার জন্য -----, ----- জায়গায় যাওয়া যেতে পারে - এখন আমাকে বলুন তো, আপনি এই তথ্য কোথা থেকে বা কার কাছ থেকে জেনেছেন?<br>*[মহিলাকে জিজ্ঞেস করুন]* আরও কিছু? *[মহিলার নিজে থেকে দেয়া সবগুলো উত্তরই বৃত্তায়িত করুন । উত্তরগুলো পড়ে শুনাবেন না । একাধিক উত্তর হতে পারে ।]*<br>If mother mentions one or more of the above listed danger signs, Ask. from where /who provided you with the	ডাক্তার/নার্স/ধাত্রী/প্যারামেডিক (Doctor/ Nurse/Midwife/ Paramedics) .... A<br>স্বাস্থ্য কেন্দ্র/হাসপাতাল (Health facility/ Clinic/ Hospital) .... B<br>কমিউনিটি স্বাস্থ্য কর্মী - স্বাস্থ্য সহকারী/পরিবার কল্যান সহকারী, পুষ্টি কর্মী, এনজিও কর্মী, MNCS প্রমোটর, স্বেচ্ছাসেবী (CHWs -HAS, FWAs, CNPs, MNCS promoter,NGO worker, volunteer) .... C<br>কমিউনিটি গ্রুপ মিটিং / মিটিং /সভা থেকে (Community meeting) .... D<br>রেডিও/টিভি (Radio /TV) .... E<br>সংবাদপত্র/খবরের কাগজ/বইপত্র (Newspaper/Books) .... F<br>পোষ্টার / কোন ছাপানো কাগজ (Poster/Leaflet) .... G<br>পরিবারের অন্যান্য সদস্য/আত্মীয়/প্রতিবেশী/বন্ধু Family/relatives/Neighbor/friend ..... H<br>অন্যান্য Others________ ...X<br>(নির্দিষ্ট করুন)<br>মনে নাই Can't remember .... Z	

ডেলিভারীর পর শিশুর সেবা

Care of the Newborn

No	Questions and filters	Options and coding category	Skip
H24	*[প্রশ্ন নং D15এবং D16 দেখুন]*<br>*[মহিলার ১লা জুন, 2011 বা এর পরে হওয়া সর্বশেষ গর্ভের ফলাফল কি ছিল?]*<br>*[Check Q D15 and D16 for the answer. What was the outcome of your most recent pregnancy since 1 June 2011].*	জীবিত জন্ম Live Birth ....1<br>মৃত জন্ম Still Birth ....2<br>গর্ভপাত/গর্ভনষ্ট Abortion 3	 →H42<br>→I00a
H25	ডেলিভারীর পর <নাম>-কে কি কাউকে দেখিয়েছিলেন বা স্বাস্থ্য পরীক্ষা করিয়েছিলেন?<br>After the birth of NAME did you visit anyone for PNC?	হ্যাঁ Yes ....1<br>না No 2	 →H31
H26	ডেলিভারীর পর <নাম>-কে কাকে দেখিয়েছিলেন বা কে স্বাস্থ্য পরীক্ষা করেছিল?<br><br>*[মহিলাকে জিজ্ঞেস করুন]* আরও কেউ? *[মহিলার নিজে থেকে দেয়া সবগুলো উত্তরই বৃত্তায়িত করুন । উত্তরগুলো পড়ে শুনাবেন না। একাধিক উত্তর হতে পারে।]*<br><br>*[যদি মহিলার উত্তর সি,এস,বি,এ (Code E) বা MNCS প্রমোটর (Code H) হয়, তাহলে তাদের নাম লিখুন।]*<br><br>**Code :E** নাম Name: ____________________<br><br>**Code :H** নাম Name: ____________________<br><br>To whom you went for PNC and how many times? Do not read out the answers. ASK: Anything else? Circle and write down all the answers	**দক্ষ/প্রশিক্ষণ প্রাপ্ত স্বাস্থ্য কর্মী (Medically trained)**<br>পাশ করা ডাক্তার (MBBS doctor) ....A<br>নার্স/ধাত্রী (Nurse/midwife) ....B<br>প্যারামেডিক/মেডিকেল এসিসটেন্ট/সাকমো (Paramedic/MA/SACMO) ....C<br>পরিবার কল্যাণ পরিদর্শক (FWV) ....D<br>সি,এস,বি,এ (CSBA) ....E<br>**অন্যান্য স্বাস্থ্য কর্মী (Other health worker)**<br>স্বাস্থ্য সহকারী/ পরিবার কল্যান সহকারী (HA /FWA) ....F<br>পুষ্টি কর্মী (CNP) ....G<br>MNCS প্রমোটর (MNCS Promoter) ....H<br>অন্যান্য কমিউনিটি স্বাস্থ্য কর্মী - এনজিও কর্মী, স্বেচ্ছাসেবী (Other CHWs, NGO worker, volunteer) ....I<br>**অন্যান্য (Other)**<br>প্রশিক্ষণ প্রাপ্ত টিবিএ (প্রশিক্ষণ প্রাপ্ত ধন্নী, চাউনী, দাই) (TTBA) ....J<br>প্রশিক্ষণহীন টিবিএ (ধন্নী, চাউনী, দাই) TBA(Dai/Dhorni/Chauni) ....K<br>হোমিওপ্যাথ/হোমিওপ্যাথ ঔষধের দোকান (Homeopath/Homeopath drug store) ....L<br>আয়ুর্বেদিক চিকিৎসক / আয়ুর্বেদিক ঔষধের দোকান /হেকিম/কবিরাজ (Ayurved/ Ayurvedic drug store /Hekim/Kabiraj) ....M<br>গ্রাম ডাক্তার (Village doctor) ....N<br>এলোপ্যাথী ঔষধের দোকান (Allopath drug store) ....O<br>ইমাম/ঝাড় ফুক/ওঝা (Spiritual healer) ....P<br>পরিবারের অন্যান্য সদস্য/আত্মীয়/ প্রতিবেশী/বন্ধু Family/relative/Neighbor/friend ....Q<br>অন্যান্য Others ____________________ ..X<br>(নির্দিষ্ট করুন)<br>জানি না/মনে নাই Don't know/can't remember ....Z	

No	Questions and filters	Options and coding category	Skip
H27	ডেলিভারীর পর <নাম>-কে স্বাস্থ্য পরীক্ষার জন্য আপনি কোথায় দেখিয়েছিলেন বা স্বাস্থ্য পরীক্ষা করেছিলেন?<br><br>*[মহিলাকে জিজ্ঞেস করুন]* আরও কোথায়? *[মহিলার নিজে থেকে দেয়া সবগুলো উত্তরই বৃত্তায়িত করুন । উত্তরগুলো পড়ে শুনাবেন না । একাধিক উত্তর হতে পারে ।]*<br><br>*[মহিলা যেখান থেকে সেবা পেয়েছেন, সেই স্বাস্থ্যকেন্দ্রের নাম লিখুন । যদি একাধিক জায়গা থেকে সেবা নিয়ে থাকেন, তাহলে সবগুলো জায়গারই নাম এবং কোড লিখুন ।]*<br><br>Code :|____| নাম Name: _________________________<br>Code :|____| নাম Name: _________________________<br>Code :|____| নাম Name: _________________________<br><br>From where did you receive Post natal Care? Do not readout the answers. Ask anywhere else? Record all the answers	**বাড়ী (Home)**<br>নিজ বাড়ী, স্বামী/শ্বশুড় বাড়ী (Own home, husband/father in laws house)……… A<br>বাবার বাড়ী (My natal home) ……… B<br>অন্য কোন বাড়ী (Others)________________ C<br>(নির্দিষ্ট করুন)<br>**সরকারী স্বাস্থ্য কেন্দ্র (Govt Health center)**<br>মেডিকেল কলেজ হাসপাতাল (Medical College Hospital) ……… D<br>জেলা/সদর হাসপাতাল (District /Sadar Hospital) ……… E<br>মা ও শিশু স্বাস্থ্য কেন্দ্র (MCWC)……… F<br>উপজেলা স্বাস্থ্য কমপ্লেক্স (UHC) ……… G<br>ইউনিয়ন স্বাস্থ্য ও পরিবার কল্যাণ কেন্দ্র / সাব সেন্টার/আরডি (FWC/SC/RD) H<br>কমিউনিটি ক্লিনিক (Community clinic)……… I<br>সেটেলাইট ক্লিনিক/ ইপিআই কেন্দ্র (Satellite clinic/EPI centre)……… J<br>অন্যান্য সরকারী স্বাস্থ্য কেন্দ্র (Other Govt Health facility) ……… K<br>**বেসরকারী স্বাস্থ্য কেন্দ্র (Non Govt Health center)**<br>এনজিও হাসপাতাল (NGO hospital)……… L<br>এনজিও স্থায়ী স্বাস্থ্য কেন্দ্র (NGO static health centre) ……… M<br>এনজিও সেটেলাইট ক্লিনিক (NGO satellite clinic)……… N<br>পুষ্টি কেন্দ্র (NNP centre)……… O<br>অন্যান্য বেসরকারী স্বাস্থ্য কেন্দ্র (Other NGO Health facility)……… P<br>**প্রাইভেট (Private Health sector)**<br>হাসপাতাল/ ক্লিনিক (Hospital/clinic)……… Q<br>স্বাস্থ্য কেন্দ্র /ডিসপেনসারী (Health centre/Dispensary) ……… R<br>এমবিবিএস ডাক্তারের চেম্বার (MBBS doctor's chamber) ……… S<br>গ্রাম ডাক্তারের চেম্বার (Village doctor's chamber) ……… T<br>প্যারামেডিক/মেডিকেল এসিসটেন্ট/সাকমোর চেম্বার (Paramedic/MA/SACMO chamber) U<br>এলোপ্যাথী ঔষধের দোকান (Allopath drug store) ……… V<br>অন্যান্য প্রাইভেট_স্বাস্থ্য কেন্দ্র (Other private Health facility……… W<br>অন্যান্য (Others) ________________.. X<br>(নির্দিষ্ট করুন)	
H28	*[প্রশ্ন নং H27 চেক করুন । লক্ষ করুন - কোড নং A থেকে C এর কোনটি কি বৃত্তায়িত করা হয়েছে? যদি বৃত্তায়িত না থাকে, তাহলে মহিলাকে প্রশ্ন করুন:]*<br><br>ডেলিভারীর পর (নাম)-কে সেবা দিতে বা দেখতে বা স্বাস্থ্য পরীক্ষা করতে আপনার বাড়ীতে কি কেউ এসেছিল?<br><br>Check H26, Code A to C circled? if No then ask the mother After the delivery, did the child receive any care at HOME? If yes, correct the answer made in Q H25 and H26.	হ্যাঁ Yes……… 1<br>না No ……… 2<br><br>[উত্তর হ্যাঁ হলে, প্রশ্ন নং H26 এবং H27 আবারও করুন এবং প্রয়োজনীয় সংশোধন করুন ।]	→Correct H26 and H27

No	Questions and filters	Options and coding category	Skip
H29	[Question H26 দেখুন, code A থেকে I এর যেকোন একটি বা একাধিক বৃত্তায়িত থাকলে কোডগুলো এখানে লিখুন: _____ , _____, _____. এবার কোড/কোডগুলো দেখে দেখে মহিলাকে প্রশ্ন করুন ঃ]<br><br>আপনি বললেন যে, আপনি ----, -----, ---- এর কাছ থেকে আপনার শিশুর জন্য সেবা বা স্বাস্থ্য পরীক্ষা করেছিলেন। এখন আমাকে বলুন যে,<br>আপনি এর/এদের ভিতর যার কাছ থেকে আপনার শিশুর জন্য প্রথম সেবা বা স্বাস্থ্য পরীক্ষা করেছিলেন, তার/তাদের কাছ থেকে ডেলিভারীর কতদিন পর প্রথম সেবা গ্রহন করেছিলেন? এবং আপনি এর/এদের কাছে ডেলিভারীর ৭ দিনের ভিতর মোট কতবার আপনার শিশুর জন্য সেবা নিয়েছিলেন বা স্বাস্থ্য পরীক্ষা করেছিলেন?<br><br>*[মহিলা যদি code A থেকে I এর ভিতর একাধিক স্বাস্থ্যকর্মীর থেকে সেবা গ্রহন করে থাকেন তাহলে তাদের সবার কথাই বলুন এবং তাদের ভিতর থেকে যার কাছে সর্বপ্রথম শিশুর জন্য সেবা গ্রহন করেছিলেন তার কাছে ডেলিভারীর কতদিন পর প্রথম সেবা গ্রহন করেছিলেন সেটি এখানে লিখুন। এবং তাদের সবার কাছ থেকে ডেলিভারীর পর ১ম ৭ দিনে মোট কতবার শিশুটির জন্য সেবা গ্রহন করেছিলেন তা লিখুন।*<br><br>How many days after delivery did you visit a health worker for the first Postnatal Care for your child? In the first week after the birth of the baby how many times in total the health worker(s) came to visit you (and/or the newborn) at your home?	দিন পর Days after .... |___|___| <br>জানি না/মনে নাই DK/can't remember .... 99<br><br>বার No. Of times .... |___| একবারও আসেনি Didn't came in the 1st week .... 96<br>জানি না/ মনে নাই Don't know/don't remember .... 99<br><br>প্রযোজ্য নয় Not applicable 98	
H30	ডেলিভারীর পর আপনি যাদের কাছে আপনার শিশুর সেবা বা স্বাস্থ্য পরীক্ষা করিয়েছিলেন, তাদের কাছ থেকে আপনি আপনার শিশুর স্বাস্থ্য এবং তার যত্নের ব্যাপারে কি কি উপদেশ/পরামর্শ বা সেবা পেয়েছিলেন?<br><br>*[মহিলাকে জিজ্ঞেস করুন]* আরও কিছু? *[মহিলার নিজে থেকে দেয়া সবগুলো উত্তরই বৃত্তায়িত করুন । উত্তরগুলো পড়ে শুনাবেন না। একাধিক উত্তর হতে পারে।]*<br><br>What sort of advices have you received from the person regarding your child's care after the delivery?	বাচ্চার স্বাস্থ্য পরীক্ষা করানোর কথা / পরামর্শ দিয়েছিলেন<br>Asked for health check up for the newborn .... A<br>বুকের দুধ খাওয়ানো দেখেছে Watch me breastfeed .... B<br>সঠিক উপায়ে বুকের দুধ খাওয়ানোর কৌশল দেখিয়েছে এবং পরামর্শ দিয়েছে<br>Demonstrated proper positioning and attachment for breastfeeding .... C<br>বাচ্চার ওজন নিয়েছে Took weight of the newborn .... D<br>বাচ্চার বিপদের লক্ষণসমূহ সম্পর্কে পরামর্শ দিয়েছে<br>Counseled on danger signs for newborn .... E<br>বাচ্চাকে পরীক্ষা করেছে Examined the newborn .... F<br>অসুস্থতার জন্য বাচ্চাকে স্বাস্থ্য কেন্দ্রে রেফার করেছে<br>Referred newborn to the health center for illness .... G<br>অন্যান্য Others ____________________ X<br>নির্দিষ্ট করুন<br>কোন উপদেশ পাই নি No advice received .... Y<br>জানি না/মনে নাই Don't know/cant remember .... Z	
H31	(নাম)-এর জন্মের পর প্রথম এক মাসে (নাম)-এর কি কোন সমস্যা/জটিলতা/অসুখ হয়েছিল?<br><br>*[সবগুলো উত্তরই পড়ে শুনান।]*<br><br>After the birth of (Name) did s/he suffer from any sort problem/complication within first month of life? You have to ask all the questions.	Y N DK<br>কষ্টকর/দ্রুত শ্বাস নেয়া Difficult or fast breathing .... 1....2...9<br>বুকের খাচা ডেবে যাওয়া Chest in drawing .... 1....2...9<br>বাচ্চার শরীর ঠান্ডা হওয়া Baby feels cold .... 1....2...9<br>জ্বর/বাচ্চার শরীর গরম হওয়া Baby feels hot/Fever .... 1....2...9<br>বুকের দুধ চুষতে না পারা/খেতে না পারা Poor sucking or feeding .... 1....2...9<br>খিঁচুনি বা শরীর শক্ত হওয়া Convulsions/spasms/rigidity .... 1....2...9<br>অচেতন/অজ্ঞান/হুঁশ না থাকা Lethargy/unconsciousness .... 1....2...9	

No	Questions and filters	Options and coding category	Skip
H32	*উপরের সমস্যাগুলোর বাইরে (নাম)-এর কি অন্য কোন সমস্যা/জটিলতা/অসুখ ছিল?*<br><br>*[মহিলাকে জিজ্ঞেস করুন] আরও কিছু? [মহিলার নিজে থেকে দেয়া সবগুলো উত্তরই বৃত্তায়িত করুন । উত্তরগুলো পড়ে শুনাবেন না। একাধিক উত্তর হতে পারে।]*<br><br>After the birth of (Name) did s/he suffer from any sort problem/complication within first month of life other than those mentioned in H30? [Don't prompt]	বাচ্চা না কাঁদা Baby doesn't cry .... A<br>নিউমোনিয়া Pneumonia .... B<br>ঠান্ডা/কাশি Cold/cough .... C<br>চামড়ার রং, হাত, হাতের তালু, পায়ের পাতা, চোখ হলুদ হওয়া/জন্ডিস Yellow skin/palm/feet/eye color (jaundice) .... D<br>নাভির চারপাশে লাল হওয়া/কিছু বের হওয়া Pus, bleeding, or discharge from around umbilical cord .... E<br>চামড়ায় ফোসকা / ঘা হওয়া Skin lesions or blisters .... F<br>চোখ লাল হওয়া/ময়লা বের হওয়া Red or swollen eyes with pus .... G<br>পেসাব না হওয়া Doesn't pass urine .... H<br>পায়খানা না হওয়া Doesn't pass stool .... I<br>একটানা বমি হওয়া Continuous vomiting .... J<br>পেট ফাঁপা বা ফোলা Distention of abdomen .... K<br>বাচ্চা খুব ছোট (too small baby) .... L<br>চামড়ায় র‍্যাশ/ফুসকুড়ি/মাসিপিসি Skin rash/ 'Mashipishi' .... M<br>অন্যান্য Others ______ ... X<br>(নির্দিষ্ট করুন)<br>অসুস্থ্য ছিল না No illness/None mentioned .... Y	
H33	*[প্রশ্ন H31 এবং H32 দেখুন এবং সঠিক কোড বৃত্তায়িত করুন।]*<br>Check Question H30 & H31 and circle appropriate code	H31 তে এক বা একাধিক কোড 1 বৃত্তায়িত এবং H32 এ Y কোড ছাড়া যে কোন একটি কোড বৃত্তায়িত One or more codes circled 1 .... 1<br>H31 তে সবগুলো কোড 2/9 বৃত্তায়িত এবং H32 এ Y কোড বৃত্তায়িত All codes circled 2 2	→H42
H34	আপনার মতে (নাম)-এর সমস্যা/ জটিলতা/অসুখটি কি খুব মারাত্নক নাকি মোটমুটি নাকি সামান্য সমস্যা ছিল?<br>From your opinion, was this problem/complication severe or mild to moderate?	সামান্য ছিল Mild .... 1<br>মোটামুটি ছিল Moderate .... 2<br>মারাত্নক ছিল Severe .... 3<br>অন্যান্য Other : ______ ... 7<br>জানেন না Doesn't know 9	
H35	(নাম)-এর এই সমস্যা/জটিলতা/অসুখের জন্য আপনি কি কাউকে দেখিয়েছিলেন বা কারও সাহায্য নিয়েছিলেন?<br>Did you seek any sort of treatment for this problem/complication?	হ্যাঁ Yes .... 1<br>না No .... 2<br>জানি না/ মনে নাই Don't know/Can't remember 9	 →H41<br>→H41
H36	(নাম)-এর এই সমস্যা/জটিলতার/অসুখের জন্য আপনি কাকে দেখিয়েছিলেন বা কার সাহায্য নিয়েছিলেন?<br><br>*[মহিলাকে জিজ্ঞেস করুন] আরও কেউ? [মহিলার নিজে থেকে দেয়া সবগুলো উত্তরই বৃত্তায়িত করুন । উত্তরগুলো পড়ে শুনাবেন না। একাধিক উত্তর হতে পারে।]*<br><br>*[যদি মহিলার উত্তর সি,এস,বি,এ (Code E) বা MNCS প্রমোটর (Code H) হয়, তাহলে তাদের নাম লিখুন।]*<br><br>**Code :E** নাম Name: ______<br><br>**Code :H** নাম Name: ______<br><br>From whom did you seek treatment for this problem/complication? Do not read out the answers. Ask: Anything else? Circle all the answers	**দক্ষ/প্রশিক্ষণ প্রাপ্ত স্বাস্থ্য কর্মী (Medically trained)**<br>পাশ করা ডাক্তার (MBBS doctor) .... A<br>নার্স/ধাত্রী (Nurse/midwife) .... B<br>প্যারামেডিক/মেডিকেল এসিসটেন্ট/সাকমো (Paramedic/MA/SACMO) .... C<br>পরিবার কল্যাণ পরিদর্শক (FWV) .... D<br>সি,এস,বি,এ (CSBA) .... E<br>**অন্যান্য স্বাস্থ্য কর্মী (Other health worker)**<br>স্বাস্থ্য সহকারী/ পরিবার কল্যান সহকারী (HA /FWA) .... F<br>পুষ্টি কর্মী (CNP) .... G<br>MNCS প্রমোটর (MNCS Promoter) .... H<br>অন্যান্য কমিউনিটি স্বাস্থ্য কর্মী - এনজিও কর্মী, স্বেচ্ছাসেবী (Other CHWs, NGO worker, volunteer) .... I<br>**অন্যান্য (Other)**<br>প্রশিক্ষণ প্রাপ্ত টিবিএ (প্রশিক্ষণ প্রাপ্ত ধন্নী, চাউনী, দাই) (TTBA) .... J<br>প্রশিক্ষণহীন টিবিএ (ধন্নী, চাউনী, দাই) TBA(Dai/Dhorni/Chauni) .... K<br>হোমিওপ্যাথ/হোমিওপ্যাথ ঔষধের দোকান (Homeopath/Homeopath drug store) .... L<br>আয়ুর্বেদিক চিকিৎসক / আয়ুর্বেদিক ঔষধের দোকান /হেকিম/কবিরাজ (Ayurved/ Ayurvedic drug store /Hekim/Kabiraj) .... M<br>গ্রাম ডাক্তার (Village doctor) .... N<br>এলোপ্যাথী ঔষধের দোকান (Allopath drug store) .... O<br>ইমাম/ঝাড় ফুক/ওঝা (Spiritual healer) .... P<br>পরিবারের অন্যান্য সদস্য/আত্নীয়/ প্রতিবেশী/বন্ধু Family/relative/Neighbor/friend .... Q<br>অন্যান্য Others ______ .. X<br>(নির্দিষ্ট করুন)<br>জানি না/মনে নাই Don't know/can't remember .... Z	

No	Questions and filters	Options and coding category	Skip
H37	(নাম)-এর এই সমস্যা/জটিলতার/অসুখের জন্য আপনি কোথায় দেখিয়েছিলেন বা চিকিৎসা কোথায় পেয়েছিলেন?<br><br>*[মহিলাকে জিজ্ঞেস করুন]* আরও কোথাও? *[মহিলার নিজে থেকে দেয়া সবগুলো উত্তরই বৃত্তায়িত করুন । উত্তরগুলো পড়ে শুনাবেন না। একাধিক উত্তর হতে পারে।]*<br><br>*[মহিলা যেখান থেকে সেবা পেয়েছেন, সেই স্বাস্থ্যকেন্দ্রের নাম লিখুন। যদি একাধিক জায়গা থেকে সেবা নিয়ে থাকেন, তাহলে সবগুলো জায়গারই নাম এবং কোড লিখুন।]*<br><br>Code :|____| নাম Name: ________________________<br><br>Code :|____| নাম Name: ________________________<br><br>Code :|____| নাম Name: ________________________<br><br>Where did you go to seek care for this problem/complication? Do not read out the answers. Ask: Anything else? Circle all the answers	**বাড়ী (Home)**<br>নিজ বাড়ী, স্বামী/শ্বশুড় বাড়ী (Own home, husband/father in laws house).........A<br>বাবার বাড়ী (My natal home) ....B<br>অন্য কোন বাড়ী (Others)____________________________C<br>(নির্দিষ্ট করুন)<br>**সরকারী স্বাস্থ্য কেন্দ্র (Govt Health center)**<br>মেডিকেল কলেজ হাসপাতাল (Medical College Hospital) ....D<br>জেলা/সদর হাসপাতাল (District /Sadar Hospital) ....E<br>মা ও শিশু স্বাস্থ্য কেন্দ্র (MCWC)..F<br>উপজেলা স্বাস্থ্য কমপ্লেক্স (UHC) ....G<br>ইউনিয়ন স্বাস্থ্য ও পরিবার কল্যাণ কেন্দ্র / সাব সেন্টার/আরডি (FWC/SC/RD) H<br>কমিউনিটি ক্লিনিক (Community clinic)..I<br>সেটেলাইট ক্লিনিক/ ইপিআই কেন্দ্র (Satellite clinic/EPI centre)...................J<br>অন্যান্য সরকারী স্বাস্থ্য কেন্দ্র (Other Govt Health facility) ....K<br>**বেসরকারী স্বাস্থ্য কেন্দ্র (Non Govt Health center)**<br>এনজিও হাসপাতাল (NGO hospital)..L<br>এনজিও স্থায়ী স্বাস্থ্য কেন্দ্র (NGO static health centre) ....M<br>এনজিও সেটেলাইট ক্লিনিক (NGO satellite clinic).................................N<br>পুষ্টি কেন্দ্র (NNP centre)...O<br>অন্যান্য বেসরকারী স্বাস্থ্য কেন্দ্র (Other NGO Health facility)......................P<br>**প্রাইভেট (Private Health sector)**<br>হাসপাতাল/ ক্লিনিক (Hospital/clinic)..Q<br>স্বাস্থ্য কেন্দ্র /ডিসপেনসারী (Health centre/Dispensary) ....R<br>এমবিবিএস ডাক্তারের চেম্বার (MBBS doctor's chamber) ....S<br>গ্রাম ডাক্তারের চেম্বার (Village doctor's chamber) ....T<br>প্যারামেডিক/মেডিকেল এসিসটেন্ট/সাকমোর চেম্বার (Paramedic/MA/SACMO chamber) U<br>এলোপ্যাথী ঔষধের দোকান (Allopath drug store) ....V<br>অন্যান্য প্রাইভেট_স্বাস্থ্য কেন্দ্র (Other private Health facility.......................W<br>অন্যান্য (Others) ____________________________..X<br>(নির্দিষ্ট করুন)	
H38	(নাম)-এর এই সমস্যা/জটিলতার জন্য কাউকে দেখানো বা চিকিৎসার ব্যাপারে কেউ কি আপনাকে পরামর্শ দিয়েছিল ?<br>Did anyone advise you or refer you to seek care for your complication?	হ্যাঁ Yes...1<br>না No ....2<br>জানি না/ মনে নাই Don't know/Can't remember 9	 →H42<br>→H42

No	Questions and filters	Options and coding category	Skip
H39	(নাম)-এর এই সমস্যা/জটিলতার/অসুখের জন্য কাউকে দেখানো বা চিকিৎসার ব্যাপারে কে আপনাকে পরামর্শ দিয়েছিল?<br><br>*[মহিলাকে জিজ্ঞেস করুন]* আরও কেউ ? *[মহিলার নিজে থেকে দেয়া সবগুলো উত্তরই বৃত্তায়িত করুন । উত্তরগুলো পড়ে শুনাবেন না । একাধিক উত্তর হতে পারে ।]*<br><br>*[যদি মহিলার উত্তর সি,এস,বি,এ (Code E) বা MNCS প্রমোটর (Code H) হয়, তাহলে তাদের নাম লিখুন ।]*<br><br>**Code :E** নাম Name: ______________________________<br><br>**Code :H** নাম Name: ______________________________<br><br>Who had advised you or refered you to seek care for your complication? Do not read out the answers. Ask: Anything else? Circle all the answers	**দক্ষ/প্রশিক্ষণ প্রাপ্ত স্বাস্থ্য কর্মী (Medically trained)**<br>পাশ করা ডাক্তার (MBBS doctor) .... A<br>নার্স/ধাত্রী (Nurse/midwife)........ B<br>প্যারামেডিক/মেডিকেল এসিসটেন্ট/সাকমো (Paramedic/MA/SACMO) .... C<br>পরিবার কল্যাণ পরিদর্শক (FWV)........ D<br>সি,এস,বি,এ (CSBA) .... E<br>**অন্যান্য স্বাস্থ্য কর্মী (Other health worker)**<br>স্বাস্থ্য সহকারী/ পরিবার কল্যান সহকারী (HA /FWA) .... F<br>পুষ্টি কর্মী (CNP) .... G<br>MNCS প্রমোটর (MNCS Promoter)........ H<br>অন্যান্য কমিউনিটি স্বাস্থ্য কর্মী - এনজিও কর্মী, স্বেচ্ছাসেবী (Other CHWs, NGO worker, volunteer)........ I<br>**অন্যান্য (Other)**<br>প্রশিক্ষণ প্রাপ্ত টিবিএ (প্রশিক্ষণ প্রাপ্ত ধন্নী, চাউনী, দাই) (TTBA) .... J<br>প্রশিক্ষণহীন টিবিএ (ধন্নী, চাউনী, দাই) TBA(Dai/Dhorni/Chauni)........ K<br>হোমিওপ্যাথ/হোমিওপ্যাথ ঔষধের দোকান (Homeopath/Homeopath drug store) .... L<br>আয়ুর্বেদিক চিকিৎসক / আয়ুর্বেদিক ঔষধের দোকান /হেকিম/কবিরাজ (Ayurved/ Ayurvedic drug store /Hekim/Kabiraj) .... M<br>গ্রাম ডাক্তার (Village doctor)........ N<br>এলোপ্যাথী ঔষধের দোকান (Allopath drug store) .... O<br>ইমাম/ঝাড় ফুক/ওঝা (Spiritual healer)........ P<br>পরিবারের অন্যান্য সদস্য/আত্মীয়/ প্রতিবেশী/বন্ধু Family/relative/Neighbor/friend........ Q<br>অন্যান্য Others ______________________ .. X<br>(নির্দিষ্ট করুন)<br>জানি না/মনে নাই Don't know/can't remember........ Z	
H40	(নাম)-এর এই সমস্যা/জটিলতার জন্য কাউকে দেখানো বা চিকিৎসার ব্যাপারে যিনি আপনাকে পরামর্শ করেছিলেন, তিনি কি আপনাকে কোন কাগজ (রেফারেল স্লিপ) দিয়েছিলেন?<br>*[রেফারেল স্লিপের একটি নমুনা দেখান]*<br>Did the person who had referred you issue you a referral slip to show it to the health facility/carer ? (show the referral slip to the respondent)	হ্যাঁ Yes........ 1<br>না No .... 2<br>জানি না/ মনে নাই Don't know/Can't remember 9	→H42<br>→H42<br>→H42
H41	আপনি কেন (নাম)-এর এই সমস্যা/জটিলতার/অসুখের জন্য কাউকে দেখান নি বা চিকিৎসা নেন নি?<br><br>*[মহিলাকে জিজ্ঞেস করুন]* আরও কিছু? *[মহিলার নিজে থেকে দেয়া সবগুলো উত্তরই বৃত্তায়িত করুন । উত্তরগুলো পড়ে শুনাবেন না । একাধিক উত্তর হতে পারে ।]*<br><br>Why did you not seek care for this complication? Do not read out the answers. Ask: Anything else? Circle all the answers	সেবার প্রয়োজন আছে বলে মনে হয় নি/সেবার প্রয়োজন নেই Didn't think it was necessary to seek care .... A<br>জানতাম না কোথায় যেতে হবে Not known where to go .... B<br>অনেক খরচ/ টাকা পয়সা ছিল না Too costly/ Lack of money........ C<br>স্বাস্থ্য কেন্দ্র বাসা হতে অনেক দুরে Too far from house........ D<br>যানবাহনের সমস্যা Transport problem .... E<br>সাথে যাবার মত কেউ ছিল না No one accompanied .... F<br>স্বাস্থ্যকেন্দ্রে যাবার মত সময় ছিল না Not enough time to go .... G<br>পরিবার আমাকে যেতে দেন নি Family didn't allow me to go........ H<br>ধর্মে মানা/বাধা Religeous bar .... I<br>স্বাস্থ্যকেন্দ্রের সেবাদানের সময় সীমা সুবিধাজনক নয় Service hr inconvenient ...J<br>স্বাস্থ্যকেন্দ্র বন্ধ ছিল/কোন স্বাস্থ্যকর্মী ছিলেন না HF found closed/nobody there..... K<br>স্বাস্থ্যকেন্দ্রে সেবা অনুন্নত মানের Poor quality of services at facility .... L<br>স্বাস্থ্যকেন্দ্রে নিম্নমানের এবং অদক্ষ সেবাপ্রদানকারী Poor quality & staffs at HF ..M<br>স্বাস্থ্যকেন্দ্রে পর্দার অভাব Lack of privacy .... N<br>স্বাস্থ্যকেন্দ্রের সেবাদানকারীদের ব্যবহার খারাপ Unpleasant behavior at center ...O<br>স্বাস্থ্যকেন্দ্রের অনেকক্ষন বসে থাকতে হয় সেবা পাবার জন্য Long queue at HF .P<br>স্বাস্থ্যকেন্দ্রের ঔষধ পত্র পাওয়া যায়না Inadequate drugs at the health center .... Q<br>অন্যান্য Others ______________________ .. X<br>(নির্দিষ্ট করুন)	

No	Questions and filters	Options and coding category	Skip
H42	আপনি কি বলতে পারবেন, জন্মের পর একটি বাচ্চার কি কি সমস্যা/জটিলতা/অসুখ হতে পারে ? আমাকে কি বলবেন?<br><br>*[মহিলাকে জিজ্ঞেস করুন]* আরও কিছু? *[মহিলার নিজে থেকে দেয়া সবগুলো উত্তরই বৃত্তায়িত করুন । উত্তরগুলো পড়ে শুনাবেন না। একাধিক উত্তর হতে পারে।]*<br><br>After the birth of a child what sort of problems/complications may occur within first month of life? Do not read out, Ask anything else? Record all the answers she mentions.	কষ্টকর/দ্রুত শ্বাস নেয়া Difficult or fast breathing .... A<br>নিউমোনিয়া Pneumonia .... B<br>ঠান্ডা/কাশি Cold/cough .... C<br>বুকের খাচা ডেবে যাওয়া Chest in drawing .... D<br>বুকের দুধ চুষতে না পারা Poor sucking or feeding .... E<br>চামড়ার রং, হাত, হাতের তালু, পায়ের পাতা, চোখ হলুদ হওয়া/জন্ডিস Yellow skin/palm/feet/eye color (jaundice) .... F<br>নাভির চারপাশে লাল হওয়া/কিছু বের হওয়া Pus, bleeding, or discharge from around the umbilical cord .... G<br>চামড়ায় ফোসকা / ঘা হওয়া Skin lesions or blisters .... H<br>খিঁচুনি বা শরীর শক্ত হওয়া Convulsions/spasms/rigidity .... I<br>অচেতন/অজ্ঞান/হুঁশ না থাকা Lethargy/unconsciousness .... J<br>চোখ লাল হওয়া/ময়লা বের হওয়া Red or swollen eyes with pus .... K<br>বাচ্চার শরীর ঠান্ডা হওয়া Baby feels cold .... L<br>জ্বর/বাচ্চার শরীর গরম হওয়া Baby feels hot/Fever .... M<br>বাচ্চা না কাঁদা Baby doesn't cry .... N<br>পেসাব না হওয়া Doesn't pass urine .... O<br>পায়খানা না হওয়া Doesn't pass stool .... P<br>একটানা বমি হওয়া Continuous vomiting .... Q<br>পেট ফাঁপা বা ফোলা Distention of abdomen .... R<br>বাচ্চা খুব ছোট হওয়া Too small baby .... S<br>চামড়ায় র‍্যাশ/ফুসকুড়ি/মাসিপিসি Skin rash/ 'Mashipishi' .... T<br>অন্যান্য Others ________________ ... X<br>(Specify)<br>অসুস্থ্য ছিল না No illness/None mentioned .... Y<br>জানা নাই (Don't know) .... Z	
H43	জন্মের পর একটি বাচ্চার সমস্যা/জটিলতার/অসুখের জন্য কোথায় যাওয়া যেতে পারে বা চিকিৎসা নেয়া যায়, তা কি আমাকে বলবেন?<br><br>*[মহিলাকে জিজ্ঞেস করুন]* আরও কিছু? *[মহিলার নিজে থেকে দেয়া সবগুলো উত্তরই বৃত্তায়িত করুন । উত্তরগুলো পড়ে শুনাবেন না। একাধিক উত্তর হতে পারে।]*<br><br>Can you tell me from where you can sought care for medical check-up for complication during pregnancy, deliveryand after delivery?	**<u>সরকারী স্বাস্থ্য কেন্দ্র (Govt Health center)</u>**<br>মেডিকেল কলেজ হাসপাতাল (Medical College Hospital) .... A<br>জেলা/সদর হাসপাতাল (District /Sadar Hospital) .... B<br>মা ও শিশু স্বাস্থ্য কেন্দ্র (MCWC) .... C<br>উপজেলা স্বাস্থ্য কমপ্লেক্স (UHC) .... D<br>ইউনিয়ন স্বাস্থ্য ও পরিবার কল্যাণ কেন্দ্র/সাব সেন্টার/আরডি (FWC/SC/RD) ... E<br>কমিউনিটি ক্লিনিক (Community clinic) .... F<br>সেটেলাইট ক্লিনিক/ ইপিআই কেন্দ্র (Satellite clinic/EPI centre) .... G<br>অন্যান্য সরকারী স্বাস্থ্য কেন্দ্র (Other Govt Health facility) .... H<br>**<u>বেসরকারী স্বাস্থ্য কেন্দ্র (Non Govt Health center)</u>**<br>এনজিও হাসপাতাল (NGO hospital) .... I<br>এনজিও স্থায়ী স্বাস্থ্য কেন্দ্র (NGO static health centre) .... J<br>এনজিও সেটেলাইট ক্লিনিক (NGO satellite clinic) .... K<br>পুষ্টি কেন্দ্র (NNP centre) .... L<br>অন্যান্য বেসরকারী স্বাস্থ্য কেন্দ্র (Other NGO Health facility) .... M<br>**<u>প্রাইভেট (Private Health sector)</u>**<br>হাসপাতাল/ ক্লিনিক (Hospital/clinic) .... N<br>স্বাস্থ্য কেন্দ্র /ডিসপেনসারী (Health centre/Dispensary) .... O<br>এমবিবিএস ডাক্তারের চেম্বার (MBBS doctor's chamber) .... P<br>গ্রাম ডাক্তারের চেম্বার (Village doctor's chamber) .... Q<br>প্যারামেডিক/মেডিকেল এসিসটেন্ট/সাকমোর চেম্বার (Paramedic/ MA/SACMO chamber) R<br>এলোপ্যাথী ঔষধের দোকান (Allopath drug store) .... S<br>অন্যান্য প্রাইভেট_স্বাস্থ্য কেন্দ্র (Other private Health facility) .... T<br>অন্যান্য (Others) ________________ X<br>(নির্দিষ্ট করুন)<br>কোথায় যেতে হবে, জানা নাই (Don't know) .... Z	

No	Questions and filters	Options and coding category	Skip
H44	*[প্রশ্ন নং H42 এবং H43 দেখুন। যদি মা জন্মের পর বাচ্চার একটিও সমস্যা/জটিলতার/অসুখের কথা বা/এবং তার জন্য কোথায় চিকিৎসা নিতে যাওয়া যায় তার কথা বলতে পারেন, তাহলে মহিলাকে জিজ্ঞেস করুন;]*<br><br>আপনি বললেন যে, জন্মের পর বাচ্চার -----, ---- সমস্যা/জটিলতা/অসুখ হলেও হতে পারে। এবং সেবা বা চিকিৎসার জন্য -----, ----- জায়গায় যাওয়া যেতে পারে - আপনি এই তথ্য কোথা থেকে বা কার কাছ থেকে পেয়েছেন?<br><br>*[মহিলাকে জিজ্ঞেস করুন]* আরও কিছু? *[মহিলার নিজে থেকে দেয়া সবগুলো উত্তরই বৃত্তায়িত করুন । উত্তরগুলো পড়ে শুনাবেন না। একাধিক উত্তর হতে পারে।]*<br><br>If mother mentions one or more of the above listed danger signs, Ask. from where /who provided you with the	ডাক্তার/নার্স/ধাত্রী/প্যারামেডিক (Doctor/ Nurse/Midwife/ Paramedics) .... A<br>স্বাস্থ্য কেন্দ্র/হাসপাতাল (Health facility/ Clinic/ Hospital)............................ B<br>কমিউনিটি স্বাস্থ্য কর্মী - স্বাস্থ্য সহকারী/পরিবার কল্যান সহকারী, পুষ্টি কর্মী, এনজিও কর্মী, MNCS প্রমোটর, স্বেচ্ছাসেবী (CHWs -HAS, FWAs, CNPs, MNCS promoter,NGO worker, volunteer)... C<br>কমিউনিটি গ্রুপ মিটিং / মিটিং /সভা থেকে (Community meeting) .... D<br>রেডিও/টিভি (Radio /TV) .... E<br>সংবাদপত্র/খবরের কাগজ/বইপত্র (Newspaper/Books) .... F<br>পোষ্টার / কোন ছাপানো কাগজ (Poster/Leaflet)...................................... G<br>পরিবারের অন্যান্য সদস্য/আত্মীয়/প্রতিবেশী/বন্ধু Family/relatives/Neighbor/friend ..... H<br>অন্যান্য Others_______________________________________... X<br>(নির্দিষ্ট করুন)<br>মনে নাই Can't remember .... Z	

Module IV

Feeding Practice

*[Question D15 থেকে ১লা জুন 2007 হতে এ পর্যন্ত যেসব সন্তানের জন্ম হয়েছে তাদের প্রত্যেকের লাইন নম্বর, নাম এবং জীবিত না মৃত তা নীচের টেবিলে লিখুন । সর্বশেষ সন্তান দিয়ে প্রশ্ন শুরু করুন । **(তিন এর অধিক সন্তান থাকলে অতিরিক্ত ফরম-এর শেষ দুই কলাম ব্যবহার করুন)**।]*

No	Questions and filters	সর্বশেষ বাচ্চা<br>*Last Birth*	সর্বশেষ থেকে শুরু করে ২য় বাচ্চা<br>*Next to last birth*	সর্বশেষ থেকে শুরু করে ৩য় বাচ্চা<br>*2nd from last birth*	Skip
I00a	লাইন নং Line number from D13	|____|____|	|____|____|	|____|____|	
I00b	নাম Name from D18	___________	___________	___________	
I00c	শিশুটি কি এখন জীবিত/মৃত Child survival status from D20	Dead………. 1 →<br>Living ………2 ↓	Dead………. 1 →<br>Living ………2 ↓	Dead………. 1 →<br>Living ………2 ↓	STOP

[১লা জুন 2007 হতে এ পর্যন্ত যেসব সন্তানের জন্ম হয়েছে এবং তাদের মধ্যে যারা জীবিত তাদের সকলের সম্পর্কে I01 হতে প্রশ্ন গুলো করুন । সর্বশেষ সন্তান দিয়ে প্রশ্ন করুন । (তিন এর অধিক সন্তান থাকলে অতিরিক্ত ফরম-এর শেষ দুই কলাম ব্যবহার করুন)] *[Ask the question from I01 for all currently live children who born in June 2007 or later. Start with last child. (If there is more than 3 births, use last 2 columns of additional questionnaire)]*

Section I: Feeding Practice

No	Questions and filters	Options and coding category	সর্বশেষ বাচ্চা<br>Last Birth	সর্বশেষ থেকে শুরু করে ২য় বাচ্চা<br>Next to last birth	সর্বশেষ থেকে শুরু করে ৩য় বাচ্চা<br>2nd to last birth	Skip
I01	আপনি কি আমাকে বলতে পারেন কত মাস বয়স পর্যন্ত শিশুকে শুধু মাত্র বুকের দুধ খেতে দিতে হয় ? How long (till what age of the baby) exclusive breastfeeding should be continued?			মাস months ……… ……. |___|___| মাস<br>(জানিনা হলে 99 লিখুন)		
I02	<নাম> কে কখনও বুকের দুধ খাইয়েছিলেন কি ?<br>*[সর্বশেষ বাচ্চার ক্ষেত্রে G39 এ দেয়া উত্তরের সাথে মিলিয়ে দেখুন]*<br>Have you ever breastfed NAME?<br>*[Check with the answer given in G39]*	হ্যাঁ Yes.. 1<br>না No 2		.... 1<br>....................... 2	.... 1<br>....................... 2	 →I06
I03	<নাম> কি এখনও বুকের দুধ খায়?<br>Are you still breast-feeding?	হ্যাঁ Yes.. 1<br>না No 2		.... 1<br>....................... 2	.... 1<br>....................... 2	→I05
I04	<নাম> -এর জন্মের পর কত মাস পর্যন্ত তাকে বুকের দুধ খাইয়েছিলেন ?<br>*[1 (এক)মাসের কম হলে মাসের ঘরে 00 লিখুন]*<br>How many months after birth have you breastfeed your child?	মাস month	|_____|_____|	|_____|_____|	|_____|_____|	→I06
I05	গতকাল সারাদিনে এবং রাতে (সকাল থেকে রাত) <নাম> কে কি একবারও বুকের দুধ খাইয়েছিলেন?<br>Was (NAME) breastfed yesterday during the day or at night?	হ্যাঁ Yes.. 1<br>না No 2		.... 1<br>....................... 2	.... 1<br>..................... 2	
I06	মাঝে মাঝে মা তার শিশুকে বুকের দুধ সনাতনী উপায়ে না খাইয়ে অন্য উপায়েও খাইয়ে থাকেন। যেমন: বুকের দুধ অন্য উপায়ে বের করে তা চামচ, কাপ বা বোতলে করে পান করান। এটা সাধারন তখনই হয় যখন মা দীর্ঘ সময় তার শিশুকে যদি সময় দিতে না পারেন। আবার মাঝে মাঝে এমন ঘটনাও হয় যে, মা শিশুকে বুকের দুধ খাওয়ানোর মত পর্যাপ্ত পরিমান দুধ না পেয়ে থাকেন। সেক্ষেত্রে দেখা যায় যে, সেই শিশুকে নিজের মায়ের বুকের দুধ না দিয়ে অন্য কোন মহিলার বুকের দুধ বের করে কাপে, বোতলে বা চামচে দেয়া হচ্ছে। Sometimes babies are fed breast milk in different ways, for example by spoon, cup or bottle. This can happen when the mother cannot always be with her baby. Sometimes babies are breastfed by another woman, or given breast milk from another woman by spoon, cup or bottle or some other way. This can happen if a mother cannot breastfeed her own baby.					
	আপনার শিশুটিকে কখনও এমন কোন উপায়ে বুকের দুধ খাওয়ানো হয়েছে কি?<br>Has (NAME) ever consumed breastmilk in any of these ways?	হ্যাঁ Yes.. 1<br>না No .... 2<br>জানি না/মনে নাই Don't know/CR.................. 9		.... 1<br>....................... 2<br>....................... 9	.... 1<br>....................... 2<br>....................... 9	 →I08<br>→I08

No	Questions and filters	Options and coding category	সর্বশেষ বাচ্চা Last Birth	সর্বশেষ থেকে শুরু করে ২য় বাচ্চা Next to last birth	সর্বশেষ থেকে শুরু করে ৩য় বাচ্চা 2nd to last birth	Skip
I07	গতকাল সারাদিন (সকাল ও রাত) আপনার শিশুকে এমন কোন উপায়ে বুকের দুধ খাইয়েছেন কি? Did (NAME) consume breast-milk in any of these ways yesterday during the day or at night?	হ্যাঁ Yes<br>না No<br>জানি না/মনে নাই Don't know/CR	1<br>2<br>9	1<br>2<br>9	1<br>2<br>9	
I08	শিশুকে গত 24 ঘন্টার মধ্যে বুকের দুধ ছাড়া অন্য কোন খাবার বা পানীয় খাওয়ানো হয়েছে কি? Do you give (Name) anything else to drink or eat beside breast milk in the last 24 hours?	হ্যাঁ Yes<br>না No	1<br>2	1<br>2	1<br>2	→J00a
I09	শিশুকে গত 24 ঘন্টার মধ্যে কোন সময়ে কি এ খাবার গুলো খেতে দিয়েছিলেন? যেমনঃ *(প্রত্যেকটি খাবার সম্পর্কে পড়ে শোনান)* Now I will ask you about the type of solid, liquid or semi-solid food you have given to (NAME) in last 24 hours. Among the following liquid, solid and semi-solid food what have you given to (NAME)? *[Ask for every item]*		Y ... N ... DK	Y ... N ... DK	Y ... N ... DK	
		গরু বা ছাগলের দুধ? Cow's or goat milk	1 ... 2 ... 9	1 ... 2 ... 9	1 ... 2 ... 9	
		অন্যান্য তরল খাবার? Other liquid food	1 ... 2 ... 9	1 ... 2 ... 9	1 ... 2 ... 9	
		শিশু খাদ্য (বেবী ফর্মুলা) Baby formula/baby food	1 ... 2 ... 9	1 ... 2 ... 9	1 ... 2 ... 9	
		সুজী/লেই Shuji	1 ... 2 ... 9	1 ... 2 ... 9	1 ... 2 ... 9	
		ভাত/রুটি/খিচুড়ী? Rice, bread, khichuri	1 ... 2 ... 9	1 ... 2 ... 9	1 ... 2 ... 9	
		পানি খেতে দিয়েছিলেন Water	1 ... 2 ... 9	1 ... 2 ... 9	1 ... 2 ... 9	
		মধু, চিনির পানি বা ফলের রস? Honey, sugar water or fruit juice	1 ... 2 ... 9	1 ... 2 ... 9	1 ... 2 ... 9	
		কলা, পাকা পেঁপে বা আম? Mango, ripe papaya	1 ... 2 ... 9	1 ... 2 ... 9	1 ... 2 ... 9	
		সবুজ শাকসবজি? Green vegetable	1 ... 2 ... 9	1 ... 2 ... 9	1 ... 2 ... 9	
		অন্যান্য ফল বা সব্জী? Other fruits or vegetables	1 ... 2 ... 9	1 ... 2 ... 9	1 ... 2 ... 9	
		মাংস, মাছ বা ডিম? Fish, meat or egg	1 ... 2 ... 9	1 ... 2 ... 9	1 ... 2 ... 9	
		ডাল Pulses	1 ... 2 ... 9	1 ... 2 ... 9	1 ... 2 ... 9	
		অন্যান্য Others	1 ... 2 ... 9	1 ... 2 ... 9	1 ... 2 ... 9	
I10	গত 24 ঘন্টায় শিশুকে বুকের দুধ ছাড়া মোট কতবার শক্ত, আধা শক্ত বা তরল খাবার খেয়েছে? *(7 বার বা তার অধিকবার হলে বক্সে 7 লিখুন, উত্তর না হলে অর্থাৎ না খাওয়ালে বক্সে 0 লিখুন)* How many times did (Name) eat solid, semi-solid, liquid food other than breast milk in last 24 hours? *[IF 7 OR MORE TIMES , RECORD 7 IF NONE , RECORD 0]*	24 ঘন্টায় কতবার শক্ত, আধা শক্ত বা তরল খাবার খেয়েছে	|____| বার	|____| বার	|____| বার	

Module V

Child Section

*[Question D15 থেকে ১লা জুন 2007 হতে এ পর্যন্ত যেসব সন্তানের জন্ম হয়েছে তাদের প্রত্যেকের লাইন নম্বর, নাম এবং জীবিত না মৃত তা নীচের টেবিলে লিখুন । সর্বশেষ সন্তান দিয়ে প্রশ্ন শুরু করুন । **(তিন এর অধিক সন্তান থাকলে অতিরিক্ত ফরম-এর শেষ দুই কলাম ব্যবহার করুন)** ।]*

[Enter in the table the line number and name and survivial status of each birth in June 2007 or later from Question D15. Start with last birth. (If there is more than 3 births, use last 2 columns of additional questionnaire).]

<table>
<tr><th>No</th><th>Questions and filters</th><th>সর্বশেষ বাচ্চা
Last Birth</th><th>সর্বশেষ থেকে শুরু করে ২য় বাচ্চা
Next to last birth</th><th>সর্বশেষ থেকে শুরু করে ৩য় বাচ্চা
2nd to last birth</th><th>Skip</th></tr>
<tr><td>J00a</td><td>লাইন নং Line number from D13</td><td>|____|____|</td><td>|____|____|</td><td>|____|____|</td><td></td></tr>
<tr><td>J00b</td><td>নাম Name from D18</td><td>___________</td><td>___________</td><td>___________</td><td></td></tr>
<tr><td>J00c</td><td>বাচ্চা কি জীবিত না মৃত Child survivial status from D20</td><td>Dead………. 1 →
Living ………2 ↓</td><td>Dead………. 1 →
Living ………2 ↓</td><td>Dead………. 1 →
Living ………2 ↓</td><td>STOP</td></tr>
</table>

[১লা জুন 2007 হতে এ পর্যন্ত যেসব সন্তানের জন্ম হয়েছে এবং তাদের মধ্যে যারা জীবিত তাদের সকলের সম্পর্কে J01 হতে প্রশ্ন গুলো করুন । সর্বশেষ সন্তান দিয়ে প্রশ্ন করুন । (তিন এর অধিক সন্তান থাকলে অতিরিক্ত ফরম-এর শেষ দুই কলাম ব্যবহার করুন)]

[Ask the question from J01 for all currently live children who born in June 2007 or later. Start with last child. (If there is more than 3 births, use last 2 columns of additional questionnaire)]

Section J: Vaccination and Vitamin

This section contains some information about health care of the baby

<table>
<tr><th>No</th><th>Questions and filters</th><th>Options and coding category</th><th>সর্বশেষ বাচ্চা
Last Birth</th><th>সর্বশেষ থেকে শুরু করে ২য় বাচ্চা
Next to last birth</th><th>সর্বশেষ থেকে শুরু করে ৩য় বাচ্চা
2nd to last birth</th><th>Skip</th></tr>
<tr><td>J01</td><td>আপনার শিশুর কি টিকা কার্ড আছে? বা এমন কোন কার্ড আছে যেখানে টিকা এবং তার তারিখগুলো লেখা আছে? Is there a vaccination card for <CHILD> or any other card that have the date of child vaccination?</td><td colspan="2">হ্যাঁ Yes...1
না No 2
জানি না/মনে নাই Don’t know/CR...........9</td><td>...................... 1
.... 2
.... 9</td><td>........................1
........................2
........................9</td><td>
→J06
→J06</td></tr>
<tr><td>J02</td><td>আমি কি কার্ডটি দেখতে পারি?
May I see the card?</td><td colspan="2">কার্ড দেখিয়েছেন Showed the card 1
কার্ড দেখাতে পারেননি Didn't shown the card 2</td><td>...................... 1
.... 2</td><td>........................1
........................2</td><td>
→J06</td></tr>
<tr><td>J03</td><td colspan="5">[টিকা কার্ড হতে প্রাপ্ত সবগুলো টিকা এবং তার প্রাপ্তির তারিখগুলো এখানে লিখুন ।]
Copy all dates of vaccinations from the card to the table</td><td></td></tr>
<tr><td></td><td colspan="2">বিসিজি BCG ....</td><td>-|__|__|-|__|__|-|__|__|</td><td>-|__|__|-|__|__|-|__|__|</td><td>-|__|__|-|__|__|-|__|__|</td><td></td></tr>
<tr><td></td><td colspan="2">পোলিও ০ Polio 0 ....</td><td>-|__|__|-|__|__|-|__|__|</td><td>-|__|__|-|__|__|-|__|__|</td><td>-|__|__|-|__|__|-|__|__|</td><td></td></tr>
<tr><td></td><td colspan="2">পোলিও ১ Polio 1 ....</td><td>-|__|__|-|__|__|-|__|__|</td><td>-|__|__|-|__|__|-|__|__|</td><td>-|__|__|-|__|__|-|__|__|</td><td></td></tr>
<tr><td></td><td colspan="2">পোলিও ২ Polio 2 ....</td><td>-|__|__|-|__|__|-|__|__|</td><td>-|__|__|-|__|__|-|__|__|</td><td>-|__|__|-|__|__|-|__|__|</td><td></td></tr>
<tr><td></td><td colspan="2">পোলিও ৩ Polio 3 ....</td><td>-|__|__|-|__|__|-|__|__|</td><td>-|__|__|-|__|__|-|__|__|</td><td>-|__|__|-|__|__|-|__|__|</td><td></td></tr>
<tr><td></td><td colspan="2">ডিপিটি ১ DPT 1..</td><td>-|__|__|-|__|__|-|__|__|</td><td>-|__|__|-|__|__|-|__|__|</td><td>-|__|__|-|__|__|-|__|__|</td><td></td></tr>
<tr><td></td><td colspan="2">ডিপিটি ২ DPT 2..</td><td>-|__|__|-|__|__|-|__|__|</td><td>-|__|__|-|__|__|-|__|__|</td><td>-|__|__|-|__|__|-|__|__|</td><td></td></tr>
<tr><td></td><td colspan="2">ডিপিটি ৩ DPT 3 ....</td><td>-|__|__|-|__|__|-|__|__|</td><td>-|__|__|-|__|__|-|__|__|</td><td>-|__|__|-|__|__|-|__|__|</td><td></td></tr>
<tr><td></td><td colspan="2">হাম Measles ....</td><td>-|__|__|-|__|__|-|__|__|</td><td>-|__|__|-|__|__|-|__|__|</td><td>-|__|__|-|__|__|-|__|__|</td><td></td></tr>
<tr><td></td><td colspan="2">হেপা বি ১ HepB1 ....</td><td>-|__|__|-|__|__|-|__|__|</td><td>-|__|__|-|__|__|-|__|__|</td><td>-|__|__|-|__|__|-|__|__|</td><td></td></tr>
<tr><td></td><td colspan="2">হেপা বি ২ HepB2 ....</td><td>-|__|__|-|__|__|-|__|__|</td><td>-|__|__|-|__|__|-|__|__|</td><td>-|__|__|-|__|__|-|__|__|</td><td></td></tr>
<tr><td></td><td colspan="2">হেপা বি ৩ HepB3 ....</td><td>-|__|__|-|__|__|-|__|__|</td><td>-|__|__|-|__|__|-|__|__|</td><td>-|__|__|-|__|__|-|__|__|</td><td></td></tr>
<tr><td></td><td colspan="2">হিব ১ Hib 1..</td><td>-|__|__|-|__|__|-|__|__|</td><td>-|__|__|-|__|__|-|__|__|</td><td>-|__|__|-|__|__|-|__|__|</td><td></td></tr>
<tr><td></td><td colspan="2">হিব ২ Hib 2..</td><td>-|__|__|-|__|__|-|__|__|</td><td>-|__|__|-|__|__|-|__|__|</td><td>-|__|__|-|__|__|-|__|__|</td><td></td></tr>
<tr><td></td><td colspan="2">হিব ৩ Hib 3 ....</td><td>-|__|__|-|__|__|-|__|__|</td><td>-|__|__|-|__|__|-|__|__|</td><td>-|__|__|-|__|__|-|__|__|</td><td></td></tr>
</table>

No	Questions and filters	Options and coding category	সর্বশেষ বাচ্চা Last Birth	সর্বশেষ থেকে শুরু করে ২য় বাচ্চা Next to last birth	সর্বশেষ থেকে শুরু করে ৩য় বাচ্চা 2nd to last birth	Skip
J04	<নাম> কে এমন কোনো টিকা দেয়া হয়েছে কি, যা এ কার্ডে লেখা নাই? Has the child received any other vaccine except the doses mentioned in the card?	হ্যাঁ Yes.........1<br>না No.........2<br>জানি না/মনে নাই Don't know/CR.........9		.........1<br>.........2<br>.........9	.........1<br>.........2<br>.........9	 →J16<br>→J16
J05	টিকা কার্ডে লেখা টিকাগুলোর বাইরে আর কোন কোন টিকা পেয়েছিলেন? What are the other vaccines given outside the card?	টিকার নাম Name of vaccine________<br>বার How many times ....|___|___| জানা নাই/ মনে নাই Don't know/CR.........9		______<br>.........|___|___| 9	______<br>.........|___|___| 9	Go to J16 For all
J06	<নাম> -কে কি যক্ষা প্রতিরোধের জন্য বিসিজি টিকা দেয়া হয়েছে (বাম কাধে যে ইনজেকশন দেয়া হয় এবং যার চিহ্ন/দাগ থাকে) Did (name) receive A BCG vaccination against tuberculosis, that is, an injection in the left shoulder that caused a scar?	হ্যাঁ Yes.........1<br>না No.........2<br>জানি না/মনে নাই Don't know/CR.........9		.........1<br>.........2<br>.........9	.........1<br>.........2<br>.........9	
J07	<নাম> -কে কি পোলিও টিকা খাওয়ানো হয়েছিল (অর্থাৎ যা মুখে ফোটা ফোটা করে ঢেলে খাওয়ানো হয়) Did (NAME) receive Polio vaccine that is, drops in the mouth?	হ্যাঁ Yes.........1<br>না No.........2<br>জানি না/মনে নাই Don't know/CR.........9		.........1<br>.........2<br>.........9	.........1<br>.........2<br>.........9	 →J10<br>→J10
J08	<নাম> -কে সর্বপ্রথম কখন পোলিও টিকা দেয়া হয়েছিল? সেটা কি জন্মের পরপরই অর্থাৎ দুই সপ্তাহের মধ্যে নাকি পরবর্তী কোন সময়ে? When was the first polio vaccine received, just after birth or later?	জন্মের পরপরই বা প্রথম দুই সপ্তাহের ভিতর Within first two wks.........1<br>২ সপ্তাহের পরে Later.........2<br>জানি না Don't know.........9		.........1<br>.........2<br>.........9	.........1<br>.........2<br>.........9	
J09	কতবার শিশুটি পোলিও টিকা পেয়েছিল? (রুটিন টিকা) How many times did (NAME) receive polio vaccine	কত বার How many times ....|___|___| বার<br>*[জানিনা হলে 99 লিখুন]*		.... |___|___| বার	.... |___|___| বার	
J10	<নাম> -কে কি DPT টিকা দেয়া হয়েছিল,অর্থাৎ যে ইনজেকশন উরুর বা পাছার মাংশ পেশীতে দেয়া হয় এবং মাঝে মধ্যে পোলিও ড্রপের সাথে একই সময়ে দেয়া হয়? Did (NAME) receive DPT vaccination, that is, an injection given in the thigh or buttocks, sometimes at the same time as polio drops?	হ্যাঁ Yes.........1<br>না No.........2<br>জানি না/মনে নাই Don't know/CR.........9		.........1<br>.........2<br>.........9	.........1<br>.........2<br>.........9	 →J12<br>→J12
J11	<নাম> DPT টিকা কত বার পেয়েছিল?How many times?	কত বার How many times ...... |___| বার<br>*[জানিনা হলে 9 লিখুন]*		.... |___| বার	.... |___| বার	
J12	<নাম> -কে কি হাম প্রতিরোধের জন্য কোনো টিকা টিকা দেয়া হয়েছিল যা 9 মাস বা তার বেশি বয়সী শিশুদের হাতে দেয়া হয়? Did (NAME) receive an injection to prevent measles?	হ্যাঁ Yes.........1<br>না No.........2<br>জানি না/মনে নাই Don't know/CR.........9		.........1<br>.........2<br>.........9	.........1<br>.........2<br>.........9	
J13	<নাম> -কে কি জন্ডিসের টিকা হেপাটাইটিস বি ইনজেকশন দেয়া হয়েছে, যা ডান উরুতে কোন কোন সময় DPT – র সাথে একই সময়ে দেয়া হয় ?Did (NAME) receive HEP B to prevent hepatitis that is given in the right thigh, sometimes given at the same time as DPT?	হ্যাঁ Yes.........1<br>না No.........2<br>জানি না/মনে নাই Don't know/CR.........9		.........1<br>.........2<br>.........9	.........1<br>.........2<br>.........9	 →J15<br>→J15
J14	<নাম> কত বার জন্ডিসের টিকা পেয়েছিল? How many times were these injections received?	কত বার How many times .... |___| বার<br>*[জানিনা হলে 9 লিখুন]*		.... |___| বার	.... |___| বার	

No	Questions and filters	Options and coding category	সর্বশেষ বাচ্চা<br>Last Birth	সর্বশেষ থেকে শুরু করে ২য় বাচ্চা<br>Next to last birth	সর্বশেষ থেকে শুরু করে ৩য় বাচ্চা<br>2nd to last birth	Skip
J15	গত 30 নভেম্বর 2011 এবং 3 জানুয়ারী 2012 এ অনুষ্ঠিত হয়ে যাওয়া জাতীয় টিকা দিবসে বা NID -তে <নাম> কে কোন পোলিও টিকা খাওয়ানো হয়েছে কি? Did your children receive any polio vaccine from the National Immunization Days (NID) on ---------	হ্যাঁ Yes<br>না No<br>জানি না/মনে নাই Don't know/CR	....1<br>..2<br>..........9	........................1<br>........................2<br>........................9	........................1<br>........................2<br>........................9	
J16	<নাম>-কে কি কখনও ভিটামিন 'এ' ক্যাপসুল খাওয়ানো হয়েছিল? *(ভিটামিন'এ ক্যাপসুল দেখান)* Has <CHILD> ever received a Vitamin A capsule (supplement) like this one - From where mustered oil like substance was squeezed out to the mouth of the child? *(Show capsule)*	হ্যাঁ Yes<br>না No<br>জানি না/মনে নাই Don't know/CR	....1<br>..2<br>..........9	........................1<br>........................2<br>........................9	........................1<br>........................2<br>........................9	 →J19<br>→J19
J17	গত 6 মাসের মধ্যে <নাম>-কে এ ধরনের ভিটামিন 'এ' ক্যাপসুল খাওয়ানো হয়েছিলো? Did <CHILD> receive a Vit A capsule (supplement) in the last 6 months?	হ্যাঁ Yes<br>না No<br>জানি না/মনে নাই Don't know/CR	....1<br>..2<br>..........9	........................1<br>........................2<br>........................9	........................1<br>........................2<br>........................9	
J18	গত 30 নভেম্বর 2008 এবং 3 জানুয়ারী 2009 এ হয়ে যাওয়া জাতীয় টিকা দিবস বা NID এবং 6 জুন 2009 এ অনুষ্ঠিত হয়ে যাওয়া ভিটামিন-'এ ক্যামপেইন এ বাচ্চাদের ভিটামিন-'এ খাওয়ান হয়েছিল <নাম> কে কি এসময় ভিটামিন' 'এ ' ক্যাপসুল খাওয়ানো হয়েছে? Was the child given Vit-A on National Immunization day or Vit-A Camp day?	NID তে খাওয়ানো হয়েছে<br>ভিটামিন-'এ ক্যামপেইন এ খাওয়ানো হয়েছে	Y .... N.. DK<br>.....1....2 ....9<br>.............1....2 9	.....Y..... N....DK<br>.....1...... 2...... 9<br>.....1...... 2...... 9	.... Y..... N....DK<br>.... 1...... 2...... 9<br>.... 1...... 2...... 9	
J19	<নাম> কে কি কখনও কৃমির ঔষধ খাওয়ানো হয়েছিল? *[নমুনা দেখান]* Has <CHILD> ever received De-Worming Tablet like this one? [*Show a sample of table]*	হ্যাঁ Yes<br>না No<br>জানি না/মনে নাই Don't know/CR	....1<br>..2<br>..........9	........................1<br>........................2<br>........................9	........................1<br>........................2<br>........................9	→K01<br>→K01
J20	গত 6 মাসে <নাম> কে কি কৃমির ঔষধ খাওয়ানো হয়েছিল? Did <CHILD> receive De-worming tablet in the last 6 months?	হ্যাঁ Yes<br>না No<br>জানি না/মনে নাই Don't know/CR	....1<br>..2<br>..........9	........................1<br>........................2<br>........................9	........................1<br>........................2<br>........................9	

Section K: Malaria Prevention

No	Questions and filters	Options and coding category	সর্বশেষ বাচ্চা<br>Last Birth<br>________	সর্বশেষ থেকে শুরু করে ২য় বাচ্চা<br>Next to last birth<br>________	সর্বশেষ থেকে শুরু করে ৩য় বাচ্চা<br>2nd to last birth<br>________	Skip
K01	<নাম> গত রাতে মশারির ভিতর ঘুমিয়েছিল কি?<br>Did <CHILD> sleep under a bednet last night?	হ্যাঁ Yes<br>না No<br>জানি না/মনে নাই Don't know/CR	....1<br>....2<br>....9	.... 1<br>.... 2<br>....9	.... 1<br>.... 2<br>.... 9	 →L01<br>→L01
K02	এই মশারিটিকে কখনো কোন কীটনাশকে চুবানো হয়েছিল কি? *[আপনাকে নিশ্চিত হতে হবে যে পরিচর্যাকারী প্রশ্নটি বুঝেছেন। প্রয়োজনে, কীটনাশক, মশা নিরোধক ইত্যাদি বুঝানোর জন্য স্থানীয় শব্দ/নাম ব্যবহার করুন]* Is the bednet ever been treated with insectiside?	হ্যাঁ Yes<br>না No<br>জানি না/মনে নাই Don't know/CR	....1<br>....2<br>....9	.... 1<br>.... 2<br>....9	.... 1<br>.... 2<br>....9	 →L01<br>→L01
K03	মশারিটি সর্বশেষ কবে কীটনাশকে চুবানো হয়েছিল? *[1 মাস পুরা না হলে 00 লিখুন]*<br>When the mosquito net was last treated with insectiside/ medicine?	মাস আগে Months ago<br>জানি না/মনে নাই Don't know/CR	.... |___|___| 99	....|___|___| 99	.... |___|___| 99	

Section L: Morbidity

No	Questions and filters	Options and coding category	সর্বশেষ বাচ্চা<br>Last Birth<br>________	সর্বশেষ থেকে শুরু করে ২য় বাচ্চা<br>Next to last birth<br>________	সর্বশেষ থেকে শুরু করে ৩য় বাচ্চা<br>2nd to last birth<br>________	Skip
	**Miscellaneous Morbidity**					
L01	কখনও কখনও ছেলেমেয়েদের কঠিন রোগ-ব্যাধি হয় এবং তখন তাদেরকে অবিলম্বে চিকিৎসার জন্য হাসপাতালে/ ডাক্তারের কাছে নিয়ে যেতে হয়। কি ধরনের লক্ষণ দেখলে আপনি আপনার শিশুকে সাথে সাথেই চিকিৎসার জন্য হাসপাতালে বা ডাক্তারের কাছে নিয়ে যাবেন? *[প্রোব করবেন না]*<br>Sometimes children have severe diseases and should be taken immediately to a health facility. When should you take a child to a health facility right away?<br>*[Do not prompt - keep asking for more signs/symptoms until the caretaker cannot recall any additional ones.]*<br>**উল্লেখ করেছেন** **উল্লেখ করেন নি**<br>যদি শিশু পানি বা বুকের দুধ খেতে না পারে Child not able to drink or breastfeed.......... 1 .... 2<br>যদি শিশু বেশি অসুস্থ হয়ে পড়ে Child becomes sicker .... 1 .... 2<br>যদি শিশুর জ্বর হয় Child develops fever... 1 .... 2<br>যদি শিশুর ঠান্ডা বা কাশির সাথে শ্বাস-প্রশ্বাস দ্রুত হয় Child has fast breathing .... 1 .... 2<br>যদি শিশুর ঠান্ডা বা কাশির সাথে শ্বাস-কষ্ট হয় Child has difficult breathing .... 1 .... 2<br>যদি শিশুর পাতলা পায়খানা বা ডাইরিয়ার সাথে রক্ত যায় Child has blood in the stool ..... 1 .... 2<br>যদি শিশু পাতলা পায়খানা বা ডাইরিয়ার সাথে পানি/দুধ কম খায় Child is drinking poorly 1 .... 2<br>যদি শিশুর খিচুনী হয় Child has convulsion .... 1 .... 2<br>যদি শিশু অজ্ঞান/নিস্তেজ হয়ে পড়ে Child become lethergis/unconscious .... 1 .... 2<br>যদি শিশু সব কিছু বমি করে দেয় Child vomit everything.................................... 1 .... 2<br>যদি শিশুর দুর্ঘটনা হয় Child has an accident.. 1 .... 2<br>যদি শিশুর ঠান্ডা বা কাশি হয় Child has cough or cold .... 1 .... 2<br>যদি শিশুর খুব পাতলা পায়খানা হয় Child has diarrhoea...................................... 1 .... 2<br>অন্যান্য *[নির্দিষ্ট করুন]* Others (specify) ____________________ |_____|_____| অন্যান্য *[নির্দিষ্ট করুন]* Others (specify) ____________________ |_____|_____|					
L02	*[যদি মা উপরের এক বা একাধিক লক্ষণ বলতে পারে, তাহলে মাকে জিজ্ঞেস করুন]-*<br>**আপনি কোথা থেকে বা কার কাছ থেকে এই তথ্য জেনেছেন?**<br>*[জিজ্ঞেস করুন]* আর কোথাও থেকে?<br>*If mother mentions one or more of the above listed danger signs, Ask.*<br>from where /who provided you with the information?	স্বাস্থ্যকর্মী:ডাক্তার/নার্স/ধাত্রী/প্যারামেডিক (Doctor/ Nurse/Midwife/Paramedics) A<br>স্বাস্থ্য কেন্দ্র / হাসপাতাল (Health facility/ Clinic/ Hospital) ....B<br>কমিউনিটি স্বাস্থ্য কর্মী - স্বাস্থ্য সহকারী/ পরিবার কল্যান সহকারী, পুষ্টি কর্মী, এনজিও কর্মী, MNCS প্রমোটর স্বেচ্ছাসেবী (CHWs -HAS , FWAs CNPs, MNCS promoter,NGO worker, volunteer)..C<br>কমিউনিটি গ্রুপ মিটিং / মিটিং /সভা থেকে (Community meeting).............D<br>রেডিও / টিভি (Radio /TV) ....E<br>সংবাদপত্র /খবরের কাগজ/বইপত্র (Newspaper/Books) ....F<br>পোষ্টার / কোন ছাপানো কাগজ (Poster/Leaflet)....................................G<br>পরিবারের অন্যান্য সদস্য/আত্মীয়/ প্রতিবেশী/বন্ধু (Family/relatives/ Neighbor or friend)...H<br>অন্যান্য Others(নির্দিষ্ট করুন) ________________________X<br>জানি না/মনে নাই...Z				
L03	কোন শিশু অসুস্থ হলে, আপনি কি তাকে স্বাভাবিকের তুলনায় বেশি খাবার খেতে দেবেন, নাকি কম খাবার দেবেন, নাকি মোটামুটি একই পরিমাণ খাবার দেবেন?<br>If any child becomes sick will you give him/her extra food than normal or less food or same as normal?	কিছুই না Nothing ....1<br>স্বাভাবিকের তুলনায় অনেক কম Much less than normal.............................2<br>স্বাভাবিকের তুলনায় কিছুটা কম Less than normal ....3<br>মোটামুটি একই পরিমাণ Same as normal..4<br>স্বাভাবিকের তুলনায় বেশি More than normal ....5<br>শিশু যেমন খেতে চায় Whatever the child wishes ....6<br>জানেন না Don't know 9				
L04	আপনার জানা মতে, আপনার বাড়ি থেকে সবচেয়ে কাছের স্বাস্থ্য কেন্দ্র কোনটি, তা কি আপনি আমাকে বলতে পারেন?<br>Can you tell me which the closest health facility to your home is?	হ্যাঁ Yes .... 1<br>না No .... 2<br>জানি না/মনে নাই Don't know/CR 9				 →L06<br>→L06
L05	স্বাস্থ্য কেন্দ্রটির নাম ঠিকানা বলুন:<br>Tell me the name and address of the health facility	________________________________			|____|____|	

No	Questions and filters	Options and coding category	সর্বশেষ বাচ্চা Last Birth	সর্বশেষ থেকে শুরু করে ২য় বাচ্চা Next to last birth	সর্বশেষ থেকে শুরু করে ৩য় বাচ্চা 2nd to last birth	Skip
	**Hospitalization History**					
L06	গত এক বছরে, অর্থাৎ গত বছরের <মাস> থেকে এখন পর্যন্ত, <নাম> কে কোন হাসপাতাল অথবা স্বাস্থ্য কেন্দ্রে ভর্তি করা হয়েছিল কি, অর্থাৎ, সেখানে রাতে থেকে যেতে হয়েছিল? In this past year, that is, from <MONTH> of last year to now, has <CHILD> been admitted to a hospital or health facility or needed to stay there for at least 24 hrs ?	হ্যাঁ Yes .... 1<br>না No .... 2<br>জানি না/মনে নাই Don't know/CR 9		.... 1<br>.............. 2<br>............... 9	.... 1<br>............... 2<br>................9	 →L09<br>→L09
L07	কয় বার <নাম> কে হাসপাতালে/স্বাস্থ্য কেন্দ্রে ভর্তি করতে হয়েছিল? Can you tell me how many times he/she was admitted in the hospital/ health facility?		|____|____| বার Times	|____|____| বার Times	|____|____| বার Times	

L08 কি কি রোগের জন্য তাকে হাসপাতালে/স্বাস্থ্য কেন্দ্রে ভর্তি করা হয়েছিল? *[প্রতিবারের জন্য আলাদা আলাদা লিখুন, ভর্তির কারণ প্রোব করুন]* Can you tell me for which diseases he/she was admitted at the hospital/health facility? *[Write separately for each admission and probe the reason for admission]*

	Last Birth ... Y .... N ..DK	Next to last birth .. Y.... N.. DK	2nd to last birth ... Y .... N ..DK
**প্রথম বার First time:**			
যে রোগের জন্য ভর্তি করা হয়েছিল তা জানা নাই বা মনে নাই Can't remember the disease for which s/he was admitted	... 1 ..... 2 9	.. 1..... 2..... 9	... 1 ..... 2 9
নিউমোনিয়া ARI/pneumonia	... 1 ..... 2 9	.. 1..... 2..... 9	... 1 ..... 2 9
ডায়রিয়া Diarrhoea	... 1 ..... 2 9	.. 1..... 2..... 9	... 1 ..... 2 9
ম্যালেরিয়া Malaria	... 1 ..... 2 9	.. 1..... 2..... 9	... 1 ..... 2 9
হাম Measles	... 1 ..... 2 9	.. 1..... 2..... 9	... 1 ..... 2 9
অপুষ্টি অথবা রক্ত স্বল্পতা Malnutrition or anaemia	... 1 ..... 2 9	.. 1..... 2..... 9	... 1 ..... 2 9
অন্যান্য Others : ______________________________	.... |____|____|	.... |____|____|	.... |____|____|
অন্যান্য Others : ______________________________	.... |____|____|	.... |____|____|	.... |____|____|
**দ্বিতীয় বার Second time:**			
যে রোগের জন্য ভর্তি করা হয়েছিল তা জানা নাই বা মনে নাই Can't remember the disease for which s/he was admitted	... 1 ..... 2 9	.. 1..... 2..... 9	... 1 ..... 2 9
নিউমোনিয়া ARI/pneumonia	... 1 ..... 2 9	.. 1..... 2..... 9	... 1 ..... 2 9
ডায়রিয়া Diarrhoea	... 1 ..... 2 9	.. 1..... 2..... 9	... 1 ..... 2 9
ম্যালেরিয়া Malaria	... 1 ..... 2 9	.. 1..... 2..... 9	... 1 ..... 2 9
হাম Measles	... 1 ..... 2 9	.. 1..... 2..... 9	... 1 ..... 2 9
অপুষ্টি অথবা রক্ত স্বল্পতা Malnutrition or anaemia	... 1 ..... 2 9	.. 1..... 2..... 9	... 1 ..... 2 9
অন্যান্য Others : ______________________________	.... |____|____|	.... |____|____|	.... |____|____|
অন্যান্য Others : ______________________________	.... |____|____|	.... |____|____|	.... |____|____|
**তৃতীয় বার Third time:**			
যে রোগের জন্য ভর্তি করা হয়েছিল তা জানা নাই বা মনে নাই Can't remember the disease for which s/he was admitted	... 1 ..... 2 9	.. 1..... 2..... 9	... 1 ..... 2 9
নিউমোনিয়া ARI/pneumonia	... 1 ..... 2 9	.. 1..... 2..... 9	... 1 ..... 2 9
ডায়রিয়া Diarrhoea	... 1 ..... 2 9	.. 1..... 2..... 9	... 1 ..... 2 9
ম্যালেরিয়া Malaria	... 1 ..... 2 9	.. 1..... 2..... 9	... 1 ..... 2 9
হাম Measles	... 1 ..... 2 9	.. 1..... 2..... 9	... 1 ..... 2 9
অপুষ্টি অথবা রক্ত স্বল্পতা Malnutrition or anaemia	... 1 ..... 2 9	.. 1..... 2..... 9	... 1 ..... 2 9
অন্যান্য Others : ______________________________	.... |____|____|	.... |____|____|	.... |____|____|
অন্যান্য Others : ______________________________	.... |____|____|	.... |____|____|	.... |____|____|

No	Questions and filters	Options and coding category	সর্বশেষ বাচ্চা Last Birth ______	সর্বশেষ থেকে শুরু করে ২য় বাচ্চা Next to last birth ______	সর্বশেষ থেকে শুরু করে ৩য় বাচ্চা 2nd to last birth ______	Skip
	**চতুর্থ বার Fourth time:**		... Y .... N .... DK	.. Y.... N.... DK	... Y .... N .... DK	
	যে রোগের জন্য ভর্তি করা হয়েছিল তা জানা নাই বা মনে নাই Can't remember the disease for which s/he was admitted		.... 1..... 2..... 9	.. 1.......2 9	... 1 ..... 2 9	
	নিউমোনিয়া ARI/pneumonia		.... 1..... 2..... 9	.. 1.......2 9	... 1 ..... 2 9	
	ডায়রিয়া Diarrhoea........		.... 1..... 2..... 9	.. 1.......2 9	... 1 ..... 2 9	
	ম্যালেরিয়া Malaria		.... 1..... 2..... 9	.. 1.......2 9	... 1 ..... 2 9	
	হাম Measles		.... 1..... 2..... 9	.. 1.......2 9	... 1 ..... 2 9	
	অপুষ্টি অথবা রক্ত স্বল্পতা Malnutrition or anaemia		.... 1..... 2..... 9	.. 1.......2 9	... 1 ..... 2 9	
	অন্যান্য Others : ______		.... |____|____|	.... |____|____|	.... |____|____|	
	অন্যান্য Others : ______		.... |____|____|	.... |____|____|	.... |____|____|	
	**Two-Week Morbidity**					
L09	আজকে **<নাম>** -এর কোন অসুখ বা স্বাস্থ্য সমস্যা আছে কি? Does the child have any illness or health problem today?	হ্যাঁ Yes.......... 1<br>না No .... 2<br>জানি না/মনে নাই Don't know/CR 9		.... 1<br>......... 2<br>......... 9	.... 1<br>......... 2<br>......... 9	 →L11<br>→L11
L10	**<নাম>** মোট কত দিন থেকে সে অসুস্থ? For how many days <NAME> is ill?	.... |____|____| দিন day		|____|____| দিন day	|____|____| দিন day	→L13
L11	গত দুই সপ্তাহে, অর্থাৎ গত সপ্তাহের আগের সপ্তাহের <বার> থেকে **<নাম>** -এর কোন অসুখ বা স্বাস্থ্য সমস্যা হয়েছিল কি? In the last two weeks, that is, since <WEEKDAY> of the week before last, has <CHILD> had any illness or health problem?	হ্যাঁ Yes.......... 1<br>না No.......... 2<br>জানি না/মনে নাই Don't know/CR 9		.... 1<br>......... 2<br>......... 9	.... 1<br>......... 2<br>......... 9	 →M01<br>→M01
L12	**<নাম>** মোট কত দিন অসুস্থ ছিল? For how many days <NAME> was ill?	.... |____|____| দিন day		|____|____| দিন day	|____|____| দিন day	
	<নাম> -এর যদি আজকে বা গত দুই সপ্তাহের মধ্যে অসুস্থ হয়ে থাকে তবে পরের প্রশ্নগুলি পূরণ করুন| Ask the following questions if the child is ill today or was ill in the previous two weeks.					
L13	<নাম> এর কি এ সকল লক্ষণ ছিল বা আছে? *[প্রোব করুন]* Did <NAME> have the following symptoms? *Prompt: Anything else?*	হ্যাঁ ..না.. জানিনা		হ্যাঁ না জানিনা	হ্যাঁ না জানিনা	
		খিঁচুনী Convulsions.......... 1 .... 2 9		1.....2 9	1.....2........9	
		সব সময় ঘুম ঘুম ভাব Always sleepy 1 .... 2 9		1.....2 9	1.....2........9	
		সব কিছু বমি করে ফেলে দিত Vomiting. 1 .... 2 9		1.....2 9	1.....2........9	
		পানি বা বুকের দুধ কম খেতে বা খেতে পারত না Not able to drink or breastfeed ....1 .... 2 9		1.....2 9	1.....2........9	
		জ্বর Fever .... 1 .... 2 9		1.....2 9	1.....2........9	
		কাশি Cough .... 1 .... 2 9		1.....2 9	1.....2........9	
		ডায়রিয়া/পাতলা পায়খানা Diarrhoea**1 .... 2 9		1.....2 9	1.....2........9	
		শ্বাস-কষ্ট Difficult breathing* .... 1 .... 2 9		1.....2 9	1.....2........9	
		কানে ব্যথা Ear pain.......... 1 .... 2 9		1.....2 9	1.....2........9	
		ক্ষুধা কম Loss of appetite .... 1 .... 2 9		1.....2 9	1.....2........9	
		সর্দি বা নাক বন্ধ Blocked/runny nose . 1 .... 2 9		1.....2 9	1.....2........9	
		গলায় ব্যথা Sore throat .... 1 .... 2 9		1.....2 9	1.....2........9	
		চোখের সমস্যা Eye problems .... 1 .... 2 9		1.....2 9	1.....2........9	
		দ্রুত শ্বাস-প্রশ্বাস Fast breathing* ...... 1 .... 2 9		1.....2 9	1.....2........9	
		সমস্ত শরীরে দানা দানা বা গুটি গুটি Generalized rash.......... 1 .... 2 9		1.....2 9	1.....2........9	
		অন্যান্য লক্ষণ Others 1______ 1 .... 2 9		1.....2 9	1.....2........9	
		অন্যান্য লক্ষণ Others 2______ 1 .... 2 9		1.....2 9	1.....2........9	
		অন্যান্য লক্ষণ Others 3______ 1 .... 2 9		1.....2 9	1.....2........9	

No	Questions and filters	Options and coding category	সর্বশেষ বাচ্চা<br>Last Birth<br>________	সর্বশেষ থেকে শুরু করে ২য় বাচ্চা<br>Next to last birth<br>________	সর্বশেষ থেকে শুরু করে ৩য় বাচ্চা<br>2nd to last birth<br>________	Skip
	[L13 এ যদি শ্বাস-কষ্ট বা দ্রুত শ্বাস-প্রশ্বাস (যুক্ত লক্ষণ)-এ কোড 1 বৃত্তায়িত করা হয়ে থাকে, তবে L14 নং প্রশ্নটি জিজ্ঞাসা করুন:]*<br>*In case of Respiratory difficulty and/or fast breathing ask question L13*					
L14	আপনি বললেন যে শিশুর <শ্বাস-কষ্ট বা দ্রুত শ্বাস-প্রশ্বাস> হয়েছিল/হয়েছে। এটি কি তার বুকের কোন সমস্যার জন্য হয়েছে/হয়েছিল না কি তার নাক বন্ধ থাকার কারনে হয়েছে/হয়েছিল?<br>You said that the child had fast breathing or difficulty in breathing. Was/were the SYMPTOMS due to a problem in the chest or a blocked nose? *(prompt)*	বুকের সমস্যার জন্য for Chest Problems.......... 1<br>বন্ধ নাকের জন্য for Blocked nose................ 2<br>উভয় Both .... 3<br>অন্যান্য Others .... 7<br>জানেন না Don't know............................. 9	............1<br>............2<br>............3<br>............7<br>............9	............1<br>............2<br>............3<br>............7<br>............9	
	*[L13 যদি ডায়রিয়া (**যুক্ত)-এ কোড 1 বৃত্তায়িত করা হলে, তবে L15 – L19 নং প্রশ্নটি জিজ্ঞাসা করুন* **In case of Diarrhoea ask question L15 to L19:]**					
L15	আপনি বললেন যে **<নাম> -এর** ডায়রিয়া/পাতলা পায়খানা হয়েছিল। সেই সময় তার কি ......<br>বার বার বমি করেছিল কি?<br>বশি বেশি পানি খেতে চেয়েছিল কি?<br>পানি/খাবার খাচ্ছিল না বা ঠিকমতো খাচ্ছিল না<br>পায়খানায় রক্ত ছিল<br>সুস্থ ছিল না/খুব অসুস্থ ছিল<br>*[প্রত্যেকটা উত্তর পড়ে শোনান এবং উত্তরগুলো বৃত্তায়িত করুন।]*<br>You said that your child had diarrhea. During the diarrhoea, did <CHILD> have...? *(prompt)*	<u>হ্যাঁ .না....জানিনা</u><br>বার বার বমি করেছিল কি?<br>Repeated vomiting ....1 .... 2 .... 9<br>বেশি বেশি পানি খেতে চেয়েছিল কি?<br>Marked thirst........................1 .... 2 .... 9<br>পানি/খাবার খাচ্ছিল না বা ঠিকমতো খাচ্ছিল না<br>Not eating/drinking well............1 .... 2 .... 9<br>পায়খানায় রক্ত ছিল Blood in stool....1 .... 2 .... 9<br>সুস্থ ছিল না/খুব অসুস্থ ছিল<br>Not getting better/sicker...........1 .... 2 9	<u>হ্যাঁ না জানিনা</u><br>.. 1.....2 ....9<br>.. 1.....2 ....9<br>.. 1.....2 ....9<br>.. 1.....2 ....9<br>.. 1.....2 9	<u>হ্যাঁ না জানিনা</u><br>.. 1.....2.....9<br>.. 1.....2.....9<br>.. 1.....2.....9<br>.. 1.....2.....9<br>.. 1.....2.....9	
L16	ডায়রিয়া/পাতলা পায়খানা চলাকালে **<নাম> -এর** (*প্রোব করুন*) দিনে কতবার পাতলা পায়খানা করেছিল?<br>সেই দিনটির কথা বলুন, যে দিন তার সবচেয়ে বেশী বার পাতলা পায়খানা হচ্ছিল<br>How many times a day he had loose motion? (The day he had the most)	|____|____| বার Times	|____|____| বার Times	|____|____| বার Times	
L17	এই অসুস্থতার সময় আপনি **<নাম> -কে** খাবার স্যালাইন বা বাসায় বানানো স্যালাইন বা জিংক সিরাপ/ ট্যাবলেট খাইয়েছিলেন কি? *[উত্তরটি লিখুন। নমুনা ORS প্যাকেট দেখান]*<br>During this last episode of diarrhoea, did (NAME) receive any of the following: *READ EACH ITEM ALOUD AND RECORD RESPONSE BEFORE PROCEEDING TO THE NEXT.*<br>*[Show the sample ORS packet]*	<u>হ্যাঁ .. না..জানিনা</u><br>ওরস্যালাইন এর প্যাকেট<br>ORS Packet (Saline packet) ....1 .... 2 .... 9<br>বাসায় বানানো স্যালাইন<br>Homemade sugar-salt-water ....1 .... 2 .... 9<br>জিংক সিরাপ Zinc Syrup ....1 .... 2 .... 9<br>জিংক ট্যাবলেট Zinc Tablet ....1 .... 2 .... 9<br>অন্যান্য Others ____________ 1 .... 2 9	<u>হ্যাঁ না জানিনা</u><br>.. 1.....2 ....9<br>.. 1.....2 ....9<br>.. 1.....2 ....9<br>.. 1.....2 ....9<br>.. 1.....2 9	<u>হ্যাঁ না জানিনা</u><br>.. 1.....2.....9<br>.. 1.....2.....9<br>.. 1.....2.....9<br>.. 1.....2.....9<br>.. 1.....2.....9	
L18	*[যদি উপরের প্রশ্নে ডায়রিয়া চলাকালে <নাম> কে খাবার স্যালাইন খাওয়ানো না হয়ে থাকে, তাহলে মাকে জিজ্ঞেস করুন]* "আপনি কি খাবার স্যালাইন যোগাড় করার চেষ্টা করেছিলেন "?<br>If in Question L16 mother mentions that the child did not receive ORS for Diarrhoea, Ask.<br>Did you try to obtain ORS?	হ্যাঁ চেষ্টা করেছিলাম কিন্তু পাইনি<br>Yes, Could not get it............................ 1<br>না No .... 2<br>জানি না/মনে নাই Don't know/CR 9	............1<br>............2<br>............9	............1<br>............2<br>............9	 <br>→L20<br>→L20
L19	আপনি কেন খাবার স্যালাইন সংগ্রহ করতে পারেন নি?<br>Why could not you obtain the ORS?	দামের কারণে Cost ....1<br>আশে পাশে কোথাও পাওয়া যায় নাই Not available locally ....2<br>অন্যান্য Others ____________ 9	..............1<br>..............2<br>..............9	..............1<br>..............2<br>..............9	
	পরের প্রশ্নগুলি (L20-L24) গত দুই সপ্তাহের মধ্যে যে কোন অসুস্থতার জন্যই প্রযোজ্য For any illness ask questions L17-L21:					

No	Questions and filters	Options and coding category	সর্বশেষ বাচ্চা<br>Last Birth	সর্বশেষ থেকে শুরু করে ২য় বাচ্চা<br>Next to last birth	সর্বশেষ থেকে শুরু করে ৩য় বাচ্চা<br>2nd to last birth	Skip
L20	আপনার মতে **<নাম> -এর** অসুখ কি সামান্য ছিল, না মোটামুটি ছিল, না কি মারাত্নক ছিল?<br>In your opinion, was <CHILD'S> disease mild, severe or so-so?	সামান্য ছিল Mild ....<br>মোটামুটি ছিল Moderate.............................<br>মারাত্নক ছিল Severe.................................<br>অন্যান্য Other : ______________ ....<br>জানেন না Doesn't know	1<br>2<br>3<br>7<br>9	.... 1<br>......... 2<br>......... 3<br>......... 7<br>......... 9	.... 1<br>......... 2<br>......... 3<br>......... 7<br>......... 9	
L21	**<নাম> -এর** যখন অসুখ চলছিল, সে কি তখন স্বাভাবিকের তুলনায় অনেক কম তরল/পানীয় (বুকের দুধ বা অন্য দুধ সহ) খেয়েছিল, নাকি স্বাভাবিকের তুলনায় বেশি খেয়েছিল, নাকি মোটামুটি স্বাভাবিক পরিমানেই খেয়েছিল?<br>During <CHILD'S> illness, did <CHILD> drink much less, about the same or more total fluids (including breast milk and formula) than usual?	কিছুই না Nothing ....<br>স্বাভাবিকের তুলনায় অনেক কম Much less ....<br>স্বাভাবিকের তুলনায় কিছু কম Less than normal ...<br>মোটামুটি স্বাভাবিকের পরিমানে About the same...<br>স্বাভাবিকের তুলানায় বেশি More than normal......<br>জানেন না Doesn't know	1<br>2<br>3<br>4<br>5<br>9	.... 1<br>......... 2<br>......... 3<br>......... 4<br>......... 5<br>......... 9	.... 1<br>......... 2<br>......... 3<br>......... 4<br>......... 5<br>......... 9	
L22	**<নাম> -এর** যখন অসুখ চলছিল, সে কি তখন স্বাভবিকের তুলনায় অনেক কম খাবার খেয়েছিল, নাকি স্বাভাবিকের তুলনায় বেশি খেয়েছিল, নাকি মোটামুটি স্বাভাবিক পরিমানেই খেয়েছিল?<br>*(শিশুটি বুকের দুধ খেলে, বুকের দুধ খাওয়ানোকে খাবার হিসেবে গণ্য করুন)*<br>During <CHILD'S> illness, did <CHILD> eat much less, about the same or more food than usual?<br>*If the child is breastfeeding, then consider it as feeding.*	কিছুইনা Nothing ....<br>স্বাভাবিকের তুলনায় অনেক কম Much less ....<br>স্বাভাবিকের তুলনায় কিছু কম Less than normal ...<br>মোটামুটি স্বাভাবিকের পরিমানে About the same...<br>স্বাভাবিকের তুলানায় বেশি More than normal......<br>জানেন না Doesn't know	1<br>2<br>3<br>4<br>5<br>9	.... 1<br>......... 2<br>......... 3<br>......... 4<br>......... 5<br>......... 9	.... 1<br>......... 2<br>......... 3<br>......... 4<br>......... 5<br>......... 9	
L23	**<নাম>** যখন অসুস্থ ছিল, আপনি তখন তার কোন চিকিৎসা করিয়েছেন / চিকিৎসা সেবা নিয়েছিলেন কি? Did you receive any treatment for this problem /complication?	হ্যাঁ Yes..<br>না No	1<br>2	.... 1<br>......... 2	.... 1<br>......... 2	 →L30
L24	**<নাম> এর কোন কোন অসুখ/** লক্ষণ এর জন্য চিকিৎসা সেবা নিয়েছিলেন?<br>*[প্রতিটি সমস্যা/জটিলতা পড়ে শোনান এবং উত্তরটি বৃত্তায়িত করুন ।]*<br>For which symptoms you received treatment?	জ্বর Fever ....<br>কাশি Cough ....<br>ডায়রিয়া/পাতলা পায়খানা Diarrhoea......<br>শ্বাস-কষ্ট Difficult breathing ....<br>দ্রুত শ্বাস-প্রশ্বাস Fast breathing ....<br>খিঁচুনী Convulsions.......................<br>সব কিছু বমি করে ফেলে দিত Vomiting...<br>পানি বা বুকের দুধ কম খেতে বা খেতে পারত না Not able to drink or breastfeed ....<br>কানে ব্যথা Ear pain........................<br>অন্যান্য লক্ষণ Others _________	হ্যাঁ.....না.....প্র. নয়<br>1 .... 2 .... 9<br>1 .... 2 .... 9<br>1 .... 2 .... 9<br>1 .... 2 .... 9<br>1 .... 2 .... 9<br>1 .... 2 .... 9<br>1 .... 2 .... 9<br>1 .... 2 .... 9<br>1 .... 2 .... 9<br>1 .... 2 9	হ্যাঁ......না..... প্র. নয়<br>1.....2........9<br>1.....2........9<br>1.....2........9<br>1.....2........9<br>1.....2........9<br>1.....2........9<br>1.....2........9<br>1.....2........9<br>1.....2........9<br>1.....2........9	হ্যাঁ..... না ... প্র. নয়<br>1.... 2........9<br>1.... 2........9<br>1.... 2........9<br>1.... 2........9<br>1.... 2........9<br>1.... 2........9<br>1.... 2........9<br>1.... 2........9<br>1.... 2........9<br>1.... 2........9	

No	Questions and filters	Options and coding category	সর্বশেষ বাচ্চা Last Birth ________	সর্বশেষ থেকে শুরু করে ২য় বাচ্চা Next to last birth ________	সর্বশেষ থেকে শুরু করে ৩য় বাচ্চা 2nd to last birth ________	Skip

L25 <নাম>-এর অসুখের জন্য কার কার কাছ থেকে আপনি চিকিৎসা করিয়েছেন / নিয়েছিলেন? [*কোন উত্তর পড়ে শোনাবেন না* | *জিজ্ঞেস করুন:*] **আরও কিছু?** যদি একাধিক স্বাস্থ্যকর্মীর কাছে যাওয়া হয়ে থাকে তাহলে **১ম** যার কাছে গিয়েছিল সেই স্বাস্থ্যকর্মীকে **১ম**, ২য় বার যার কাছে গিয়েছিল সেই স্বাস্থ্যকর্মীকে ২য় এবং ৩য়/৪র্থ /৫ম স্বাস্থ্যকর্মীদের |_____| কলামে লিখুন From whom you sought treatment for your baby? Do not read out any answers Ask: Anything else? Circle all the answers

If more than one provider was consulted to receive care for the baby, then probe for which provider was first consulted to receive care and circle the provider on column 1 (1st). Circle the

No	Provider	Last Birth	Next to last birth	2nd to last birth
	**সরকারী (Govt Health Services)**			
11	মেডিকেল কলেজ হাসপাতাল (Medical College Hospital)______________________	1 … 2 … |___|	1 … 2 … |___|	1 … 2 … |___|
12	জেলা/সদর হাসপাতাল (District /Sadar Hospital) ___________________________...	1 … 2 … |___|	1 … 2 … |___|	1 … 2 … |___|
13	মাতৃ ও শিশু স্বাস্থ্য কেন্দ্র (MCWC) _____________________________________ ..	1 … 2 … |___|	1 … 2 … |___|	1 … 2 … |___|
14	উপজেলা স্বাস্থ্য কমপ্লেক্স (UHC) ____________________________________.....	1 … 2 … |___|	1 … 2 … |___|	1 … 2 … |___|
15	ইউনিয়ন স্বাস্থ্য ও পরিবার কল্যাণ কেন্দ্র / সাব সেন্টার/আরডি (FWC/SC/RD) ____..	1 … 2 … |___|	1 … 2 … |___|	1 … 2 … |___|
16	স্বাস্থ্য সহকারী/ পরিবার কল্যান সহকারী (HA /FWA)...	1 … 2 … |___|	1 … 2 … |___|	1 … 2 … |___|
17	কমিউনিটি ক্লিনিক (Community clinic) ______________________________........	1 … 2 … |___|	1 … 2 … |___|	1 … 2 … |___|
18	সেটেলাইট ক্লিনিক/ ইপিআই কেন্দ্র (Satellite clinic/EPI centre)	1 … 2 … |___|	1 … 2 … |___|	1 … 2 … |___|
19	অন্যান্য সরকারী স্বাস্থ্য কেন্দ্র (Other Govt Health facility) ____	1 … 2 … |___|	1 … 2 … |___|	1 … 2 … |___|
	**বেসরকারী (Non Govt Health Services)**			
21	এনজিও হাসপাতাল (NGO hospital)_________________________________...	1 … 2 … |___|	1 … 2 … |___|	1 … 2 … |___|
22	এনজিও স্থায়ী স্বাস্থ্য কেন্দ্র (NGO static health centre)__________________________	1 … 2 … |___|	1 … 2 … |___|	1 … 2 … |___|
23	পুষ্টি কেন্দ্র (NNP centre)...	1 … 2 … |___|	1 … 2 … |___|	1 … 2 … |___|
24	পুষ্টি কর্মী (CNP)____.....	1 … 2 … |___|	1 … 2 … |___|	1 … 2 … |___|
25	MNCS প্রমোটর (MNCS Promoter)________________________________.....	1 … 2 … |___|	1 … 2 … |___|	1 … 2 … |___|
26	অন্যান্য কমিউনিটি স্বাস্থ্য কর্মী - এনজিও কর্মী, স্বেচ্ছাসেবী (Other CHWs - NGO worker, volunteer) ____ ...	1 … 2 … |___|	1 … 2 … |___|	1 … 2 … |___|
27	এনজিও সেটেলাইট ক্লিনিক (NGO satellite clinic)	1 … 2 … |___|	1 … 2 … |___|	1 … 2 … |___|
28	অন্যান্য বেসরকারী স্বাস্থ্য কেন্দ্র (Other NGO Health facility) ________________________________	1 … 2 … |___|	1 … 2 … |___|	1 … 2 … |___|
	**প্রাইভেট (Private Health Services)**			
31	হাসপাতাল/ ক্লিনিক (Hospital/clinic) _______________________________ ..	1 … 2 … |___|	1 … 2 … |___|	1 … 2 … |___|
32	স্বাস্থ্য কেন্দ্র /ডিসপেনসারী (Health centre/Dispensary) ____	1 … 2 … |___|	1 … 2 … |___|	1 … 2 … |___|
33	পাশ করা ডাক্তার এমবিবিএস ডাক্তার (MBBS doctor)....... বাসায় এসেছিল/ চেম্বার ___________________________________...	1 … 2 … |___|	1 … 2 … |___|	1 … 2 … |___|
34	প্যারামেডিক/মেডিকেল এসিসটেন্ট/ উপসহকারী কমিউনিটি চিকিৎসা কর্মকর্তা (Paramedic/MA/SACMO) .... বাসায় এসেছিল/ চেম্বার ____________________________________..	1 … 2 … |___|	1 … 2 … |___|	1 … 2 … |___|

No	Questions and filters	Options and coding category	সর্বশেষ বাচ্চা Last Birth	সর্বশেষ থেকে শুরু করে ২য় বাচ্চা Next to last birth	সর্বশেষ থেকে শুরু করে ৩য় বাচ্চা 2nd to last birth	Skip
	35 প্রশিক্ষণ প্রাপ্ত টিবিএ TTBA .... বাসায় এসেছিল/ চেম্বার		1 … 2 … |___|	1 … 2 … |___|	1 … 2 … |___|	
	36 প্রশিক্ষণহীন টিবিএ (ধন্নী, চাউনী, দাই) TBA .... বাসায় এসেছিল/ চেম্বার ..		1 … 2 … |___|	1 … 2 … |___|	1 … 2 … |___|	
	37 নার্স/ধাত্রী (Nurse/midwife) .... বাসায় এসেছিল/ চেম্বার		1 … 2 … |___|	1 … 2 … |___|	1 … 2 … |___|	
	38 সি এস বি এ (CSBA) .... বাসায় এসেছিল/ চেম্বার		1 … 2 … |___|	1 … 2 … |___|	1 … 2 … |___|	
	39 এলোপ্যাথী ঔষধের দোকান (Allopath drug store)		1 … 2 … |___|	1 … 2 … |___|	1 … 2 … |___|	
	40 হোমিওপ্যাথ/হোমিওপ্যাথ ঔষধের দোকান (Homeopath/Homeopath drug store)........		1 … 2 … |___|	1 … 2 … |___|	1 … 2 … |___|	
	41 আয়ুর্বেদিক চিকিৎসক / আয়ুর্বেদিক ঔষধের দোকান /হেকিম/কবিরাজ (Ayurved/ Ayurved drug store /Hekim/Kabiraj)........		1 … 2 … |___|	1 … 2 … |___|	1 … 2 … |___|	
	42 গ্রাম ডাক্তার (Village doctor) .... বাসায় এসেছিল/ চেম্বার __________		1 … 2 … |___|	1 … 2 … |___|	1 … 2 … |___|	
	43 ইমাম/ঝাড় ফুক/ওঝা (Spiritual healer) .... বাসায় এসেছিল/ চেম্বার........		1 … 2 … |___|	1 … 2 … |___|	1 … 2 … |___|	
	44 পরিবারের অন্যান্য সদস্য/আত্মীয়/ প্রতিবেশী/বন্ধু (Family/relatives/Neighbor or friend) .... বাসায় এসেছিল/ চেম্বার........		1 … 2 … |___|	1 … 2 … |___|	1 … 2 … |___|	
	51 অন্যান্য Others ____________... বাসায় এসেছিল/ চেম্বার .... (নির্দিষ্ট করুন)		1 … 2 … |___|	1 … 2 … |___|	1 … 2 … |___|	
	99 জানি না/মনে নাই........		1 … 2 … |___|	1 … 2 … |___|	1 … 2 … |___|	
L26	*[উপরে উল্লেখিত স্বাস্থ্যকেন্দ্রগুলোর ভিতর যেগুলোতে অসুস্থ বাচ্চার সেবা বা চিকিৎসা গ্রহন করা হয়েছিল, তার ভিতর কোনটিতে কোনটিতে IMCI ট্রেনিং প্রাপ্ত স্বাস্থ্যকর্মী আছে তা চিহ্নিত করুন।]*		1 2 3 4 5	1 2 3 4 5	1 2 3 4 5	
L27	এই অসুস্থ বাচ্চার চিকিৎসা করানোর ব্যাপারে কেউ আপনাকে পরামর্শ দিয়েছিল কি? Did anyone advise you or refer you to seek care for your complication?	হ্যাঁ Yes........ 1<br>না No .... 2<br>জানি না/মনে নাই Don't know/CR........ 9	.... 1<br>......... 2<br>......... 9	.... 1<br>......... 2<br>......... 9	.... 1<br>......... 2<br>......... 9	 →M01<br>→M01
L28	চিকিৎসা নেয়ার জন্য কে আপনাকে পরামর্শ দিয়েছিল? Who had advised you or refered you to seek care for your complication?					
	<u>দক্ষ/প্রশিক্ষণ প্রাপ্ত স্বাস্থ্য কর্মী **(Medically trained)**</u>					
	পাশ করা ডাক্তার (MBBS doctor)........		.... A	.... A	.... A	
	নার্স/ধাত্রী (Nurse/midwife)........		.... B	.... B	.... B	
	প্যারামেডিক/মেডিকেল এসিসটেন্ট/ উপসহকারী কমিউনিটি চিকিৎসা কর্মকর্তা (Paramedic/MA/SACMO)........		 C	 C	 C	
	পরিবার কল্যাণ পরিদর্শক (FWV)........		.... D	.... D	.... D	
	কমিউনিটি দক্ষ দাই (CSBA)........		.... E	.... E	.... E	
	<u>অন্যান্য স্বাস্থ্য কর্মী **(Other health worker)**</u>					
	স্বাস্থ্য সহকারী/ পরিবার কল্যান সহকারী (HA /FWA)........		.... F	.... F	.... F	
	পুষ্টি কর্মী (CNP)........		.... G	.... G	.... G	
	MNCS প্রমোটর (MNCS Promoter)........		.... H	.... H	.... H	
	অন্যান্য কমিউনিটি স্বাস্থ্য কর্মী - এনজিও কর্মী, স্বেচ্ছাসেবী (Other CHWs - NGO worker, volunteer) __________		 I	 I	 I	
	<u>অন্যান্য (Other)</u>					
	প্রশিক্ষণ প্রাপ্ত টিবিএ TTBA........		.... J	.... J	.... J	
	প্রশিক্ষণহীন টিবিএ (ধন্নী, চাউনী, দাই) TBA(Dai/Dhorni/Chauni)........		.... K	.... K	.... K	
	হোমিওপ্যাথ/হোমিওপ্যাথ ঔষধের দোকান (Homeopath/Homeopath drug store)........		 L	 L	 L	
	আয়ুর্বেদিক চিকিৎসক / আয়ুর্বেদিক ঔষধের দোকান /হেকিম/কবিরাজ (Ayurved/ Ayurved drug store /Hekim/Kabiraj)........		.... M	.... M	.... M	
	গ্রাম্য ডাক্তার (Village doctor)........		.... N	.... N	.... N	
	এলোপ্যাথী ঔষধের দোকান (Allopath drug store)........		.... O	.... O	.... O	
	ইমাম/ঝাড় ফুক/ওঝা (Spiritual healer)........		.... P	.... P	.... P	
	পরিবারের অন্যান্য সদস্য/আত্মীয়/ প্রতিবেশী/বন্ধু (Family/relatives/Neighbor or friend)........		.... Q	.... Q	.... Q	
	অন্যান্য **Others** __________........ (নির্দিষ্ট করুন)		 X	 X	 X	
	জানি না/মনে নাই		.... Z	.... Z	.... Z	

No	Questions and filters	Options and coding category	সর্বশেষ বাচ্চা Last Birth	সর্বশেষ থেকে শুরু করে ২য় বাচ্চা Next to last birth	সর্বশেষ থেকে শুরু করে ৩য় বাচ্চা 2nd to last birth	Skip
L29	(নাম)-এর এই সমস্যা/জটিলতার জন্য কাউকে দেখানো বা চিকিৎসার ব্যাপারে যিনি আপনাকে পরামর্শ করেছিলেন, তিনি কি আপনাকে কোন কাগজ (রেফারেল স্লিপ) দিয়েছিলেন? *[রেফারেল স্লিপের একটি নমুনা দেখান]*<br>Did the person who had referred you issue you a referral slip/ write something on a paper to show it to the health facility/carer? *(show the referral slip to the respondent)*	হ্যাঁ Yes... 1<br>না No .... 2<br>জানি না/মনে নাই Don't know/CR 9	.... 1<br>......... 2<br>......... 9	.... 1<br>......... 2<br>......... 9	→M01<br>→M01<br>→M01
L30	আপনি কেন <নাম> -এর অসুস্থতার জন্য কোন চিকিৎসা গ্রহন করেন নি?<br>*[উত্তর গুলো পড়ে শুনাবেন না]*<br>*[উত্তরগুলো পড়ে শোনাবেন না। জিজ্ঞাস করুন:]* আরো কোন কারন ছিল?<br>*[সবগুলো উত্তরই বৃত্তায়িত করুন]*<br>Why did you not seek care for this complication?<br>Do not read out the answers<br>Ask: Anything else?<br>Circle all the answers	সেবার প্রয়োজন আছে বলে মনে হয় নি/ সেবার প্রয়োজন নেই... A<br>জানতাম না কোথায় যেতে হবে .... B<br>অনেক খরচ/ টাকা পয়সা ছিল না .... C<br>স্বাস্থ্য কেন্দ্র বাসা হতে অনেক দুরে .... D<br>যানবাহনের সমস্যা..E<br>সাথে যাবার মত কেউ ছিল না.............................F<br>স্বাস্থ্যকেন্দ্রে যাবার মত সময় ছিল না .... G<br>পরিবার আমাকে যেতে দেন নি .... H<br>ধর্মীয় মানা/বাধা..I<br>স্বাস্থ্যকেন্দ্রের সেবাদানের সময় সীমা সুবিধাজনক নয় ....J<br>স্বাস্থ্যকেন্দ্র বন্ধ ছিল/কোন স্বাস্থ্যকর্মী ছিলেন না.......... K<br>স্বাস্থ্যকেন্দ্রে সেবা অনুন্নত মানের..........................L<br>স্বাস্থ্যকেন্দ্রে নিম্নমানের এবং অদক্ষ সেবাপ্রদানকারী.....M<br>স্বাস্থ্যকেন্দ্রে পর্দার (শালীনতা) অভাব.................... N<br>স্বাস্থ্যকেন্দ্রের সেবাদানকারীদের ব্যবহার খারাপ .... O<br>স্বাস্থ্যকেন্দ্রের অনেকক্ষন বসে থাকতে হয় সেবা পাবার জন্য ....P<br>স্বাস্থ্যকেন্দ্রের ঔষধ পত্র পাওয়া যায়না.................... Q<br>অন্যান্য Others ____________________... X	.........A<br>.........B<br>.........C<br>.........D<br>......... E<br>......... F<br>.........G<br>.........H<br>.......... I<br>..........J<br>.........K<br>......... L<br>........M<br>.........N<br>.........O<br>......... P<br>.........Q<br>.........X	.... A<br>.........B<br>.........C<br>........ D<br>.........E<br>......... F<br>........ G<br>........ H<br>.......... I<br>..........J<br>........ K<br>.........L<br>........M<br>........ N<br>........ O<br>......... P<br>........ Q<br>........ X	
	সাক্ষাৎকার গ্রহনকারীর জন্য নির্দেশাবলী:<br>*গত ২ সপ্তাহে অসুস্থ প্রত্যেক শিশুর জন্য আলাদা আলাদা CHILD ILLNESS COST MODULE (MODULE VIII) পূরণ করতে হবে।*<br>*Cover Page-এ পাঁচ বছরের কম বয়সের অসুস্থ শিশুর সংখ্যা লিখুন।*					

Section M: Community Action Group

This section contains some information about community action group

আপনি হয়ত জানেন যে কখনও কখনও এলাকা ভিত্তিক কিছু কমিটি/**দল থাকে**। আমি এখন এসব বিষয়ে কিছু প্রশ্ন করব।

You may know that there are local networks and committees in many communities. Now I will be discussing about such formations in your communities.

	QUESTIONS AND FILTERS	CODING CATEGORIES	SKIP
M01	আপনার এলাকায় এমন কোন কমিটি বা দল (গ্রুপ) সম্পর্কে আপনি জানেন কি যারা মা ও বাচ্চার শারিরীক উন্নতির জন্য কাজ করে?<br>Do you know about any committees, or network or group in your community that works towards improving you or your babies' health?	হ্যাঁ Yes ....1<br>না No....2	 →M11
M02	আপনি বা আপনার পরিবারের কোন সদস্য কি এই ধরনের কমিটি বা দল (গ্রুপ) এর সদস্য?<br>Are you and/or any of your family member is a member of any such group?	হ্যাঁ Yes ....1<br>না No....2	 →M04
M03	আপনি/আপনার পরিবারের সদস্য কতদিন আগে ঐ কমিটি বা দলের (গ্রুপের) সদস্য হয়েছেন? [বছরে বললে মাসে পরিবর্তিত করুন। ১ মাসের কম হলে ০০ লিখুন]<br>How long ago did you or someone in your family become a member of this group?<br>Interviewer: If respondent answer in years convert it into months.<br>If less than one month write '00' in the box	মাস MONTH ....|___|___| মনে নাই Don't Remember....99	
M04	এই কমিটি বা গ্রুপ কোন কোন বিষয় নিয়ে কাজ করে?<br>What issues do they deal with?	মায়ের স্বাস্থ্য সেবা Mother's health ....A<br>শিশুর স্বাস্থ্য সেবা Baby's health ....B<br>পরিবার পরিকল্পনা Family planning ....C<br>শিক্ষা Education ....D<br>অসুস্থ মা ও বাচ্চার পরিবহনের ব্যবস্থা করে Transporting sick mother & babies....E<br>অসুস্থ মা ও বাচ্চার অর্থের ব্যবস্থা করে Financing sick mother & babies ....F<br>অন্যান্য Others__________ .X<br>নির্দিষ্ট করুন<br>জানে না Don't know ....Y	
M05	এই কমিটি /দলের কাছে কি আপনি কোন প্রকার সাহায্যের জন্য গিয়েছিলেন?<br>Did you go for any sort of help from this group for this/these __ problems? *[MENTION maternal and childhood problems described earlier]*	হ্যাঁ Yes ....1<br>না No 2	
M06	এই কমিটি/দলের কাছ থেকে কখনও কোন ধরনের সাহায্য পেয়েছিলেন কি?<br>Have you received any sort of help from this group?	হ্যাঁ Yes ....1<br>না No 2	 →M11
M07	এই কমিটি/দলের কাছ থেকে কি ধরনের সাহায্য পেয়েছিলেন?<br>What kind of support you and/or a newborn get from this village group/CAG?	পরিবহনের ব্যবস্থা Transport support ....A<br>টাকা পয়সার ব্যবস্থা Financial support ....B<br>স্বাস্থ্যকর্মীকে জানানো Informed health worker....C<br>স্বাস্থ্যকর্মীকে পরিদর্শন নিশ্চিত করা Ensured visit of health worker...D<br>অন্যান্য Other __________...X	 <br>→M10<br>→M10<br>→M10
M08	***[M07 এ A বৃত্তায়িত হলে]*** এই কমিটি/গ্রুপের কাছ থেকে কি ধরনের পরিবহন সুবিধা পেয়েছিলেন?<br>What kind of transport support you got from them?	পরিবহন খুঁজতে সাহায্য করেছে Helped to find a transport.. 1<br>পরিবহন দিয়েছে Provided with a transport .... 2<br>প্রজোয্য নয় Not applicable 3	
M09	***[M07 এ B বৃত্তায়িত হলে]*** তাদের তহবিল থেকে সরাসরি আপনাকে টাকা দিয়েছিল না-কি এলাকা থেকে টাকা তুলে দিয়েছিল?<br>Did they give you money directly from their fund or arranged money from the community?	তহবিল থেকে দিয়েছিল Gave money from their fund .... 1<br>এলাকা থেকে টাকা তুলো দিয়েছিল Arranged from community .2<br>জানি না Don't know .... 9<br>প্রজোয্য নয় Not applicable 3	
M10	এই কমিটি/দলের কাছ থেকে যে সাহায্য পেয়েছিলেন তা কি আপনার কাছে উপকারী মনে হয়েছে?<br>Do you consider this as beneficial?	হ্যাঁ Yes ....1<br>না No 2	
M11	*[উত্তরদাতার কাছ থেকে বিদায় নেয়ার পূর্বে প্রশ্নমালাটি ভাল করে পরীক্ষা করে দেখুন অতঃপর উত্তরদাতাকে ধন্যবাদ জানিয়ে সাক্ষাৎকার শেষ করুন][Before leaving the place check the questionnaire properly then END THE SURVEY WITH THANKS and record the time.]*	সাক্ষাৎকার শেষ করার সময় Interview ending time<br>|___|___|:|___|___| H H : M M (ঘন্টা:মিনিট)	
